# Systematic review and meta-analysis of diagnostic studies of proximal surface caries

**DOI:** 10.1007/s00784-021-04113-1

**Published:** 2021-09-04

**Authors:** Mila Janjic Rankovic, Svetlana Kapor, Yegane Khazaei, Alexander Crispin, Ina Schüler, Felix Krause, Kim Ekstrand, Stavroula Michou, Florin Eggmann, Adrian Lussi, Marie-Charlotte Huysmans, Klaus Neuhaus, Jan Kühnisch

**Affiliations:** 1 Department of Orthodontics and Dentofacial Orthopedics, University Hospital, Ludwig-Maximilians University Munich, Munich, Germany; 2 Department of Conservative Dentistry and Periodontology, University Hospital, Ludwig-Maximilians University Munich, Munich, Germany; 3 Institute of Medical Biometry and Epidemiology, Ludwig-Maximilians University of Munich, Munich, Germany; 4 Department of Orthodontics, Section of Preventive and Paediatric Dentistry, University Hospital, Jena, Germany; 5 Clinic for Operative Dentistry, Periodontology and Preventive Dentistry, University Hospital RWTH Aachen, Aachen, Germany; 6 Department of Odontology, University of Copenhagen, Copenhagen, Denmark; 7 Clinic of Periodontology, Endodontology and Cariology, University Centre for Dental Medicine Basel, University of Basel, Basel, Switzerland; 8 Department of Operative Dentistry and Periodontology, Faculty of Dentistry, University Medical Centre, Freiburg, Germany; 9 School of Dental Medicine, University of Bern, Bern, Switzerland; 10 Department of Dentistry, Radboud University Medical Centre, Nijmegen, The Netherlands; 11 Department of Dermatology, Inselspital - Bern University Hospital, Bern, Switzerland; 12 Poliklinik für Zahnerhaltung und Parodontologie, Klinikum der Universität München, LMU München, Goethestraße 70, 80336 Munich, Germany

**Keywords:** Approximal caries, Proximal caries, Interproximal caries, Caries detection, Caries diagnostics, Visual examination, Bitewing radiography, Laser fluorescence measurements, Fibre-optic transillumination, Systematic review, Meta-analysis, Diagnostic performance, Diagnostic accuracy, Sensitivity, Specificity

## Abstract

**Aim:**

This systematic review and meta-analysis aimed to assess the diagnostic accuracy and reliability of commonly used caries detection methods for proximal caries diagnostics. Visual examination (VE), bitewing radiography (BWR), laser fluorescence (LF), and fibre-optic transillumination (FOTI) were considered in detail.

**Material and methods:**

PRISMA guidelines for the reporting of systematic reviews and meta-analyses were applied. The mnemonic PIRDS (problem, index test, reference test, diagnostic and study type) concept was used to guide the literature search. Next, studies that met the inclusion criteria were stepwise selected and evaluated for their quality with a risk of bias (RoB) assessment tool. Studies with low/moderate bias and sufficient reporting were considered for meta-analysis. The pooled sensitivity (SE), specificity (SP), diagnostic odds ratio (DOR), and area under the ROC curve (AUC) were calculated.

**Results:**

From 129 studies meeting the selection criteria, 31 in vitro studies and five clinical studies were finally included in the meta-analysis. The AUC values for in vitro VE amounted to 0.84 (caries detection) and 0.85 (dentin caries detection). BWR ranged in vitro from 0.55 to 0.82 (caries detection) and 0.81–0.92 (dentin caries detection). LF showed higher AUC values for overall caries detection (0.91) and dentin caries detection (0.83) than did other methods. Clinical data are limited.

**Conclusion:**

The number of diagnostic studies with low/moderate RoB was found to be low and indicates a need for high-quality, well-designed caries diagnostic studies.

**Clinical relevance:**

BWR and LF showed good diagnostic performance on proximal surfaces. However, because of the low number of includable clinical studies, these data should be interpreted with caution.

**Supplementary Information:**

The online version contains supplementary material available at 10.1007/s00784-021-04113-1.

## Introduction

Many studies have indicated a decline in caries prevalence [1, 2]. However, the occurrence of proximal caries lesions in posterior teeth is still very common in primary and permanent dentition and should not be underestimated [3]. For this reason, the detection, assessment, and diagnostics of proximal caries lesions is an important procedure for clinicians in daily dental practice and should enable well-justified preventive, non-operative, or operative caries management [4–6]. When considering visual examination (VE) as a basic diagnostic method, it must be concluded that this technique is generally insufficient to estimate lesional characteristics in terms of detecting early lesions and determining the caries extent or activity at proximal sites [7–11]. Therefore, conventional, film-based bitewing radiographs (conv-BWR) were introduced as an additional diagnostic method of first choice several decades ago [12] and are still used mostly through digital bitewing radiography (dig-BWR) in daily clinical routines [13, 14]. To improve the repeatability of diagnostic examinations and provide X-ray–free diagnostics, several other photo-optical methods have been introduced over the last few decades, and these modalities, e.g. laser fluorescence (LF, DIAGNOdent, KaVo, Biberach, Germany) or fibre-optic transillumination (FOTI), can potentially be used on proximal sites [14–16].

Over the past decades, many in vitro and in vivo studies on proximal caries have assessed the diagnostic performance of the abovementioned methods. Meanwhile, systematic reviews have summarised the existing data [13, 16–23]. However, when analysing these studies in detail, it becomes evident that there is considerable variation in the results, which is probably linked to variations in the chosen methodology, e.g. different study aims, differences in the usage of the index and reference test method, different thresholds to determine the caries process or technical differences in the performance of each study. All of these aspects might limit the comparability between the studies. Even though the available systematic reviews [13, 17, 18] have mentioned substantial heterogeneity between the included diagnostic studies, little attention has been paid to this important methodological issue so far, and therefore, potential methodological sources of bias might be undetected and may also potentially skew the meta-analytic data. Ideally, each diagnostic trial should be designed similarly according to equal scientific standards and protocols to generate comparable results and, therefore, decrease the potential risk of bias (RoB) and exhibit low heterogeneity.

Therefore, the primary objective of this report was to assess and compare the diagnostic performance of commonly used methods for proximal caries detection under in vitro and in vivo conditions in permanent, posterior teeth. To achieve this aim, it was necessary first to identify relevant studies on the basis of a systematic search of the literature, second, to evaluate potential sources of bias, and third, to provide meta-analytic data of the diagnostic accuracy.

## Material and methods

To support the unbiased inclusion of studies and reporting of findings, this systematic review was conducted according to the PRISMA-DTA statement (Preferred Reporting Items for a Systematic Review and Meta-Analyses of Diagnostic Test Accuracy Studies) [24]. Additionally, most recently published drafts of the “Cochrane Handbook for Diagnostic Test Accuracy Reviews” [25] and “The Joanna Briggs Institute Reviewers’ Manual 2015: Methodology for JBI Scoping Reviews” [26] influenced this work. The systematic review was registered on the PROSPERO platform (CRD42017069894).

### Inclusion and exclusion criteria

Studies eligible for inclusion were in vivo and in vitro caries diagnostic studies that tested the diagnostic performance of the following caries diagnostic methods: (1) VE with and without tactile examination, (2) conventional bitewing radiography (conv-BWR) independently from the film type used, (3) digital bitewing radiography (dig-BWR), (4) laser fluorescence measurement (LF, DIAGNOdent 2095 and 2190; KaVo, Biberach, Germany), and (5) fibre-optic transillumination with (FOTI, I.C. Lercher, Emmingen, Germany). Only studies assessing primary caries on the proximal surfaces of permanent posterior teeth were considered for inclusion. Studies containing information on primary teeth or teeth with restorations, secondary caries, or artificially induced caries lesions were excluded. The actual status of the tooth surface had to be confirmed by a suitable reference test. In in vitro studies, histological validation of dental tissues was considered the “gold standard,” while in in vivo studies, this validation was direct VE after tooth separation or “bioptical” cavity preparation. In order to be included, at least one of the following outcomes had to be assessed: diagnostic test accuracy (expressed in terms of sensitivity (SE), specificity (SP), AZ values from ROC curves, and/or reliability/reproducibility (Kappa). Only studies published in English until 31 December 2018 were considered for inclusion.

### Development of the search strategy

In relation to the above-formulated research question and the corresponding inclusion and exclusion criteria, a structured search of the literature was initiated in accordance with the mnemonic PIRD recommendations [27]. The final consented search items are shown in Table 1.

**Table 1:** Search strategy and documentation of keywords according to the PIRDS concept [27]

Population/Problem (*P*)		Index test (*I*)		Reference test (*R*)		Diagnostic and study type (*D*/*S*)
caries decay AND proximal approximal interdental	AND	visual clinical* inspect* ICDAS bitewing conventional digital film radiogra* analo* speed* X ray Xray radiol* roentge* laser fluorescence diagnodent foti difoti fiber fibre transillumination opti* qlf quantit* laser light induced	AND	valid* accuracy sensitivity specificity SE SP ROC Az reproducib* reliab* Kappa threshold cut-off performance histolog* micro micro computed CT *CT	AND	Systemat* Meta-Analysis Diagnos* Detect* Assessm* Vivo Vitro Study Studies
MeSH terms that were used to search the PubMed and EMBASE databases: ((caries OR decay) AND (proximal OR approximal OR interdental) AND (visual OR clinical* OR inspect* OR icdas OR bitewing OR conventional OR digital OR film OR radiogra* OR analo* OR speed* OR X ray OR Xray OR radiol* OR roentge* OR laser OR fluorescence OR diagnodent OR foti OR difoti OR fiber OR fibre OR transillumination OR opti* OR qlf OR quantit* OR laser OR light OR induced) AND (OR valid* OR accuracy OR sensitivity OR specificity OR se OR sp OR roc OR az OR reproducib* OR reliab* OR kappa OR threshold OR cutoff OR perfORmance OR histolog* OR micro OR micro computed OR ct OR *ct) AND (systemat* OR review OR meta-analysis OR diagnos* OR detect* OR assessm* OR vivo OR vitro OR study OR studies))						

### Literature search and study selection process

**Fig. 1:**
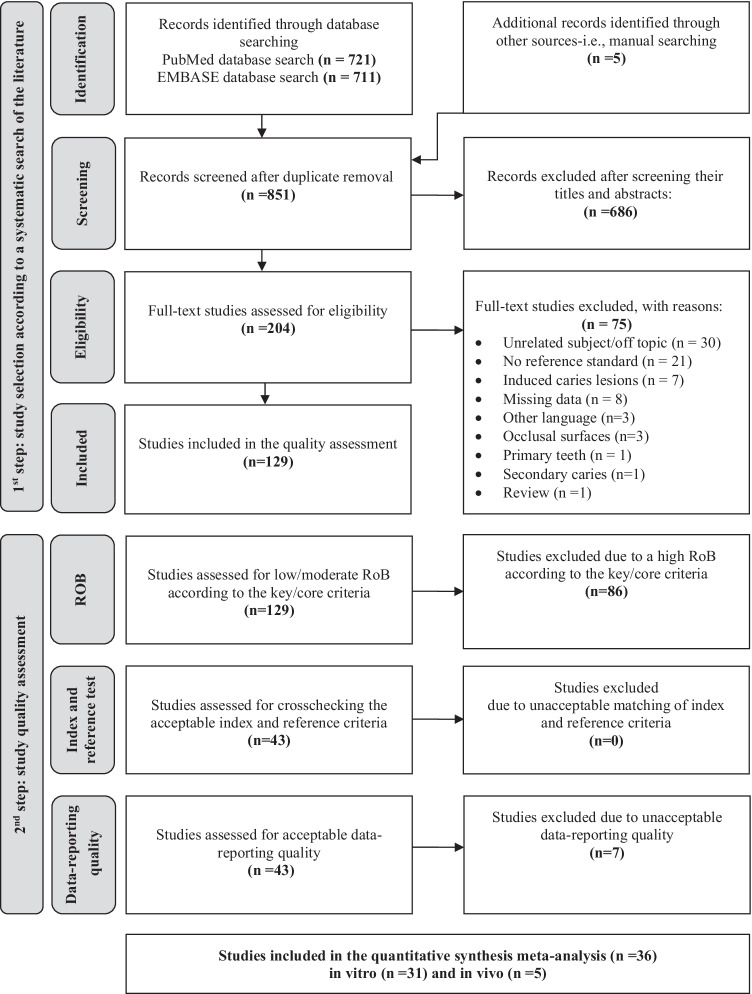
Flow diagram detailing our search and study selection process applied during the systematic literature search (1st step) and study quality assessment (2nd step)

A literature search was performed in the MEDLINE (PubMed) and EMBASE databases following the predefined search strategy (Figs. 1, Table 1). The electronic search yielded 721 abstracts from PubMed and 711 abstracts from EMBASE. Both sets of records were downloaded from each database to the bibliographic software package EndNote X7 (Clarivate Analytics, Philadelphia, PA, USA) and merged into one core database to remove duplicate records and to facilitate retrieval of relevant articles. All potentially relevant reports identified after searching other nonelectronic sources were entered into EndNote manually. After the elimination of duplicates, 851 studies were identified. Additionally, five new studies were identified through other sources (Fig. 1).

The titles and abstracts of all identified studies were examined by two reviewers independently (M.J.R and S.K.), according to predefined inclusion and exclusion criteria. Review authors were not blinded to the names of the authors, institutions, journal of publication, or results of the studies. All records identified by the searches were primarily checked on the basis of the title and abstract. Records that were obviously irrelevant were excluded, and the full text of all remaining records was obtained. If the relevant information for meeting the inclusion criteria was not available from the abstract and/or title, we obtained the full text of the report. In this way, 204 studies were selected for full-text reading and were assessed independently by the same two reviewers. Any doubts or disagreements were solved by discussion with an experienced researcher (J.K.). Articles that did not meet all inclusion criteria after the full-text assessment (*N* = 75) were excluded from further examination. Reasons for their exclusion were recorded in specially prepared tables (Supplemental Table S0). Figure 1 depicts and summarises the complete study selection process.

### Data extraction

Data from the included studies were extracted by both reviewers (M.J.R. and S.K.) using a structured examination form. Any disagreements were resolved through discussion with an expert (J.K.) until a consensus was reached. Trial authors were contacted for clarification or missing information, where necessary. In brief, the following information was extracted from the papers: (1) the setting of in vivo or in vitro studies; (2) study material details, including the number of patients, age, type, and the number of teeth used in the investigation; (3) diagnostic criteria and methodology of the index and reference standard including cutoff values (Supplementary Table S1a-e); and (4) diagnostic-accuracy results (SE, SP, Az value, inter- and, intra-examiner reliability). All extracted data are summarised in tables and can be obtained from the supplementary online content on the journal website (Supplementary Tables S3a-d, S4a–d, S5a–d, S6a–d, and S7a–d).

### RoB assessment and study selection for meta-analysis

For this study project, a new, tailor-made RoB assessment tool was used (Supplementary Table S2). Briefly, the tool consists of four domains, each of them containing items that cover different sources of bias. To determine the RoB in the primary studies, one of three modalities was used, high, low, or unclear. The category “unclear RoB” was used whenever no information or insufficient details were reported by the study group. The RoB assessment was performed independently by two reviewers (M.J.R., S.K.). An additional reassessment was performed by two other colleagues from the workgroup (I.S., F.K.).

To choose studies with a low RoB for the meta-analysis, an additional selection step was performed by checking the study quality. Studies that were found to be related to a low/moderate RoB in the key items (index test criteria, reference test criteria, incorporation bias, partial verification bias, and differential verification bias) were included in the meta-analysis. In the case of in vivo studies, differential verification bias was not considered a key item, since using two different reference tests for different caries thresholds can be justified for ethical reasons (e.g. “bioptical” cavity preparation not applicable in all cases). In a second selection step, each study report was carefully cross-checked again if the index and reference test criteria and the corresponding thresholds were correctly used. The final inclusion was made when the quality of data reporting was found to be sufficient. At least 2 × 2 contingency tables or the SE, SP, negative predictive value (NPV), and positive predictive value (PPV), which could be used in the meta-analysis, had to be reported. The RoB assessment of all systematically searched and selected studies was performed independently by 2 reviewers (M.J.R and S.K.); discrepancies were resolved again in cooperation with an experienced researcher (J.K.).

### Data handling, statistical procedures, and meta-analysis

All data were entered into a database and later transferred to Excel spreadsheets (Excel 2010, Microsoft Corporation, Redmond, WA, USA). Descriptive data analyses were performed using Microsoft Excel 2010 and the statistical package mada version 0.5.9. [28] for RStudio [29]. If the included studies provided contingency tables, the data were used directly. If not, we calculated true positives (SE), true negatives (SP), false positives, and false negatives from the given data in the original publication. If these calculations were not possible, the corresponding study was excluded. Corrections of tables with zero cells were also made; when, for example, TP is zero, R itself makes a correction by changing the zero to 0.5 (a very small number) because RStudio cannot deal with zero cells. In some reports, statistical information was given to more than one examiner. However, in those cases, a mean was calculated by logit transformation.

Meta-analytic statistics were calculated for all included diagnostic test methods and commonly used diagnostic thresholds. Diagnostic accuracy and their 95% confidence intervals (95% CI) were calculated from the pooled data of all included studies, in terms of SE, SP, and the diagnostic odds ratio (DOR). A bivariate diagnostic random-effects meta-analysis suggested by Reitsma et al. [30] was used to provide pooled estimates of SE and SP for the respective subgroups along with their 95% CI. This method can take the heterogeneity between studies into account by jointly analysing the logit transformation of SEs and SPs [31]. Finally, the pooled DOR was calculated using a random-effects model following the approach by DerSimonian and Laird and aimed at describing the performance of the included diagnostic tests [32]. An uninformative test shows a DOR value of 1; as the DOR increases, the test has more discriminatory power [33]. The area under the curve (AUC) of summary receiver operating characteristics (sROC) was reported to create an overall view of the results within each subgroup. The AUC value quantifies the overall ability of a diagnostic test to discriminate between individuals with the disease and those without the disease [34]. The ideal test would have an AUC value of 1, whereas a random guess would have an AUC of 0.5; the larger the area under the ROC curve, the more accurate the diagnostic test [33]. In addition, sROC plots and forest plots were computed to illustrate the diagnostic performance and heterogeneity, respectively [34].

## Results

**Fig. 2:**
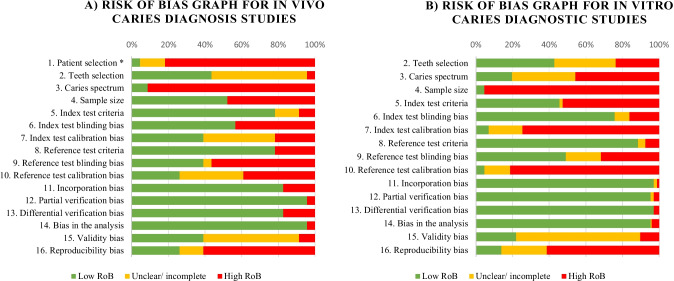
RoB graph across included in vivo (**a**) and in vitro (**b**) caries diagnostic studies for proximal surfaces. *Item no. 1 (patient selection bias) is only available for clinical diagnostic studies

Altogether, 129 studies were accounted for after meeting the inclusion criteria in the first selection step (Fig. 1, Table 2); 120 were performed under in vitro conditions and 9 under in vivo conditions. When additionally considering those studies with a low/moderate RoB (Fig. 2), the number of includable studies decreased to 43. Furthermore, 7 studies had to be excluded due to the low quality of data reporting. Finally, 31 laboratory studies [35–65] and five clinical studies [66–70] were included in the meta-analysis. Figure 1 and Table 2 provide a summary of the step-by-step selection process. All details of the systematic search of the literature and the stepwise selection process before meta-analysis can be taken from the supplementary online content.

**Table 2:** Overview of the identified diagnostic studies in relation to the method used and characteristics of the study setup with stepwise included studies for meta-analysis

Studies on diagnostic methods	1st step		2nd step		
	Study inclusion according to the systematic search of the literature		Study inclusion according to the quality assessment		
	Study setup	Specification (*N* according to PRISMA)	Low/moderate RoB	Acceptable index and reference test	Acceptable data reporting quality
VE (*N* = 20)	In vitro (*N* = 15)		10	10	10
	In vivo (*N* = 5)		2	2	2
Conventional bitewing radiography (*N* = 77)	In vitro (*N* = 72)	D-speed (*N* = 17)	24	24	19
		E-speed (*N* = 43)			
		F-speed (*N* = 26)			
		Not specified (*N* = 4)			
	In vivo (*N* = 5)	D-speed (*N* = 0)			
		E-speed (*N* = 5)	1	1	1
		F-speed (*N* = 0)			
Digital bitewing radiography (*N* = 88)	In vitro (*N* = 81)	Phosphor plate (*N* = 46)	23	23	19
		Sensor (*N* = 48)			
	In vivo (*N* = 7)	Phosphor plate (*N* = 3)	4	4	3
		Sensor (*N* = 4)			
LF measurement (*N* = 9)	In vitro (*N* = 5)	DIAGNOdent 2095 (*N* = 1)	5	5	4
		DIAGNOdent 2190/pen (*N* = 4)			
	In vivo (*N* = 4)	DIAGNOdent 2095 (*N* = 0)	4	4	4
		DIAGNOdent 2190/pen (*N* = 4)			
Fibre-optic transillumination (*N* = 7)	In vitro (*N* = 4)	-	-	-	
	In vivo (*N* = 3)	-	-	-

The majority of the included studies assessed the diagnostic accuracy of conventional and digital BWR, and only a few of them additionally assessed VE and LF (Table 2). Table 3 provides an overview of the meta-analytic diagnostic accuracy for each diagnostic method, diagnostic threshold, and study setting. The results from the clinical studies are partial and mostly limited to the dentin detection level, showing only data based on a few studies. According to this assessment, digital, sensor-based BWR showed the highest SE value for dentin detection level, of 0.96, followed by phosphor plate-based BWR (0.83), LF (SE = 0.63), E-speed BWR (0.35), and VE (SE = 0.32) (Table 3).

**Table 3:** Bivariate diagnostic random-effects meta-analysis for the finally included in vitro and in vivo studies for all diagnostic methods at different caries detection levels

Method and parameters		Caries detection level		Dentin detection level		1/3 dentin detection level
		In vitro	In vivo	In vitro	In vivo	In vitro
Visual examination	*N* SE (95% CI) SP (95% CI) DOR (95% CI) AUC	9 0.64 (0.42–0.81) 0.85 (0.74–0.92) 11.49 (5.19–25.46) 0.84	-	1 0.09 (0.04–0.24) 0.99 (0.94–0.999) 13.26 (1.44–122.51) 0.85	2 0.32 (0.07–0.74) 0.76 (0.11–0.99) 1.84 (0.03–103.47) 0.53	1 0.93 (0.77–0.98) 0.84 (0.76–0.89) 67.0 (14.7–304.9) 0.95
Conventional bitewing radiography (D-speed)	*N* SE (95% CI) SP (95% CI) DOR (95% CI) AUC	2 0.19 (0.13–0.28) 0.96 (0.91–0.99) 5.2 (1.6–16.8) 0.55	-	1 0.17 (0.07–0.40) 0.99 (0.96–0.999) 31.0 (3.3–291.3) 0.91	-	-
Conventional bitewing radiography (E-speed)	*N* SE (95% CI) SP (95% CI) DOR (95% CI) AUC	9 0.42 (0.28–0.57) 0.90 (0.84–0.93) 6.80 (4.58–10.10) 0.82	-	5 0.67 (0.19–0.95) 0.94 (0.71–0.99) 31.40 (14.31–68.93) 0.92	1 0.35 (0.19–0.51) 0.91 (0.80–0.96) 4.8 (1.48–15.48) 0.74	-
Conventional bitewing radiography (F-speed)	*N* SE (95% CI) SP (95% CI) DOR (95% CI) AUC	8 0.43 (0.31–0.57) 0.88 (0.74–0.95) 5.34 (2.55–11.17) 0.72	-	3 0.42 (0.29–0.56) 0.92 (0.86–0.96) 8.7 (4.2–18.1) 0.81	-	1 0.54 (0.36–0.70) 0.996 (0.96–1) 283.6 (16.1–5010.0) 0.98
Digital bitewing radiography (sensor)	*N* SE (95% CI) SP (95% CI) DOR (95% CI) AUC	12 0.35 (0.26–0.45) 0.90 (0.85–0.93) 5.01 (2.83–8.88) 0.74	1 0.55 (0.42–0.67) 0.93 (0.77–0.98) 17.11 (3.73–78.39) 0.82	4 0.36 (0.30–0.42) 0.95 (0.93–0.97) 12.84 (6.56–25.13) 0.90	1 0.96 (0.91–0.98) 0.50 (0.02–0.98) 24.4 (0.44–1359.98) 0.89	-
Digital bitewing radiography (phosphor plate)	*N* SE (95% CI) SP (95% CI) DOR (95% CI) AUC	11 0.41 (0.25–0.61) 0.89 (0.83–0.93) 5.56 (3.04–10.16) 0.82	-	2 0.86 (0.03–0.99) 0.86 (0.11–0.99) 26.06 (7.29–93.21) 0.92	1 0.83 (0.77–0.88) 0.60 (0.20–0.90) 7.5 (1.19–47.13) 0.79	-
Laser fluorescence 2190	*N* SE (95% CI) SP (95% CI) DOR (95% CI) AUC	5 0.79 (0.62–0.90) 0.89 (0.76–0.95) 30.79 (8.74–108.51) 0.91	1 0.92 (0.82–0.96) 0.90 (0.73–0.97) 99 (22.01–445.30) 0.96	5 0.82 (0.58–0.94) 0.81 (0.78–0.85) 23.09 (7.01–76.04) 0.83	3 0.63 (0.58–0.68) 0.60 (0.15–0.93) 2.18 (0.24–19.95) 0.63	-
Fibre-optic transillumination FOTI	*N* SE (95% CI) SP (95% CI) DOR (95% CI) AUC	-	-	-	-	-

Data from the laboratory settings are based on the findings from a greater number of studies and, therefore, are more complete. The results from the bivariate diagnostic random-effects meta-analysis indicated that VE showed higher SE values for overall caries detection (0.64) and 1/3 dentin caries detection (0.93), while for dentin caries detection it was only 0.09. Contrary, SP for the dentine caries detection threshold was higher (0.99) than for overall caries detection (0.85) and 1/3 dentin caries detection thresholds (0.84). AUC values ranged from 0.84 to 0.95 for VE under in vitro conditions.

Among conv-BWR modalities, F-speed showed the highest SE (0.43) for the caries detection level and E-speed (0.67) for dentin caries detection. SP was high for both caries detection levels, ranging from 0.88 to 0.99 between the different modalities. The AUC values were lower for any type of caries detection level in comparison to the dentin caries detection level and ranged from 0.55 to 0.92. In general, the results for digital BWR were in the same order of magnitude, with exception of higher SE for phosphor plate-based BWR; the AUC values ranged between 0.74 and 0.92. The bivariate diagnostic random-effects meta-analysis showed a good diagnostic performance for LF on proximal sites in comparison to all other caries diagnostic methods irrespective of the cutoff level used; the documented AUC values ranged above 0.83. sROC plots and forest plots can be found in the supplemental online content.

## Discussion

In the case of proximal caries lesions, where direct VE is mostly impossible, the use of additional caries detection and diagnostic methods is typically indicated. During previous years, many systematic reviews and/or meta-analyses focusing on and analysing the diagnostic accuracy of these methods were undertaken (e.g. Refs. 13 and 17–23). In comparison to all previous work, the present systematic review and meta-analysis provide an overview and comparison between commonly used diagnostic test methods for proximal caries detection on the basis of the available literature from in vitro and in vivo caries diagnostic studies. Another unique feature of this work is that the spectrum of heterogeneity was narrowed due to the inclusion of a tailor-made RoB analysis, which resulted in the inclusion of studies with a low to moderate RoB.

When discussing the results from the systematic search of the literature, it is noteworthy that, first, the final number of selected studies was low and, second, that clinical trials (*N* = 5) were rare in comparison to laboratory studies (*N* = 31, Fig. 1, Tables 2 and 3). With respect to this imbalance, there seems to be an urgent need to design, plan, and conduct well-designed and highly standardised clinical diagnostic studies that compare different test methods in a well-justified and homogenous patient sample. Problematically, the clinical validation of the caries extent, cavity level, or activity by reference tests cannot be performed in full due to the unavailability of reference test methods for evaluating caries activity and the impossibility of applying histological methods under clinical conditions. This explains the documented imbalance, limits the planning of future clinical trials, and, considering the importance of clinical testing, also illustrates the need to develop clinically applicable reference standards, which may improve the present situation in the future.

Regarding the meta-analytic diagnostic performance of all the included diagnostic methods and used cutoff levels, it needs to be highlighted that, first, in some of the categories, only one and, at best, a few studies were identified (Tables 2 and 3). Second, several studies included only a small number of investigated teeth (Supplementary Tables S3g, h, S4g, h, S5g, h, S6g, and h). Third, the proportions of included teeth in relation to the caries spectrum were often misbalanced. Therefore, the results from this meta-analysis (Table 3) should not be overrated and generalised. Nevertheless, some aspects of the meta-analysis need to be discussed. Under in vitro conditions, all test methods showed mostly high SP values, while SE varied between the different methods and thresholds. A substantial difference between SE values was registered for VE under in vitro and in vivo conditions (Table 3), which was also reported by Gimenez et al. [18]. This finding is most likely related to the simple fact that clinical caries detection is more difficult to perform due to the limited direct view of proximal surfaces that could not be simulated in full under laboratory conditions. Here, VE under in vitro conditions probably provides more details, which results in higher SE values, with exception of results for dentin detection level based on just one study. This methodological aspect illustrates the difficulty of comparing data from clinical and in vitro investigations. Therefore, the results from any study need to be interpreted with consideration of the methodology of the corresponding trial.

For proximal caries detection and diagnostics, the BWR is the most frequently used additional method [14]. Therefore, it is not surprising that the majority of included studies investigated conventional and/or digital BWR. Thus, to eliminate possible bias originating from the use of different conventional film types, the available speed classes (D-, E-, and F-speed) were analysed separately. Similarly, studies on digital BWR that used sensor or phosphor plate imaging technology were also assessed separately, which is in contrast to a previously published systematic review that merged all these data into one category [13]. The results (Table 3) revealed high SP and low SE for all types of BWR except for phosphor plate-based systems. This ratio needs to be discussed, again, in relation to the included spectrum of caries lesions in the corresponding studies. Here, frequently, the proportion of dentin caries lesions was low. In contrast, when it was only possible to sample dentin caries lesions in a clinical investigation [67], the SE was mostly documented as good. This example highlights the influence of the sample constitution on diagnostic performance.

LF has been increasingly used as an additional caries detection aid [15] and has also been included in several diagnostic studies on proximal sites. The results found high AUC, SE, and SP values for LF, which is in line with earlier findings from Gimenez et al. [17]. Contrary to these encouraging results, clinical usage is sensitive, and good standardisation is essential to avoid false-positive readings due to other fluorescence sources [20].

This systematic review and meta-analysis have strengths and limitations from a methodological point of view. As for strengths, first, all diagnostic methods for proximal caries detection and diagnostics were merged into one meta-analysis. Second, the study selection followed a strict protocol and included only those studies with a low RoB in core categories. On the one hand, this procedure resulted in the selection of studies with a comparable methodology and good quality; on the other hand, it caused a substantial reduction in includable scientific reports. Another strength of this project seems to be the detailed and extensive documentation (Supplemental online content). As it is necessary to mention limitations, in many categories, no or only a few studies were available, which limits the generalisability of the meta-analytical results. Another potential limitation is the quality assessment of all studies that basically met the inclusion criteria. Here, extensive discussions were held in the study group regarding the question “Which indicators in the reporting are linked to which degree of bias?” It is possible that some of our decisions could be questioned, especially concerning studies with weak methodological reporting. Another limitation might be that variables, e.g. sample size, sample composition, sample storage, study setting, or examiner experience, which could possibly influence or confound the results of the meta-analysis remained unconsidered. This might be another reason not to overrate the findings from this meta-analysis.

## Conclusion

When considering the available data records and quality in relation to the consequences for future research, it must be concluded that there is an overall need for high-quality, well-designed, and well-powered caries detection and diagnostic studies. This need must be emphasised much more for clinical data. Another urgent void that has to be addressed is the non-availability of an acceptable reference standard for clinical caries detection and diagnostic studies. Here, experts should try to reach a consensus regarding which procedure will meet ethical and methodological requirements.

## Supplementary Information

Below is the link to the electronic supplementary material.

Supplementary file1 (PDF 5371 KB)
