# Supplementary material for: Systematic review and meta-analysis of diagnostic studies of proximal surface caries
Source: Clin Oral Investig. 2021 Sep 4;25(11):6069–79. doi: 10.1007/s00784-021-04113-1 (PMC8531083; doi:10.1007/s00784-021-04113-1)
Supplement: Supplementary file 1 — Supplementary file1 (PDF 5371 KB) [file 784_2021_4113_MOESM1_ESM.pdf]

Supplemental content

Content

Table S0 List of excluded studies with reasons for exclusion 5

Diagnostic test method criteria 6

Table S1a Visual examination criteria 6

Table S1b Radiography criteria 8

Table S1c Laser fluorescence criteria 10

Table S1d FOTI criteria 11

Table S1e Semiquantitative histological criteria 12

Risk of bias assessment 13

Table S2 Risk of bias assessment tool 13

Visual Examination 19

Table S3a Results of Systematic Literature Review for in vitro visual validation studies on proximal surfaces 19

Table S3b Results of Systematic Literature Review for in vivo visual validation studies on proximal surfaces 20

Table S3c Reproducibility/Reliability results of Systematic Literature Review for in vitro visual studies on proximal surfaces 21

Table S3d Reproducibility/Reliability results of Systematic Literature Review for in vivo visual studies on proximal surfaces 22

Table S3e Risk of bias assessment for visual examination of in vitro validation studies on proximal surfaces 23

Table S3f Risk of bias assessment for visual examination of in vivo validation studies on proximal surfaces 24

Figure S1 Risk of bias graph for in vivo and in vitro caries diagnostic studies with visual examination: review authors' judgements about each risk of bias item presented as percentages across all included studies 25

Table S3g Studies assessed for inclusion in the meta-analysis- visual examination of in vitro validation studies on proximal surfaces 26

Table S3h Studies assessed for inclusion in the meta-analysis- visual examination of in vivo validation studies on proximal surfaces 27

Conventional Radiography 28

Table S4a Results of Systematic Literature Review for in vitro conventional radiography validation studies on proximal surfaces 28

Table S4b Results of Systematic Literature Review for in vivo conventional radiography validation studies on proximal surfaces 31

Table S4c Reproducibility/Reliability results of Systematic Literature Review for in vitro conventional bitewing studies on proximal surfaces 32

Table S4d Reproducibility/Reliability results of Systematic Literature Review for in vivo conventional bitewing studies on proximal surfaces 34

Table S4e Risk of bias assessment for conventional bitewing radiography of in vitro validation studies on proximal surfaces 35

Table S4f Risk of bias assessment for conventional bitewing radiography of in vivo validation studies on proximal surfaces....... 39

Figure S2 Risk of bias graph for in vivo and in vitro caries diagnostic studies with conventional bitewing radiography: review authors' judgements about each risk of bias item presented as percentages across all included studies....... 40

Table S4g Studies assessed for inclusion in the meta-analysis- conventional bitewing radiography of in vitro validation studies on proximal surfaces 41

Table S4h Studies assessed for inclusion in the meta-analysis- visual examination of in vivo validation studies on proximal surfaces....... 43

Digital Radiography....... 44

Table S5a Results of Systematic Literature Review for in vitro digital radiography validation studies on proximal surfaces....... 44

Table S5b Results of Systematic Literature Review for in vivo digital radiography validation studies on proximal surfaces....... 48

Table S5c Reproducibility/Reliability results of Systematic Literature Review for in vitro digital bitewing studies on proximal surfaces 49

Table S5d Reproducibility/Reliability results of Systematic Literature Review for in vivo digital bitewing studies on proximal surfaces 52

Table S5e Risk of bias assessment for digital bitewing radiography of in vitro validation studies on proximal surfaces....... 53

Table S5f Risk of bias assessment for digital bitewing radiography of in vivo validation studies on proximal surfaces....... 57

Figure S3 Risk of bias graph for in vivo and in vitro caries diagnostic studies with digital bitewing radiography: review authors' judgements about each risk of bias item presented as percentages across all included studies 58

Table S5g Studies assessed for inclusion in the meta-analysis- digital bitewing radiography of in vitro validation studies on proximal surfaces 59

Table S5h Studies assessed for inclusion in the meta-analysis- digital bitewing radiography of in vivo validation studies on proximal surfaces 61

Laser Fluorescence....... 62

Table S6a Results of Systematic Literature Review for in vitro LF validation studies on proximal surfaces....... 62

Table S6b Results of Systematic Literature Review for in vivo Laser Fluorescence validation studies on proximal surfaces 63

Table S6c Reproducibility/Reliability results of Systematic Literature Review for in vitro laser fluorescence studies on proximal surfaces....... 64

Table S6d Reproducibility/Reliability results of Systematic Literature Review for in vivo laser fluorescence studies on proximal surfaces....... 65

Table S6e Risk of bias assessment for laser fluorescence measurements of in vitro validation studies on proximal surfaces....... 66

Table S6f Risk of bias assessment for laser fluorescence measurements of in vivo validation studies on proximal surfaces 67

Figure S4 Risk of bias graph for in vitro and in vivo caries diagnostic studies with laser fluorescence: review authors' judgements about each risk of bias item presented as percentages across all included studies 68

Table S6g Studies assessed for inclusion in the meta-analysis- laser fluorescence of in vitro validation studies on proximal surfaces....... 69

Table S6h Studies assessed for inclusion in the meta-analysis- laser fluorescence of in vivo validation studies on proximal surfaces 70

FOTI 71

Table S7a Results of Systematic Literature Review for in vitro FOTI validation studies on proximal surfaces 71

Table S7b Results of Systematic Literature Review for in vivo FOTI validation studies on proximal surfaces 71

Table S7c Reproducibility/Reliability results of Systematic Literature Review for in vitro FOTI studies on proximal surfaces 72

Table S7d Reproducibility/Reliability results of Systematic Literature Review for in vivo FOTI studies on proximal surfaces 72

Table S7e Risk of bias assessment for FOTI of in vitro validation studies on proximal surfaces 73

Table S7f Risk of bias assessment for FOTI of in vivo validation studies on proximal surfaces 74

Figure S5 Risk of bias graph for in vivo and in vitro caries diagnostic studies with FOTI: review authors' judgements about each risk of bias item presented as percentages across all included studies 75

Meta analytic statistics 76

Table S8 Forest plots (DOR) for different caries diagnostic methods- in vitro validation studies on proximal surfaces 76

Table S9 Forest plots (DOR) for different caries diagnostic methods- in vivo validation studies on proximal surfaces 79

Table S10 SROC for different caries diagnostic methods- in vitro validation studies on proximal surfaces 81

Table S11 SROC for different caries diagnostic methods- in vivo validation studies on proximal surfaces 85

Table S0 List of excluded studies with reasons for exclusion

Reason for exclusion	Study
**Induced caries lesion**	Okano et al. (1985), Eggertsson et al. (1999), Young and Featherstone (2005), Ferreira et al. (2006), Belem et al. (2013), Vieira et al. (2015), Kajan et al. (2015)
**Missing data/ Unclear data reporting**	Noar and Smith (1990), Pitts and Rimmer (1992), Choksi et al. (1994), Syriopoulos et al. (2000), Janhom et al. (2001), Hellen-Halme and Lith (2013), Nikneshan et al. (2015) Gray et al. (2017);
**No/ unsuitable reference standard**	Espelid and Tveit (1986), Obry-Musset et al. (1988), de Vries et al. (1990), Verdonschot et al. (1992), Svenson et al. (1993), Scarfe et al. (1994), Heaven et al. (1994), Ardakani et al. (2004), Otis and Sherman (2005), Akkaya et al. (2006), Aleksejuniene et al. (2006), Galcera Civera et al. (2007), Tsuchida et al. (2007), Akarslan et al. (2008), Crombie et al. (2009), Raghav et al. (2014), Yoon et al. (2017), Laitala et al. (2017), Berg et al. (2018)
**Occlusal surfaces**	Ketley and Holt (1993), Lussi and Hellwig (2006)
**Other language**	Li et al. (2006), Falahzadeh et al. (2013), Faghihian and Faghihian (2015),
**Out of subject**	Marsh et al. (1989), Verdonschot et al. (1991), De Araujo et al. (1992), Reddy and Sugandhan (1994), Vaarkamp et al. (1997a), Vaarkamp et al. (1997b),Tyndall et al. (1998),Syriopoulos et al. (1999), Caliskan Yanikoglu et al. (2000), Shi et al. (2001), Kidd et al. (2003),Haak et al. (2001),Koob et al. (2004), Llena-Puy and Forner (2005) Kielbassa et al. (2006), Matalon et al. (2007),Hellén-Halme et al. (2008), Valizadeh et al. (2009), Holtzman et al. (2011), Tracy et al. (2011), Qu et al. (2011), Schulze et al. (2011), Xavier et al. (2011), Maia et al. (2011), Akbari et al. (2013),Ritter et al. (2013), Madalli et al. (2014),Sansare et al. (2014a), Sansare et al. (2014b) ,Ozsevik et al. (2015), Dashpuntsag et al. (2017)
**Primary teeth**	Clifton et al. (1998), Hintze (2006)
**Review**	Vaarkamp et al. (2000)
**Secondary caries**	White et al. (1988)

Diagnostic test method criteria

Table S1a Visual examination criteria

Criteria No.	Authors	Crieria description
1	Bin-Shuwaish et al. (2008)	1.Halo present<br>2.Halo absent
2	Ekstrand et al. (2011)	*ICDAS-II Classification.*<br>0 = sound,<br>1 = first visual change in enamel (seen only after prolonged air drying or restricted to within the confines of a pit or fissure),<br>2 = distinct visual change in enamel,<br>3 = localized enamel breakdown (without clinical visua signs of dentinal involvement),<br>4 = underlying dark shadow from dentine, 5 = distinct cavity with visible dentine, 6 = extensive distinct cavity with visible dentine
3	Hintze et al. (1998)<br>Mialhe et al. (2003)	0 = sound<br>1 = non-cavitated caries<br>2 = cavitated caries
4	Senel et al. (2010)	0, no caries lesion;<br>1, opacity or cavitation in enamel;<br>2, cavitation in dentine;<br>3, cavitation in dentine extending to pulp.
5	Shimada et al. (2014)	0: Sound tooth surface. No evidence of caries or demineralization of enamel.<br>1: Superficial demineralization of enamel, but without cavitation. Opacity or discoloration (white or brown) is visible.<br>2: Localized enamel breakdown due to caries, with no visible dentin or underlying shadow. Carious lesion was limited to the depth of enamel.<br>3: Superficial dentin caries. Softened carious dentin was present, and the carious involvement was within the outer half of total thickness of dentin.<br>4: Deep dentin caries. Softened carious dentin was present, and deep dentin was involved, where caries infection extended into the inner half of total thickness of dentin.
6	Haak et al. (2002)	0 = no lesion,<br>1 = enamel opacity with smooth surface,<br>2 = enamel opacity with rough surface,<br>3 = cavitation restricted to the enamel,<br>4 = cavitation extending into dentine.
7	Hintze (2003)<br>De Araujo et al.(1992)	0 = sound<br>1 = caries lesion without cavitation,<br>2 = caries lesion with cavitation<br>3 = caries lesion with manifest cavitation

Criteria No.	Authors	Crieria description
8	Silva Neto et al. (2008)	0= sound<br>1= white spot<br>2= dark spot<br>3= white dark spot or cavitation
9	Bozdemir et al. (2016)	0= sound<br>1= presence of opaque white or brown spots<br>2= gray discoloration in the underlaying dentin
10	Peker et al. (2009)	0= sound<br>1= Enamel opacity with smooth surface<br>2= Enamel opacity with rough surface<br>3= Cavitation restricted to enamel<br>4= Cavitation extending into dentin

Table S1b Radiography criteria

Criteria No.	Authors	Healthy	Enamel caries		Dentine caries		
1	Rickets et al. (1997)<br>Dove et al. (1992)<br>Russel et al. (1993)<br>Pitts et al. (1984)<br>Marthaler et al. (1966)	0	1	2[#]	3	4	
2	Espelid et al.(1986)	0	1	2	3	4	5
3	Ekstrand et al. (2011)	0	1	2		3	4
4	Hintze et al. (1998)<br>Mialhe et al. (2003)	0	1	2	3	4	
5	Wenzel et al.(2007)	0	1		2	3	
6	Abesi et al. (2012)<br>Hintze (2003)	0	1		2	3	
7	Neuhaus et al. (2015)	0	1		2	3	4
8	da Silva Neto et al (2008)<br>Espellid et al. (1986)	0	1	2	3	4	5
9	Berkhout (2007)	0	1	2	3		
10	Behere et al. (2011)	0	1		2		
11	Peers et al. (2003)	1			2		
12	Wojtovicz et al. (2003)<br>Verdonschot et al. (1993)^	0	1	2[##]	3		
13	Haak et al (2003)	0	1	2	3	4	5
14	Wenzel et al. (2007a)	0	1		2	3	
15	Wenzel et al. (2013)	0-sound<br>1-lesion in enamel without cavitation<br>2- lesion in enamel with cavitation<br>3-lesion 1/3 or less in dentine without cavitation<br>4-lesion 1/3 or less in dentine with cavitation<br>5-lesion more than 1/3 in dentine without cavitation<br>6-lesion more than 1/3 in dentine with cavitation<br>7-surface not recordable					
16	Mehare et al. (1999),<br>Shimada et al. (2014)	0-sound<br>1-Superficial enamel demineralization<br>2-Localized enamel breakkdown<br>3-Superficial dentin caries<br>4-Deep dentin caries					
17	Wong et al. (2002)	1-less than halfaway through enamel<br>2-greater than halfaway through enamel<br>3-to the DEJ					

		4-beyond the DEJ<br>5-no lesion<br>6-unsure if a lesion is present
18	Hintze et al. (1994)<br>Wenzel et al. (1995)<br>Espelid et al. (1986)	*“5 point- confidence rating scale”*<br>score 1 = caries definitely absent;<br>score 2 = caries probably absent;<br>score 3 = unsure if present or absent;<br>score 4 = caries probably present<br>score 5 = caries definitely present.
19	Kay et al. (1992)	1- I would definitely restore this tooth surface<br>2- I would probably restore this tooth surface<br>3- I would possibly restore this tooth surface<br>4- I would possibly leave this tooth surface unrestored<br>5- I would probably leave this tooth surface unrestored<br>6- I would definitely leave this tooth surface unrestored
20	Castro et al. (2007)	Continuous confidence-rating scale from 1 to 100<br>with 1 representing “lesion definitely not present” and 100 representing “lesion definitely present.
21	Safi et al. (2015)	0: Definitely no caries<br>1: Enamel caries (radiolucency in enamel)<br>2: Dentine caries (radiolucency in dentine)<br>3: Deep dentine caries (radiolucency extending to pulp)
22	Booshehry et al. (2010)	0: No caries<br>1: Caries present

[#]+/- dentin-enamel junction (DEJ); [##]reaches and affects DEJ

Table S1c Laser fluorescence criteria

Criteria No.	Authors	Criteria description	
1	Kühnisch et al. (2016)<br>Huth et al. (2010)	Interproximal dentin caries was associated with values ≥16	
2	Neuhaus et al. (2015)	D0—(0–6)<br>D1—(6.1–13)<br>D2—(13.1–17)<br>D3, D4—(>17)	
3	Tagtekin et al. (2008)<br>Lussi et al. (2001)	(D1 sound fissures and enamel fissure lesions _0-13)<br>(D2 enamel caries _ 14–19) and<br>(D3 dentinal caries _ 20)	
4	Rodrigues. (2009)<br>Lussi et al. (2006)	Wedge shaped tip<br>D0=0-6<br>D1=6.1-9<br>D2=9.1-15<br>D3, D4>15	Tapered wedge shaped tip<br>D0= 0-9<br>D1= 9.1- 13<br>D2=13.1- 22<br>D3, D4> 22

Table S1d FOTI criteria

Criteria No.	Authors	Healthy	Enamel caries		Dentine caries	
1	Astvaldsdottir et al. (2012)	0	1	2	3	4
2	Hintze et al. (1998)	0	1		2	
3	Bin-Shuwaish et al. (2008)	They determined the extent of the lesion according to the size of the black area on the image and scored each lesion as<br>0, lesion not present;<br>1, small lesion present;<br>2, medium lesion present;<br>3, large lesion present.				
4	Peers et al. (1993)	1. Shadow or opacity beneath the marginal ridge up to DEJ<br>2. 1. Shadow or opacity beneath the marginal ridge beyound DEJ				
5	Abogazalah et al. (2019)	0. No caries present<br>1. Probably no caries present<br>2. Not sure if caries present<br>3. Probably present				

Table S1e Semiquantitative histological criteria

Criteria No.	Authors	Healthy	Enamel caries			Dentine caries		
1	Marthaler (1966)	0	1	2		3	4	
2	Lussi (1991)	0	1	2		3		
3	Nytun et al. (1992), Lazarchik et al. (1995), Gray und Paterson (1997), El-Housseiny und Jamjoum (2001), Costa et al. (2002), Fung et al. (2004), de Paula et al. (2009)	0	1			2		
4	Wenzel und Fejerskov (1992), Rodrigues et al. (2009)	0	1			2	3	
5	Ekstrand et al. (1997)	0	1	2		3	4	
6	Krzizostaniak et al. (2014)	0	1	2		3	4	
7	Cheng et al. (2012)	0	1	2	3*	4	5	
8	Espelid et al. (1986)	1	2	3		4	5	6
9	Khan et al. (2005) Pabla et al. (2003) Nair et al. (2001) Abreu et al. (2001) Abreu et al. (1999) Ludlow et al. (1999)	0	1	2**		3	4	5
10	Haak et al. (2003)	0	1	2		3	4	5
11	Haak et al. (2005)	0	1			2	3	4
12	Adibi et al. (2018)	0	1	2		3	4	5***
13	Isidor et al. (2009)	0	1			2	3	
14	Hellen-Halme et al. (2010)	0	1	2		3		
15	Booshehry et al. (2010)	0	1					
16	Peker et al. (2009)	0	1	2		3	4	
17	Moystad et al. (1996)	0	1	2	3	4		

* Caries reached but not crossed DEJ; ** +/-DEJ; *** caries reaching pulp

Risk of bias assessment

Table S2 Risk of bias assessment tool

<table>
<tr><th>Signalling questions</th><th>RoB</th><th colspan="5">Description of the criteria (Domain 1)</th></tr>
<tr><td rowspan="3">Patient selection bias
1. Is an eligible sample selected from the study participants/population?
In vivo studies only</td><td>Indicators for low RoB</td><td colspan="5">The eligible sample from the study participants/population is representative of the patients for whom the results of the study was applicable. The eligible sample is homogenous, enrolled consecutively or randomly, and obtained from the research question, e.g., PIRD.</td></tr>
<tr><td>Indicators for high RoB</td><td colspan="5">The eligible sample is not representative, the clinical indication for the application of the diagnostic test(s) is not completely described, and the sample is not enrolled consecutively or randomly; there is no research question.</td></tr>
<tr><td>Response options</td><td>Yes
(low RoB)</td><td>Most likely yes
(low RoB)</td><td>Most likely no
(high RoB)</td><td>No
(high RoB)</td><td>No information
(unclear)</td></tr>
<tr><td rowspan="3">Tooth selection bias
2. Is an eligible sample of teeth selected?</td><td>Indicators for low RoB</td><td colspan="5">Eligible selection of the target teeth and surfaces:
- In vitro/in vivo studies on occlusal caries detection = permanent molars/primary molars
- In vitro/in vivo studies on proximal caries detection = permanent molars & premolars/primary molars.</td></tr>
<tr><td>Indicators for high RoB</td><td colspan="5">The selected teeth and surfaces are not homogeneous; there is over/underrepresentation of at least one group of teeth, a mixture of posterior and anterior teeth, a mixture of permanent and primary teeth or a mixture of occlusal surfaces from premolars and molars.</td></tr>
<tr><td>Response options</td><td>Yes
(low RoB)</td><td>Most likely yes
(low RoB)</td><td>Most likely no
(high RoB)</td><td>No
(high RoB)</td><td>No information
(unclear)</td></tr>
</table>

Signalling questions	RoB	Description of the criteria (Domain 1)				
Spectrum bias 3. Is an appropriate spectrum of caries lesions selected?	Indicators for low RoB	All stages of caries (e.g., sound/enamel/dentin caries/caries at least in the inner half of the dentin or non-cavitated/cavitated caries) are included. The sampled caries spectrum should be pre-assessed.				
	Indicators for high RoB	At least one stage of caries is excluded or under/over-represented in the study.				
	Response options	Yes (low RoB)	Probably yes (low RoB)	Probably no (high RoB)	No (high RoB)	No information (unclear)
Sample size 4. Is the sample size appropriate for validity and reproducibility testing?	Indicators for low RoB	The sample size is statistically determined.				
	Indicators for high RoB	There is no sample size calculation, etc.				
	Response options	Yes (low RoB)	Probably yes (low RoB)	Probably no (high RoB)	No (high RoB)	No information (unclear)

Signalling questions	RoB	Description of the criteria (Domain 2)				
Index test criteria<br>5. Do/does the index test(s) correctly classify the target condition?	Indicators for low RoB	Exact pre-definition/prescription of the criteria used, thresholds for the index test. Correct usage of the index test(s) according to latest recommendations (justified on the basis of the references).				
	Indicators for high RoB	Modifications of the index test(s), mis-usage, misinterpretation.				
	Response options	Yes<br>(low RoB)	Probably yes<br>(low RoB)	Probably no<br>(high RoB)	No<br>(high RoB)	No information<br>(unclear)
Blinding bias (index test)<br>6. Are the index test(s) data interpreted without knowledge of the results of the reference standard?	Indicators for low RoB	Appropriate blinding of the examiners who are making the decisions/diagnoses from index test(s), e.g., at least a one-week interval between examinations, randomized/shuffled allocation of the order of specimen/images and/or inclusion of multiple examiners who are performing only one test each.				
	Indicators for high RoB	Insufficient blinding. Same examiner performed multiple tests.				
	Response options	Yes<br>(low RoB)	Probably yes<br>(low RoB)	Probably no<br>(high RoB)	No<br>(high RoB)	No information<br>(unclear)
Calibration bias (index test)<br>7. Were the examiners trained/calibrated for the performing the index test(s)?	Indicators for low RoB	Details and outcomes of the calibration training, including the Kappa values for intra- and inter-examiner reliability, are given. Calibration training must include an independent sample of individuals or teeth. Calibration data should not be interpreted/misunderstood as intra-examiner reliability.				
	Indicators for high RoB	Insufficient training/calibration.				
	Response options	Yes<br>(low RoB)	Probably yes<br>(low RoB)	Probably no<br>(high RoB)	No<br>(high RoB)	No information<br>(unclear)

Signalling questions	RoB	Description of the criteria (Domain 3)				
Reference test criteria<br>8. Does the reference test correctly classify the target condition?	Indicators for low RoB	Usage of an optimal ("perfect") reference standard, e.g., histology, microradiography or µCT. Exact pre-definition/prescription of the used criteria, thresholds for the reference test. Correct usage of the reference test according to the latest recommendations (justified on the basis of references). The reference test is conditionally independent of the index tests.				
	Indicators for high RoB	Usage of a sub-optimal ("imperfect") reference standard, e.g., radiography. Modifications of the reference test; mis-usage; misinterpretation. The reference test is conditionally not independent of the index test. Differential misclassification – the error rate is associated with the index test results. Non-differential misclassification – the error rate is independent of the index test results, but this can underestimate SE and SP.				
	Response options	Yes (low RoB)	Probably yes (low RoB)	Probably no (high RoB)	No (high RoB)	No information (unclear)
Blinding bias (reference test)<br>9. Is the reference test data interpreted without knowledge of the results of the index test(s)?	Indicators for low RoB	Appropriate blinding of the examiners who are making the decisions/diagnoses from reference test(s). For example, there is at least a one-week interval between examinations, randomized/shuffled allocation of the order of specimen/images and/or inclusion of multiple examiners, who are performing only one test each, with unawareness of the outcome of index test(s).				
	Indicators for high RoB	Insufficient blinding. For example, the same examiner performs multiple tests within a few days.				
	Response options	Yes (low RoB)	Probably yes (low RoB)	Probably no (high RoB)	No (high RoB)	No information (unclear)
Calibration bias (reference test)<br>10. Are the examiners trained/calibrated for performing the reference test(s)?	Indicators for low RoB	Details and outcomes of the calibration training, including Kappa values for intra- and inter-examiner reliability, are given. Calibration training must include an independent sample of individuals or teeth. Calibration data should not be interpreted/misunderstood as intra-examiner reliability.				
	Indicators for high RoB	Insufficient training/calibration.				
	Response options	Yes (low RoB)	Probably yes (low RoB)	Probably no (high RoB)	No (high RoB)	No information (unclear)
**Signalling questions**	**RoB**	**Description of the criteria (Domain 4)**				

Incorporation bias<br><br>11. Are the reference test(s) performed separately from the index test(s)?	Indicators for low RoB	The reference and index test are performed separately.				
	Indicators for high RoB	The index test is incorporated in a (composite) reference test; the result of the index test is explicitly used as a criterion for the reference test.				
	Response options	Yes (low RoB)	Probably yes (low RoB)	Probably no (high RoB)	No (high RoB)	No information (unclear)
Partial verification bias<br><br>12. Do all patients/teeth/surfaces undergo both the reference tests and the index tests?	Indicators for low RoB	Ensure that all patients/teeth/surfaces undergo both the reference tests and the index tests.				
	Indicators for high RoB	Identified when a non-random set of patients/teeth/surfaces does not undergo the reference test and the verification rate depends on the index test results.				
	Categories of RoB	Yes (low RoB)	Probably yes (low RoB)	Probably no (high RoB)	No (high RoB)	No information (unclear)
Differential verification bias<br><br>13. Do all patients/teeth/surfaces receive the same reference standard?	Indicators for low RoB	Ensure that all patients/teeth/surfaces receive the same reference standard.				
	Indicators for high RoB	Identified when a non-random set of patients/teeth/surfaces is verified with a second or third reference test, especially when this selection depends on the index test result.				
	Response options	Yes (low RoB)	Probably yes (low RoB)	Probably no (high RoB)	No (high RoB)	No information (unclear)

Signalling questions	RoB	Description of the criteria (Domain 4)				
Bias in the analysis 14. Are all patient/teeth/surfaces, uninterpretable or intermediate test results and withdrawals included in the analysis?	Indicators for low RoB	All patients (teeth) who entered the study are accounted for, and all uninterpretable or intermediate test results and withdrawals (including lost specimens of the teeth) are explained.				
	Indicators for high RoB	Not all patients (teeth) who entered the study are accounted for, and not all uninterpretable or intermediate test results and withdrawals (including lost specimens of the teeth) are explained.				
	Response options	Yes (low RoB)	Probably yes (low RoB)	Probably no (high RoB)	No (high RoB)	No information (unclear)
Validity bias 15. Are the validation of results for the test method(s) included in the analysis?	Indicators for low RoB	Full presentation of results: Cross-tabulation (or distribution) of the index and reference test results by the reference standard results. Estimates of diagnostic accuracy and their precision are included (SE, SP, Az value).				
	Indicators for high RoB	Insufficient/incomplete information's, e.g., missing 2x2 contingency tables and/or SE, SP, Az values. Incorrect statistics. Biased interpretation.				
	Response options*	Yes (low RoB)	Probably yes (low RoB)	Probably no (high RoB)	No (high RoB)	No information (unclear)
Reproducibility bias 16. Are the reliability data of results for the test method(s) included in analysis?	Indicators for low RoB	Full presentation of results: Intra- and inter-examiner reliability for all examiners and for all teeth. Correct statistical procedures, e.g., Kappa values, Bland-Altman-Plots, etc. Data from the calibration training have not been mis/interpreted as reliability.				
	Indicators for high RoB	Insufficient/incomplete information on intra- and inter-examiner reliability or incorrect statistics. Data for calibration purposes only. Biased interpretation.				
	Response options	Yes (low RoB)	Probably yes (low RoB)	Probably no (high RoB)	No (high RoB)	No information (unclear)

Visual Examination

Table S3a Results of Systematic Literature Review for in vitro visual validation studies on proximal surfaces

Visual Examination	Study material		Diagnostics	Histology				Validity					
In vitro validation studies on proximal surfaces	Teeth (N)	Molars /PM (N)	Visual criteria[1]	Hard tissue processing	Caries staining	Visualisation /magnification	Scoring criteria[2]	Caries detection level			Dentin caries detection level		
								SE (%)	SP (%)	Az (ROC)	SE (%)	SP (%)	Az (ROC)
Peers et al. (1993)	240	-/-	nr	Slices	-	Microscopy	nr	-	-	-	38	99	-
Haak et al. (2002)	160	-/-	6	Slices	-	Microscopy	nr	71	75	0.76	-	-	-
Hintze et al. (2003)	373^{surfaces}	304/57	7	Slices	-	Microscopy	2	-	-	-	-	-	-
Silva Neto et al. (2008)	44	22/22	8	Slices/Groun	-	Microscopy	9	65.6	83.3	-	-	-	-
Peker et al. (2009)	48	-/-	10	Slices	-	Microscopy	16	-	-	0.65/0.53	-	-	-
Mitropoulos et al. (2010)	20	8/12	2	Slices	-	Microscopy	2	96/92	50/63	0.73/0.77	100/94	41/50	0.71-0.68
Senel et al. (2010)	138	-/-	4	Slices	-	Scanner	4	-	-	0.63-0.67	-	-	-
Ekstrand et al. (2011)	140*,**	-/-	2	Slices	-	Microscopy	5	-	-	-	93#	84#	-
Ko et al. (2015)	95	-/-	2	Slices	-	Microscopy	1	80	68	0.74	64	68	0.66
Neuhaus et al. (2015)	118	118/-	2	Ground	-	Microscopy/Photo	4	33	84	-	4	89	-
Bozdemir et al. (2016)	156 ^{surfaces}	-/-	9	Slices	-	Microscopy	3	32.5/31.3	94.5/95.9	0.75/0.78	15.4/2.7	99.1/99.1	0.84/0.84
Jan et al. (2016)	50*	-/-	2	Slices	-	Microscopy	1	73	65	0.68	-	-	-
Abogazalah et al. (2019)	30^{surfaces}	-/-	2	-	-	M3cro-CT	6	89	30^{surfaces}83	0.90	-	-/- -	-
Tonkaboni et al. (2019)	108^{surfaces}	-/-	2	Slices	-	Microscopy	1	49	99	-	-	-	-

[1]See Table S1a; [2]See Table S1e; nr-not reported; *canines, ** front teeth, #threshold:outer 1/3 dentin

Table S3b Results of Systematic Literature Review for in vivo visual validation studies on proximal surfaces

Visual Examination	Study material			Diagnostics	In vivo validation methodology			Validity					
In vivo validation studies on proximal surfaces	Patients (N)	Age (years)	Teeth (N)	Visual Criteria	Histology technique	Validation	Reference method	Caries detection level			Dentin caries detection level		
								SE (%)	SP (%)	Az (ROC)	SE (%)	SP (%)	Az (ROC)
Hintze et al. (1998)	53	20-38	338 surfaces	3	nr	Tooth separation	3	-	-	-	34	98	-
Mialhe et al. (2003)	70	13-15	199 surfaces	3	nr	Tooth separation	3	-	-	-	-	-	-
Bin-Shuwaish et al. (2008)	21	20-54	51	1	nr	Radiography	nr	-	-	-	100	27	-
Kühnisch et al. (2016)	85	25	127 surfaces	2	Cav.Prep.	Visual/Radiography		-	-	-	16	-	0.68
Ozkan et al. (2017)	157	12-18	161 surfaces	2	Cav.Prep.	Visual/Radiography	4	-	-	-	54	100	0.77

nr-not reported

Table S3c Reproducibility/Reliability results of Systematic Literature Review for in vitro visual studies on proximal surfaces

Visual Examination	Study material			Diagnostics	Reproducibility/Reliability detection & diagnostic methods		
In vitro validation studies on proximal surfaces	Examiners (N)	Teeth (N)	Molars/PM (N)	Visual Criteria	Reproducibility/Reliability testing	Intraexaminer	Interexaminer
Peers et al. (1993)	1	240	-/-	nr	Cohen's Kappa	0.76	-
Haak et al. (2002)	14	160	-/-	6	Cohen's Kappa	0.41-0.46	-
Hintze et al. (2003)	2	373[surfaces]	-/-	7	Cohen's Kappa	0.78	-
Silva Neto et al. (2008)	3	44	22/22	8	Cohen's Kappa	-	0.64
Peker et al. (2009)	3	48	-/-	10	nr	-	-
Mitropoulos et al. (2010)	2	20	-/-	2	Cohen's Kappa	-	0.51
Senel et al. (2010)	3	138	-/-	4	Cohen's Kappa	0.74-0.91	-
Ekstrand et al. (2011)	3	140*	-/-	2	Cohen's Kappa	0.87-0.94	0.85-0.87
Ko et al. (2015)	1	95	-/-	2	ICC	0.96	-
Neuhaus et al. (2015)	2	118	118/-	2	Cohen's Kappa	0.40-0.43	0.64
Bozdemir et al. (2016)	2	156 [surfaces]	-/-	9	Cohen's Kappa	-	0.55
Jan et al. (2016)	2	50*	-/-	2	nr	-	-
Abogazalah et al. (2019)	3	30[surfaces]	-/-	2	ICC	0.79	0.72
Tonkaboni et al. (2019)	1	108[surf]	-/-	2	nr	-	-

nr-not reported; *canines

Table S3d Reproducibility/Reliability results of Systematic Literature Review for in vivo visual studies on proximal surfaces

Visual Examination	Study material				Diagnostics	Reproducibility/Reliability detection & diagnostic methods		
In vivo validation studies on proximal surfaces	Patients (N)	Examiners (N)	Teeth (N)	Molars/PM (N)	Visual Criteria	Reproducibility/Reliability testing	Intraexaminer	Interexaminer
Hintze et al. (1998)	53	4	338 surfaces	163/176	3	Cohen's Kappa	-	0.40-0.50
Mialhe et al. (2003)	70	3	199 surfaces	-/-	3	Cohen's Kappa	0.79	-
Bin-Shuwaish et al. (2008)	21	1	51	-/-	1	nr	-	-
Kühnisch et al. (2016)	85	2	127 surfaces	46/81	2	nr	-	-
Ozkan et al. (2017)	157	2	161 surfaces	-/-	2	Cohen's Kappa	0.76/0.63	0.41

Table S3e Risk of bias assessment for visual examination of in vitro validation studies on proximal surfaces

Visual examination of *in vitro* validation studies on proximal surfaces	Signaling questions															
	Selection bias				Index test bias			Reference test bias				Verification bias			Outcome bias	
	Patient selection	Teeth selection	Caries Spectrum	Sample size	Test Criteria	Blinding bias	Calibration bias	Test Criteria	Blinding bias	Calibration bias	Incorporation bias	Partial ver. bias	Differential ver. bias	Bias in the Analysis	Validity bias	Reproducibility bias
Peers et al. (1993)	x	?	?	-	?	+	-	?	+	?	+	?	-	-	?	?
Haak et al. (2002)	x	+	+	-	+	+	-	-	?	-	+	+	+	+	+	?
Hintze et al. (2003)	x	?	-	-	+	+	-	+	+	-	+	+	+	+	-	?
Silva Neto et al. (2008)	x	+	?	-	+	-	-	+	-	-	+	+	+	+	+	?
Peker et al. (2009)	x	?	+	-	+	+	-	+	-	+	+	+	+	+	?	-
Mitropoulos et al. (2010)	x	+	-	-	+	+	?	+	?	?	+	+	+	+	+	?
Senel et al. (2010)	x	?	?	-	+	?	-	+	?	-	+	+	+	+	+	+
Ekstrand et al. (2011)	x	-	+	-	+	+	+	+	+	+	+	+	+	+	+	+
Ko et al. (2015)	x	?	-	-	+	-	?	+	-	-	+	-	-	-	?	?
Neuhaus et al. (2015)	x	?	?	-	+	+	?	+	+	-	+	+	+	+	?	+
Bozdemir et al. (2016)	x	-	?	-	+	-	-	+	+	-	+	+	+	+	+	?
Jan et al. (2016)	x	+	+	+	+	+	+	+	+	?	+	+	+	+	+	-
Abogazalah et al. (2019)	x	-	-	+	+	+	+	+	+	+	+	+	+	+	+	+
Tonkaboni et al. (2019)	x	?	-	-	+	?	-	+	+	?	+	+	+	+	?	-

Legend: +=Low risk of bias (Yes); -=High risk of bias (Probably No, No); ?=Unclear (No information, Incomplete reporting, Probably Yes) x=Question for in vivo studies

Table S3f Risk of bias assessment for visual examination of in vivo validation studies on proximal surfaces

Visual examination of *in vivo* validation studies on proximal surfaces	Signaling questions															
	Selection bias				Index test bias			Reference test bias				Verification bias			Outcome bias	
	Patient selection	Teeth selection	Caries Spectrum	Sample size	Test Criteria	Blinding bias	Calibration bias	Test Criteria	Blinding bias	Calibration bias	Incorporation bias	Partial ver. bias	Differential ver. bias	Bias in the Analysis	Validity bias	Reproducibility bias
Hintze et al. (1998)	-	+	-	-	+	-	?	-	-	?	-	+	+	+	?	?
Mialhe et al. (2003)	-	?	-	-	+	+	?	+	-	?	+	+	+	+	?	-
Bin-Shuwaish et al. (2008)	-	?	-	-	?	+	-	+	+	-	+	+	+	+	?	-
Kühnisch et al. (2016)	-	+	-	+	+	+	+	+	+	+	+	+	+	+	+	-
Ozkan et al. (2017)	-	?	-	+	+	-	+	+	-	-	+	+	+	+	+	+

Legend: +=Low risk of bias (Yes); -=High risk of bias (No)? =Unclear (No information, Incomplete reporting, Probably Yes, Probably No)

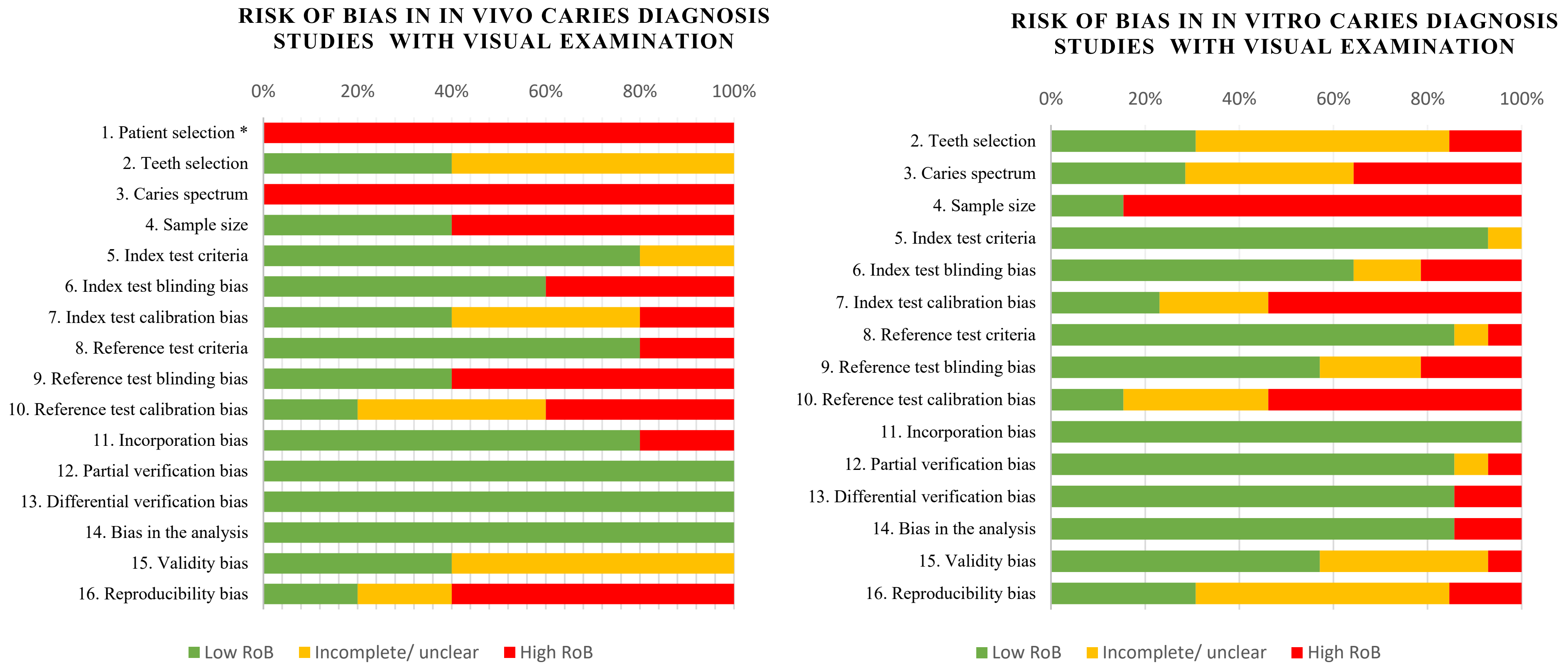

Figure S1 Risk of bias graph for in vivo and in vitro caries diagnostic studies with visual examination: review authors' judgements about each risk of bias item presented as percentages across all included studies

* Item no 1 (Patient selection bias) is only available for clinical diagnostic studies

Table S3g Studies assessed for inclusion in the meta-analysis- visual examination of in vitro validation studies on proximal surfaces

Studies selected in the 1st round	ITC	RTC	Sample size and caries distribution				Cut-off	Status in the 2nd round of selection	Reason for exclusion in the 2nd round	Cross-tabulation given	SE	SP	Az	Status in the 3th round of selection
			Total	S	E	D								
Hintze et al. 2003	7	2	373 surf.	208	113	52	Caries detection level	included		yes	-	-	-	included
Silva Neto et al. 2008	8	9	88 surf	24	46	**18**	Caries detection level	included		yes	65.6	83.3	-	included
Mitropoulos et al. 2010	2	2	40 surf.	16	6	18	Caries detection level	included		yes	96/92	50/63	0.73/0.77	included
							Dentin detection level	excluded	ITC not fitting Cut-off					excluded
Senel et al. 2010	4	4	276 surf.	142	40	94	Caries detection level	included		no	24*	92*	0.63-0.67	included
Ekstrand et al. 2011	2	5	151surf	33	52	28/38 (outer 1/3 dentin)	Ekstrand outer 1/3 dentin	included		yes	93	84	-	included
Neuhaus et al. 2014	2	4	118surf	*38*	*45*	*35*	Caries detection level	included		yes	33	84	-	included
							Dentin detection level	excluded	ITC not fitting Cut-off					excluded
Bozdemir et al. (2016)	9	3	156surf	73	44	39	Caries detection level	included		no	32.5/ 31.3	94.5/ 95.9	0.75/0.78	included
							Dentin detection level	included		no	15.4/ 2.7	99.1/ 99.1	0.84/0.84	included
Jan et al. 2016	2	1	100surf	40	47	13	Caries detection level	included		yes	73	65	0.68	included
Tonkaboni et al. 2019	2	1	108surf	245	13	66	Caries detection level	included		yes	49	99	-	included
Abogazalah et al. 2018	2	6	30surf	12	12	6	Caries detection level	included		no	89	83	0.90	included

ITC-Index test criteria, RTC- Reference test criteria, S-Sound, E-Caries in enamel, D- Caries in dentin, *calculated by us

Table S3h Studies assessed for inclusion in the meta-analysis- visual examination of in vivo validation studies on proximal surfaces

Studies selected in the 1st round	ITC	RTC	Sample size and caries distribution				Cut-off	2nd round of selection	Cross-tabulation given	SE	SP	Az	3th round of selection
			Total	S	E	D							
Kühnisch et al. 2016	2	*	127[surf]	0	0	127	Dentin detection level	included	yes	16	-	0.68	included
Ozkan et al. (2017)	2	4	161 [surf]	0	5	156	Dentin detection level	included	no	54	100	0.77	included

ITC-Index test criteria, RTC- Reference test criteria, S-Sound, E-Caries in enamel, D- Caries in dentin

Conventional Radiography

Table S4a Results of Systematic Literature Review for in vitro conventional radiography validation studies on proximal surfaces

Conventional bitewing radiography examination	Study material		Diagnostics	Validation methodology				Validity					
								Caries detection level			Dentin caries detection level		
In vitro validation studies on proximal surfaces	Teeth (N)	Molars/ PM (N)	Radiographic Criteria[1]	Hard tissue processing	Caries Staining	Visualisation /magnification	Scoring criteria[2]	SE (%)	SP (%)	Az (ROC)	SE (%)	SP (%)	Az (ROC)
Mileman et al. (1990) [D-speed]	105[surf]	-/-	18	Hemi/Slices	-	Micro radiography	nr	-	-	-	54	97	0.88
Kay et al. (1992) [unclear]	341[surf]	-/-	19	Slices	-	Microscopy	1	-	-	-	26	96	-
Dove et al. (1992) [D-speed]	80	40/40	1	Slices	-	Microscopy	1	-	-	0.75	-	-	-
Russel et al. (1993) [E- speed]	120	-/-	1	Hemi/Slices	-	Microscopy	1	25	90	-	30	96	-
Russel et al. (1993) [D-speed]	120	-/-	1	Hemi/Slices	-	Microscopy	1	25	90	-	29	92	-
Peers et al. (1993) [D-speed]	240	-/-	11	Slices	-	Microscopy	nr	-	-	-	59	96	-
Hintze et al. (1994) [D-speed]	66	-/-	18	Slices	-	Microscopy	1	-	-	0.61	-	-	-
Hintze et al. (1994) [E-speed]	66	-/-	18	Slices	-	Microscopy	1	-	-	0.61	-	-	-
Hintze et al. (1996a) [D-speed]	116	-/-	18	Slices	-	Microscopy	3	-	-	0.56/0.69	-	-	-
Hintze et al. (1996a) [E-speed]	116	-/-	18	Slices	-	Microscopy	3	-	-	0.55	-	-	-
Hintze et al. (1996b) [E-speed]	130	-/-	18	Slices	-	Microscopy	1	-	-	0.61-0.88	-	-	-
Downer et al. (1996) [D-speed]	344[surf]	-/-	19	Slices	-	Microscopy	1	-	-	-	0.5-52	92-99	-
Moystad et al. (1996) [E-speed]	50	25/25	18	Slices	-	Microscopy	17	-	-	0.69	-	-	0.79
Svanaes et al. (1996) [E-speed]	50	25/25	18	Hemisection	-	Microscopy	3	-	-	0.73	-	-	0.75
Ricketts et al. (1997) [D-speed]	180 [surf]	84/96	1	Slices	-	Microscopy	1	20-29	96-99	-	8-22	98-100	-
Ricketts et al. (1997) [E-speed]	180 [surf]	84/96	1	Slices	-	Microscopy	1	17-27	95-99	-	8-15	100	-
Ludlow et al. (1997) [D-speed]	64		18	Slices	-	Microscopy	9	-	-	-	-	-	-
Ludlow et al. (1997) [E-speed]	64		18	Slices	-	Microscopy	9	-	-	-	-	-	-
Ludlow et al. (1997) [F-speed]	64		18	Slices	-	Microscopy	9	-	-	-	-	-	-
Schneiderman et al. (1997) [E-speed]	50 [*,**]	14/12	nr	Slices/Groun	SvB[s]/Fuchsin	Microscopy	nr	21	91	-	-	-	-
White et al. (1997) [E-speed]	320[surf] [*,**]	80/80	18	Slices	-	Microscopy	1	43.2	88.8	-	49.6	97.1	-
Ariji et al. (1998) [E-speed]	118	-/-	1	Slices	-	Microscopy	1	30	88	-	34	98	-
Abreu et al. (1999) [E-speed]	40	20/20	18	Slices	-	Microscopy	9	-	-	-	-	-	-
Jessee et al. (1999) [D-speed]	26	-/-	1	Slices	-	Microscopy/Photo	1	-	-	-	-	-	-
Jessee et al. (1999) [E-speed]								-	-	-	-	-	-
Ludlow et al. (1999) [E-speed]	117[urf]	-/-	18	Slices	-	Microscopy/Photo	9	-	-	-	-	-	0.75
Svanaes et al. (2000) [E-speed]	120	61/59	18	Slices	-	Microscopy	1	48	94	0.77	-	-	-
White et al. (2000) [E-speed]	80	-/-	18	Hemisection	-	Microscopy/Probing	1	52/54	75/78	0.67/0.68	61/63	91/92	0.79/0.81
Abreu et al. (2001) [E-speed]	40	20/20	18	Slices	-	Microscopy	1	-	-	0.85	-	-	-

Conventional bitewing radiography examination	Study material		Diagnostics	Validation methodology				Validity					
In vitro validation studies on proximal surfaces								Caries detection level			Dentin caries detection level		
	Teeth (N)	Molars/ PM (N)	Radiographic Criteria[1]	Hard tissue processing	Caries Staining	Visualisation /magnification	Scoring criteria[2]	SE (%)	SP (%)	Az (ROC)	SE (%)	SP (%)	Az (ROC)
Espelid et al. (2001) [D-speed]	24	2/22	18	Ground/Drill	-	Visually	3	-	-	-	-	-	0.82
Ludlow et al. (2001) [D-speed]	40	20/20	18	Slices	-	Microscopy	9	-	-	0.88	-	-	-
Ludlow et al. (2001) [E-speed]	40	20/20	18	Slices	-	Microscopy	9	-	-	0.85	-	-	-
Ludlow et al. (2001) [F-speed]	40	20/20	18	Slices	-	Microscopy	9	-	-	0.84	-	-	-
Nair et al. (2001) [E-speed]	46	-/-	18	Slices	-	Microscopy	9	71	86	0.76	80	89	-
Nair et al. (2001) [F-speed]	46	-/-	18	Slices	-	Microscopy	9	74	88	0.77	85	92	-
Hintze et al. (2002a) [E-speed]	190	159/31	18	Slices	-	Microscopy	4	-	-	0.69	-	-	-
Hintze et al. (2002b) [E-speed]	177	146/31	18	Slices	-	Microscopy	4	-	-	0.70	-	-	-
Hintze et al. (2002b) [F-speed]	177	146/31	18	Slices	-	Microscopy	4	-	-	0.67	-	-	-
Matsuda et al. (2002) [D-speed]	30	-/30	18	-	-	Micro-CT	nr	-	-	0.71	-	-	-
Mileman et al. (2002) [D-speed]	105[surf]	-/-	18	Slices	-	Micro-radiography	nr	-	-	-	54/67.2	3.1/8.3	0.81/0.81
Wong et al. (2002) [D-speed]	40	-/-	17	Slices	-	Microscopy	nr	-	-	-	-	-	-
Wong et al. (2002) [E-speed]	40	-/-	17	Slices	-	Microscopy	nr	-	-	-	-	-	-
Hintze et al. (2003) [E-speed]	373[surf]	304/57	6	Slices	-	Microscopy	2	-	-	-	-	-	-
Hintze et al. (2003) [E-speed]	60	60/-	6	Hemisection	-	Microscopy	2	-	-	-	-	-	-
Matalon et al. (2003) [E-speed]	36	-/-	1	Slices	-	Microscopy	3	-	-	-	90	92	0.93
Wojtowicz et al. (2003) [F-speed]	96	-/-	12	Slices	-	Microscopy	2	-	-	-	-	-	-
Khan et al. (2004) [F-speed]	45	17/28	18	Slices	-	Microscopy	9	-	-	0.73	-	-	-
De Araujo et al. (2005) [E-speed]	52	-/52	1	Slices	-	Microscopy	1	-	-	0.87-0.95	-	-	-
De Araujo et al. (2005) [F-speed]	52	-/52	1	Slices	-	Microscopy	1	-	-	0.76-0.95	-	-	-
Erten et al. (2005) [D-speed]	40	-/-	18	Slices	-	Microscopy	6	39	91	-	-	-	-
Erten et al. (2005) [E-speed]	40	-/-	18	Slices	-	Microscopy	6	48	88	-	-	-	-
Erten et al. (2005) [F-speed]	40	-/-	18	Slices	-	Microscopy	6	45	84	-	-	-	-
Gungor et al. (2005) [F-speed]	80	40/40	18	Hemisection	-	Microscopy	9	-	-	-	-	-	-
Gungor et al. (2005) [E-speed]	80	40/40	18	Hemisection	-	Microscopy	9	-	-	-	-	-	-
Khan et al. (2005) [E-speed]	40	20/20	18	Slices	-	Microscopy	9	-	-	0.85	-	-	-
Rocha et al. (2005) [unclear]	48	-/-	11	Slices	-	Microscopy	nr	65/71	93/100	-	-	-	-
Akdeniz et al. (2006) [F-speed]	30	16/14	nr	Slices	-	Microscopy	nr	-	-	-	-	-	-
Lussi et al. (2006) [F-speed]	75	75/-	1	Ground	-	Microscopy/Photo	1	68	67	-	45	89	-
Kalathingal et al. (2007)[unclear]	20	-/-	18	Slices	-	Microscopy	2	51	92	0.79	-	-	-
Alkurt et al. (2007)) [E-speed]	48	24/24	18	Hemisection	fuchsin	Microscopy	5	-	-	0.81/0.84	-	-	-
Alkurt et al. (2007)) [F-speed]	48	24/24	18	Hemisection	fuchsin	Microscopy	5	-	-	0.79/0.80	-	-	-
Castro et al. (2007) [E-speed]	174	-/-	nr	Slices	-	Microscopy	nr	-	-	0.77	-	-	-
Peker et al. (2007) [E-speed]	48	24/24	18	Hemisection	fuchsin	Microscopy	5	-	-	0.84	-	-	-
Wenzel et al. (2007b) [F-speed]	80	40/40	13	Slices	-	Microscopy	5	18	92	-	-	-	-
Silva Neto et al. (2008) [E-speed]	44	22/22	8	Slices/Groun	-	Microscopy	9	30	96	-	-	-	-

Conventional bitewing radiography examination	Study material		Diagnostics	Validation methodology				Validity					
In vitro validation studies on proximal surfaces								Caries detection level			Dentin caries detection level		
	Teeth (N)	Molars/ PM (N)	Radiographic Criteria[1]	Hard tissue processing	Caries Staining	Visualisation /magnification	Scoring criteria[2]	SE (%)	SP (%)	Az (ROC)	SE (%)	SP (%)	Az (ROC)
Forner-Navarro et al. (2008) D-speed	192	-/-	1	Abrasion	-	Photomacroscope	1	14/18	91/98	-	5/3	100/96	-
Haiter Neto et al. (2008) [F-speed]	80	40/40	18+9	Slices	-	Microscopy	3	18	92	-	-	-	-
Rockenbach et al. (2008) [F-speed]	75	51/24	12	Hemisection	-	Microscopy	4	55.6	67.9	0.62	-	-	-
Peker et al. (2009) [F-speed]	48	-/-	1	Slices	-	Microscopy	16	-	-	0.77/0.80	-	-	-
Mitropoulos et al. (2010) [F-speed]	20	8/12	9	Slices	-	Microscopy	2	45/50	100/93	0.73/0.72	61/61	100/90	0.74/0.74
Pontual et al. (2010) [F-speed]	80	40/40	9	Slices	-	Microscopy	2	16	92	-	-	-	-
Senel et al. (2010) [E-speed]	138	-/-	6	Slices	-	Scanner	4	-	-	0.70-0.76	-	-	-
Bottenberg et al. (2011)[D-speed]	116[surf]	-/-	2	Slices/Groun	-	Microscopy/Photo	1	82-93	11-21	0.52/0.54	-	-	-
Bottenberg et al. (2011)[F-speed]	116[surf]	-/-	2	Slices/Groun	-	Microscopy/Photo	1	85-92	2-22	0.50/0.53	-	-	-
Ekstrand et al. (2011) [F-speed]	140*	-/-	3	Slices	-	Microscopy	5	-	-	-	-	-	-
Kayipmaz et al. (2011) [E-speed]	72	27/45	18	Slices	-	Microscopy	1	-	-	0.78	-	-	-
Zhang et al. (2011) [E-speed]	78[surf]	-/-	18	Slices	-	Microscopy	2	-	-	0.54	-	-	-
Abesi et al. (2012) [E-speed]	66[surf]	-/-	6	Slices	-	Microscopy	4	55	100	-	-	-	-
Astvaldsdottr et al. (2012) [E-speed]	56	-/56	1	Slices	-	Microscopy/M-rad	1	13-60	56-100	0.56-0.65	27-53	91-100	0.65-0.92
Kamburoglu et al. (2012) [F-speed]	80	-/-	18	Slices	-	Microscopy	4	69-84	94-99	0.83-0.92	-	-	-
Minston et al. (2013) [E-speed]	46	-/-	18	Slices	-	Microscopy	3	-	-	0.52/0.56	-	-	0.74/0.78
Pontual et al. (2013) [F-speed]	160 [surf]	-/-	18	Slices	-	Microscopy	3	-	-	0.51-0.63	-	-	-
De Souza et al. (2014) [F-speed]	51**	-/-	1	Slices	Rhodamine B	Microscopy	1	57	96	0.77	51	96	0.83
Krzyzostaniak et al. (2014) [F-speed]	135	68/67	4	Slices	-	Microscopy	6	-	-	0.67	-	-	-
Zayet et al. (2014) [E-speed]	75	-/-	1	Slices	-	Microscopy	1	90/90	63.3/ 66.7	0.80/0.72	100/10 0	46.2/44.2	-
Neuhaus et al. (2015) [F-speed]	118	118/-	7	Ground	-	Microscopy/Photo	4	23	86	0.80	27	94	0.65
Safi et al. (2015) [E-speed]	42	-/-	21	Slices	-	Microscopy	4	31.8	95.4	-	-	-	-
Jan et al. (2016) [F-speed]	50*	-/-	6	Slices	-	Microscopy	1	27	88	0.57	-	-	-
Melo et al. (2015) [F-speed]	20	10/10	18	Slices	-	Microscopy	3	-	-	0.49-0.63	-	-	-
Dehghani et al. (2017) [E-speed]	50	16/34	18	Slices	-	Microscopy	1	16.6	79.2/ 82.9	0.71/ 0.68			
Tonkaboni et al. (2019) [F-speed]	108[surf]	-/-	1	Slices	-	Microscopy	1	53	100				

[1]See Table S1b; [2]See Table S1e; nr-not reported *canines; **incisors

Table S4b Results of Systematic Literature Review for in vivo conventional radiography validation studies on proximal surfaces

Conventional bitewing radiography examination: In vivo validation studies on proximal surfaces	Study material: Patients (N)	Age (years)	Teeth (N)	Diagnostics: Radiographic Criteria	In vivo validation methodology: Histology technique	Validation	Reference method	Validity: Caries detection level SE (%)	SP (%)	Az (ROC)	Dentin caries detection level SE (%)	SP (%)	Az (ROC)
Hintze et al. (1996b) E-speed	nr	nr	130	18	Histo prep	*in vivo*	1	-	-	0.69-0.7	-	-	-
Hintze et al. (1998) E-speed	53	20-38	338 surfaces	4	nr	Tooth separation	3	-	-	-	41	99.9	-
Mialhe et al. (2003) E-speed	70	13-15	199 surfaces	4	nr	Tooth separation	3	-	-	-	-	-	-
Shimada et al. (2014) E-speed	53	21-64	86 surfaces	16	Cav.Prep.	Visual/Radiograph	5	-	-	-	35	91	0.63

nr-not reported

Table S4c Reproducibility/Reliability results of Systematic Literature Review for in vitro conventional bitewing studies on proximal surfaces

Conventional bitewing radiography examination	Study material			Diagnostics	Reproducibility/Reliability detection & diagnostic methods		
In vitro validation studies on proximal surfaces	Examiners (N)	Teeth (N)	Molars/PM (N)	Radiographic Criteria	Reproducibility/Reliability testing	Intraexaminer	Interexaminer
Mileman et al. (1990)	276	105	-/-	18	Cohen's Kappa	-	0.53
Kay et al. (1992)	20	341 surfaces	-/-	19	nr	-	-
Dove et al. (1992)	8	80	40/40	1	nr	-	-
Russel et al. (1993)	3	120	-/-	1	nr	-	-
Peers et al. (1993)	1	240	-/-	11	Cohen's Kappa	0.79	-
Hintze et al. (1994)	3	66	-/-	18	nr	-	-
Hintze et al. (1996a)	3	116	-/-	18	nr	-	-
Hintze et al. (1996a)	3	116	-/-	18	nr	-	-
Hintze et al. (1996b)	4	130	-/-	18	nr	-	-
Downer et al. (1996)	8	344 surf	-/-	19	nr	-	-
Moystad et al. (1996)	10	50	25/25	18	nr	-	-
Svanaes et al. (1996)	10	50	25/25	18	nr	-	-
Ricketts et al. (1997)	5	180 surf	84/96	1	ANOVA	-	-
Ludlow et al. (1997)	6	64		18	nr	-	-
Schneiderman et al. (1997)	5	50	14/12	nr	nr	-	-
White et al. (1997)	16	160 surfaces	80/80	18	nr	-	-
Ariji et al. (1998)	6	118	-/-	1	nr	-	-
Abreu et al. (1999)	8	40	20/20	18	ICC	0.74	0.60
Jessee et al. (1999)	8	26	-/-	1	nr	-	-
Ludlow et al. (1999)	6	122 surf	-/-	18	nr	-	-
Svanaes et al. (2000)	9	120	61/59	18	nr	-	-
White et al. (2000)	12	80	-/-	18	nr	-	-
Abreu et al. (2001)	6	40	20/20	18	nr	-	-
Espelid et al. (2001)	240	24	2/22	18	Cohen's Kappa	-	0.74
Ludlow et al. (2001)	6	40	20/20	18	ANOVA	-	-
Nair et al. (2001)	8	46	-/-	18	Cohen's Kappa	0.66	0.42
Hintze et al. (2002a)	4	190	159/31	18	nr	-	-
Hintze et al. (2002b)	4	177	143/31	18	nr	-	-
Matsuda et al. (2002)	3	30	-/30	18	nr	-	-
Mileman et al. (2002)	259	105 surfaces	-/-	18	nr	-	-
Wong et al. (2002)	6	40	-/-	17	nr	-	-
Hintze et al. (2003)	2	220 surfaces	-/-	6	Cohen's Kappa	0.76	-
Hintze et al. (2003)	2	26 surfaces	-/-	6	Cohen's Kappa	0.94	-
Matalon et al. (2003)	7	36	-/-	1	nr	-	-
Wojtowicz et al. (2003)	94	96	-/-	12	nr	-	-
Khan et al. (2004)	9	45	17/28	18	nr	-	-
De Araujo et al. (2005)	4	52	-/52	1	nr	-	-

Conventional bitewing radiography examination	Study material			Diagnostics	Reproducibility/Reliability detection & diagnostic methods		
In vitro validation studies on proximal surfaces	Examiners (N)	Teeth (N)	Molars/PM (N)	Radiographic Criteria	Reproducibility/Reliability testing	Intraexaminer	Interexaminer
Erten et al.(2005)	3	40	-/-	18	Spearman`s rho test	-	-
Gungor et al. (2005)	3	80	-/-	18	Gama Values	-	-
Khan et al. (2005)	4	40	20/20	18	nr	-	-
Rocha et al. (2005)	14	48	-/-	12	Cohen's Kappa	0.51-0.55	-
Akdeniz et al. (2006)	2	30	16/14	nr	Bland Altman	-	-
Lussi et al. (2006)	5	75	75.-	1	Cohen's Kappa	-	-
Kalathingal et al. (2007)	8	20	-/-	18	Cohen's Kappa	-	0.46
Alkurt et al. (2007)	2	48	24/24	18	ANOVA	-	-
Castro et al. (2007)	7	174	-/-	nr	Pearson corr. coeff./ Kendall	-0.02	0.43
Peker et al. (2007)	2	48	24/24	18	nr	-	-
Wenzel et al. (2007b)	6	80	40/40	5	nr	-	-
Silva Neto et al. (2008)	3	44	22/22	8	Cohen's Kappa	-	0.64
Forner-Navarro et al. (2008)	1	192	-/-	1	Cohen's Kappa	0.98	-
Haiter Neto et al. (2008)	6	80	40/40	18+9	nr	-	-
Rockenbach et al. (2008)	1	75	51/24	12	Kendall's test	0.85	-
Peker et al. (2009)	3	48	-/-	1	nr	-	-
Mitropoulos et al. (2010)	2	20	-/-	9	Cohen's Kappa	-	0.67
Pontual et al. (2010)	17	80	40/40	9	nr	-	-
Senel et al. (2010)	3	138	-/-	6	Cohen's Kappa	0.83-0.89	-
Bottenberg et al. (2011)	3	116[surfaces]	-/-	1	Cohen's Kappa	0.72-0.80	-
Ekstrand et al. (2011)	3	140*	-/-	3	Cohen's Kappa	0.62-0.81	0.56-0.60
Kayipmaz et al. (2011)	2	72	27/45	18	nr	-	-
Zhang et al. (2011)	7	78[surfaces]	-/-	18	Paired t test	-	-
Abesi et al. (2012)	4	66[surfaces]	-/-	6	nr	-	-
Astvaldsdottr et al. (2012)	8	56	-/56	1	Cohen's Kappa	0.82	0.63-0.78
Kamburoglu et al. (2012)	3	80	-/-	18	Cohen's Kappa	0.88-0.96	0.71-0.74
Minston et al. (2013)	20	46	-/-	18	nr	-	-
Pontual et al. (2013)	6	160 [surfaces]	-/-	18	nr	-	-
De Souza et al. (2014)	2	51**	-/-	1	Cohen's Kappa	0.68/0.90	0.69
Krzyzostaniak et al. (2014)	2	135	68/67	4	nr	-	-
Zayet et al. (2014)	6	75	-/-	1	Cohen's Kappa	0.76-0.79	0.75-0.78
Neuhaus et al. (2015)	2	120	120/-	7	Cohen's Kappa	0.68-0.73	0.66
Safi et al. (2015)	4	42	-/-	21	nr	-	-
Jan et al. (2016)	2	50*	-/-	6	nr	-	-
Melo et al. (2015) [F-speed]	3	20	10/10	18	Cohen's Kappa	-	-
Dehghani et al. (2017)	2	50	16/34	18	nr	-	-
Tonkaboni et al. (2019)	1	108[surf]	-/-	1	nr	-	-

nr-not reported; *canines; **incisors

Table S4d Reproducibility/Reliability results of Systematic Literature Review for in vivo conventional bitewing studies on proximal surfaces

Conventional bitewing radiography examination	Study material				Diagnostics	Reproducibility/Reliability detection & diagnostic methods		
In vivo validation studies on proximal surfaces	Patients (N)	Examiners (N)	Teeth (N)	Molars/PM (N)	Radiographic Criteria	Reproducibility/Reliability testing	Intraexaminer	Interexaminer
Hintze et al. (1996b)	nr	4	130	123/7	18	nr	-	-
Hintze et al. (1998)	53	4	338 surfaces	163/176	4	Cohen's Kappa	-	0.48-0.65
Mialhe et al. (2003)	70	3	199 surfaces	-/-	4	Cohen's Kappa	0.85	-
Shimada et al. (2014)	53	5	86 surfaces	-/-	16	nr	-	-

nr-not reported

Table S4e Risk of bias assessment for conventional bitewing radiography of in vitro validation studies on proximal surfaces

Conventional bitewing of *in vitro* validation studies on proximal surfaces	Signaling questions																
	Selection bias				Index test bias			Reference test bias				Verification bias			Outcome bias	
	Patient selection	Teeth selection	Caries Spectrum	Sample size	Test Criteria	Blinding bias	Calibration bias	Test Criteria	Blinding bias	Calibration bias	Incorporation bias	Partial ver. bias	Differential ver. bias	Bias in the Analysis	Validity bias	Reproducibility bias
Mileman et al. (1990)	x	?	?	-	-	+	-	+	+	-	+	+	+	+	?	?
Kay et al. (1992)	x	?	+	-	-	+	-	+	-	-	+	+	+	+	+	-
Dove et al. (1992)	x	+	+	-	-	+	-	+	-	-	+	+	+	+	?	-
Russel et al. (1993)	x	?	-	-	+	+	-	+	+	-	+	+	+	+	?	?
Peers et al. (1993)	x	?	?	-	?	+	-	?	+	?	+	?	-	-	?	?
Hintze et al. (1994)	x	?	-	-	-	+	-	+	-	-	+	+	+	+	-	-
Downer et al. (1996)	x	?	+	-	-	+	?	+	-	?	+	+	+	+	?	-
Hintze et al. (1996a)	x	?	-	-	-	+	-	+	-	-	+	+	+	+	?	-
Hintze et al. (1996b)	x	-	-	-	-	+	?	+	?	-	+	+	+	+	?	-
Moystad et al. (1996)	x	+	?	-	-	?	?	+	-	-	+	+	+	+	?	-
Svanaes et al. (1996)	x	+	?	-	-	+	-	+	-	-	+	+	+	+	?	-
Ricketts et al. (1997)	x	+	?	-	+	+	-	+	+	-	+	+	+	+	?	-
Ludlow et al. (1997)	x	+	-	-	-	+	-	+	?	-	+	+	+	-	-	-
Schneiderman et al. (1997)	x	-	-	-	-	-	?	-	-	-	-	-	+	+	-	-
White et al. (1997)	x	-	+	-	-	+	-	+	?	-	+	+	+	+	?	-
Ariji et al. (1998)	x	?	-	-	+	?	-	+	?	-	+	+	+	+	?	-
Abreu et al. (1999)	x	+	-	-	-	-	?	+	+	-	+	+	+	+	?	-
Jessee et al. (1999)	x	+	-	-	-	+	-	+	+	-	+	+	+	+	?	-
Ludlow et al. (1999)	x	+	?	-	-	+	-	+	?	-	+	+	+	+	?	-
Svanaes et al. (2000)	x	+	?	-	-	+	-	+	?	-	+	+	+	+	?	-

Conventional bitewing of *in vitro* validation studies on proximal surfaces	Signaling questions															
	Selection bias				Index test bias			Reference test bias				Verification bias			Outcome bias	
	Patient selection	Teeth selection	Caries Spectrum	Sample size	Test Criteria	Blinding bias	Calibration bias	Test Criteria	Blinding bias	Calibration bias	Incorporation bias	Partial ver. bias	Differential ver. bias	Bias in the Analysis	Validity bias	Reproducibility bias
White et al. (2000)	x	+	?	-	-	+	-	+	?	-	+	+	+	+	?	-
Abreu et al.(2001)	x	+	+	-	-	+	?	+	?	-	+	+	+	+	?	-
Espelid et al. (2001)	x	-	-	-	-	+	-	+	+	-	+	+	+	+	?	-
Ludlow et al. (2001)	x	+	+	-	-	+	?	+	?	-	+	+	+	+	?	-
Nair et al. (2001)	x	?	-	-	-	+	?	+	?	-	+	+	+	+	?	+
Hintze et al. (2002a)	x	+	-	-	-	+	-	+	+	?	+	+	+	+	?	-
Hintze et al. (2002b)	x	+	-	-	-	?	-	+	?	-	+	+	+	+	?	-
Matsuda et al. (2002)	x	-	-	-	-	+	-	-	-	-	+	+	+	+	?	-
Mileman et al. (2002)	x	?	-	-	-	+	-	-	+	-	+	+	+	+	+	-
Wong et al. (2002)	x	+	+	-	+	+	-	?	+	-	+	+	+	+	?	-
Hintze et al. (2003)	x	?	-	-	+	+	-	+	+	-	+	+	+	+	-	?
Matalon et al. (2003)	x	?	?	-	+	+	-	+	+	-	+	+	+	+	?	-
Wojtowicz et al. (2003)	x	?	+	-	+	+	-	-	+	-	+	+	+	+	-	-
Khan et al. (2004)	x	+	+	-	-	+	-	+	+	-	?	+	+	+	?	-
De Araujo et al. (2005)	x	-	+	-	+	-	+	+	-	-	+	+	+	+	?	-
Erten et al. (2005)	x	?	?	-	-	+	-	+	-	-	+	+	+	+	?	?
Gungor et al. (2005)	x	+	?	-	-	+	-	+	+	-	+	+	+	+	?	?
Khan et al. (2005)	x	+	+	-	-	+	?	+	+	-	+	+	+	+	?	-
Setti da Rocha et al. (2005)	x	-	-	-	-	+	-	-	-	-	?	+	+	+	?	?
Akdeniz et al. (2006)	x	+	?	-	-	-	-	-	-	-	+	+	+	+	-	-

Conventional bitewing of *in vitro* validation studies on proximal surfaces	Signaling questions															
	Selection bias				Index test bias			Reference test bias				Verification bias			Outcome bias	
	Patient selection	Teeth selection	Caries Spectrum	Sample size	Test Criteria	Blinding bias	Calibration bias	Test Criteria	Blinding bias	Calibration bias	Incorporation bias	Partial ver. bias	Differential ver. bias	Bias in the Analysis	Validity bias	Reproducibility bias
Kalathingal et al. (2007)	x	?	?	-	+	+	?	+	+	?	+	+	+	+	?	?
Lussi et al. (2006)	x	-	-	-	+	+	-	+	-	-	+	+	+	+	?	-
Alkurt et al. (2007)	x	+	-	-	-	+	-	+	-	-	+	+	+	+	?	?
Castro et al. (2007)	x	?	?	-	-	+	-	+	?	-	+	+	+	+	?	?
Peker et al. (2007)	x	+	?	-	-	-	-	+	-	-	+	+	+	+	?	-
Wenzel et al. (2007b)	x	+	-	-	+	+	-	+	+	-	+	+	+	+	?	-
Silva Neto et al. (2008)	x	+	?	-	+	-	-	+	-	-	+	+	+	+	+	?
Forner-Navarro et al. (2008)	x	+	-	+	+	-	-	+	-	-	+	+	+	+	?	-
Haiter Neto et al. (2008)	x	+	+	-	-	+	-	+	+	?	+	+	+	+	?	-
Rockenbach et al. (2008)	x	+	-	-	+	+	-	+	+	-	+	+	+	+	?	?
Peker et al. (2009)	x	?	+	-	+	+	-	+	-	+	+	+	+	+	?	-
Mitropoulos et al. (2010)	x	+	-	-	+	+	?	+	-	?	+	+	+	+	+	?
Pontual et al. (2010)	x	+	?	-	+	+	-	+	-	-	+	+	+	+	+	?
Senel et al. (2010)	x	?	?	-	+	?	-	+	?	-	+	+	+	+	+	+
Bottenberg et al. (2011)	x	?	?	-	+	?	-	+	+	-	+	+	+	+	-	-
Ekstrand et al. (2011)	x	-	+	-	+	+	+	+	+	+	+	+	+	+	+	+
Kayipmaz et al. (2011)	x	+	-	-	-	-	-	+	-	-	+	+	+	+	?	-
Zhang et al. (2011)	x	-	-	-	-	+	-	+	+	-	+	+	+	+	?	?
Abesi et al. (2012)	x	?	?	-	+	+	-	+	+	-	+	+	+	+	-	-
Astvaldsdottir et al. (2012)	x	?	?	-	+	+	-	+	+	?	+	+	+	+	+	+
Kamburoglu et al. (2012)	x	?	-	-	-	+	-	+	+	-	+	+	+	+	?	+

Conventional bitewing of *in vitro* validation studies on proximal surfaces	Signaling questions															
	Selection bias				Index test bias			Reference test bias				Verification bias			Outcome bias	
	Patient selection	Teeth selection	Caries Spectrum	Sample size	Test Criteria	Blinding bias	Calibration bias	Test Criteria	Blinding bias	Calibration bias	Incorporation bias	Partial ver. bias	Differential ver. bias	Bias in the Analysis	Validity bias	Reproducibility bias
Minston et al. (2013)	x	?	-	-	-	+	-	+	+	-	+	+	+	+	?	-
Pontual et al. (2013)	x	-	-	-	-	+	+	+	+	-	+	+	+	+	?	-
De Souza et al. (2014)	x	-	+	+	+	+	-	+	+	-	+	+	+	+	?	+
Krzyzostaniak et al. (2014)	x	+	-	-	+	+	-	+	+	-	+	+	+	+	?	-
Zayet et al. (2014)	x	?	?	-	+	+	-	+	-	-	+	+	+	+	+	+
Neuhaus et al. (2015)	x	?	?	-	+	+	?	+	+	-	+	+	+	+	?	+
Safi et al. (2015)	x	?	?	-	+	-	-	+	-	+	+	+	+	+	+	-
Jan et al. (2016)	x	+	+	+	+	+	-	+	+	?	+	+	+	+	+	-
Melo et al. (2015)	x	+	-	-	-	+	+	+	?	?	+	+	+	+	?	?
Dehghani et al. (2017)	x	+	?	-	-	+	-	+	+	-	+	+	+	+	+	-
Tonkaboni et al. (2019)	x	?	-	-	+	?	-	+	+	?	+	+	+	+	?	-

Legend: +=Low risk of bias (Yes); -=High risk of bias (Probably No, No);?=Unclear (No information, Incomplete reporting, Probably Yes) x=Question for in vivo studies

Table S4f Risk of bias assessment for conventional bitewing radiography of in vivo validation studies on proximal surfaces

Conventional bitewing of *in vivo* validation studies on proximal surfaces	Signaling questions															
	Selection bias				Index test bias			Reference test bias				Verification bias			Outcome bias	
	Patient selection	Teeth selection	Caries Spectrum	Sample size	Test Criteria	Blinding bias	Calibration bias	Test Criteria	Blinding bias	Calibration bias	Incorporation bias	Partial ver. bias	Differential ver. bias	Bias in the Analysis	Validity bias	Reproducibility bias
Hintze et al. (1996b)	-	-	-	-	-	-	-	+	?	-	+	+	+	+	?	-
Hintze et al. (1998)	-	+	-	-	+	-	?	-	-	?	-	+	+	+	?	?
Mialhe et al. (2003)	-	?	-	-	+	+	?	+	-	?	+	+	+	+	?	-
Shimada et al. (2014)	-	?	-	-	+	+	?	+	+	-	+	+	-	+	?	-

Legend: +=Low risk of bias (Yes); -=High risk of bias (Probably No, No); ?=Unclear (No information, Incomplete reporting, Probably Yes)

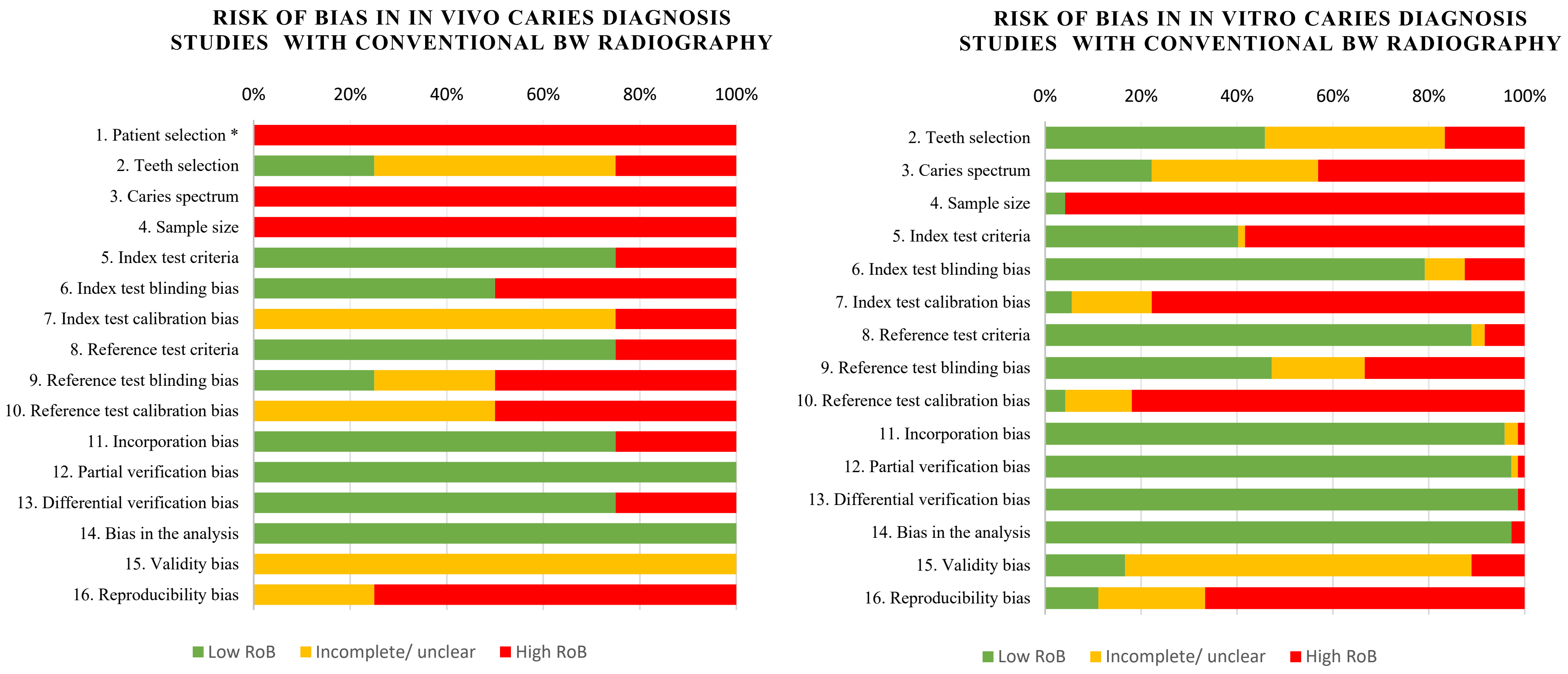

Figure S2 Risk of bias graph for in vivo and in vitro caries diagnostic studies with conventional bitewing radiography: review authors’ judgements about each risk of bias item presented as percentages across all included studies

* Item no 1 (Patient selection bias) is only available for clinical diagnostic studies

Table S4g Studies assessed for inclusion in the meta-analysis- conventional bitewing radiography of in vitro validation studies on proximal surfaces

Studies that passed 1st round of selection	ITC	RTC	Sample size and caries distribution				Radiogr. mode	Cut-off	2nd round of selection	Cross-tabulation given	SE	SP	Az	3th round of selection
			Total	S	E	D								
Russel et al. 1993	1	1	120^{teeth}				E-speed	Caries detection level	included	no	25	90	-	excluded
							E-speed	Dentin detection level	included	no	30	96	-	
							D-speed	Caries detection level	included	no	26	90	-	
							D-speed	Dentin detection level	included	no	29	92	-	
Ricketts et al. 1997	1	1	180^{surf}	111	45	24	E-speed	Caries detection level	included	no	17-27	95-99	-	included
							E-speed	Dentin detection level	included	no	8-15	100	-	
							D-speed	Caries detection level	included	no	20-29	96-99	-	
							D-speed	Dentin detection level	included	no	8-22	98-100	-	
Ariji et al. (1998)	1	1	236^{surf}	54	86	96	E-speed	Caries detection level	included	no	30	88	-	included
								Dentin detection level	included	no	34	98	-	
Matalon et al. 2003	1	3	36^{surf}	14	0	22	E-speed	Dentin detection level	included	no	90	92	0.93	included
Hintze et al. 2003	6	2	$373^{surf.}$	208	113	52	E-speed	Caries detection level	included	yes	-	-	-	included
Lussi et al. 2006	1	1	150^{surf}	61	52	37	F-speed	Caries detection level	included	no	68	67	-	included
								Dentin detection level	included	no	45	89	-	
Wenzel et al. 2007b	13	5	160^{surf}	101	50	9	F speed	Caries detection level	included	no	18	92	-	included
Silva Neto et al. 2008	8	9	88^{surf}	24	46	18	E-speed	Caries detection level	included	yes	30	96	-	included
Rockenbach et al. 2008	12	4	100^{surf}	28	49	23	F-speed	Caries detection level	included	no	55.6	67.9	0.62	included
Forner-Navarro et al. 2008	1	1	$384^{surf.}$	-	-	-	D-speed	Caries detection level	included	no	14/18	91/98	-	excluded
								Dentin detection level	included	no	5/3	100/96	-	
Pontual et al. 2010	9	2	$152^{surf.}$	88	64	-	F-speed	Caries detection level	included	no	16	92	-	excluded
Mitropoulos et al. 2010	9	2	$40^{surf.}$	16	6	18	F-speed	Caries detection level	included	yes	45/50	100/93	0.73/0.72	Included
								Dentin detection level	included	yes	61/61	100/90	0.74/0.74	excluded

Senel et al. 2010	6	4	276 surf.	142	40	94	E-speed	Caries detection level	included	no	-	-	0.70-0.76	included
Bottenberg et al. 2011	2	1	116 surf.	61	55	-	F-speed	Caries detection level	included	no	85-92	2-22	0.50-0.53	excluded
							D-speed	Caries detection level	included	no	82-93	11-21	0.52-0.54	
Astvaldsdottr et al. 2012	1	1	97surf.	34	48	15	E-speed	Caries detection level	included	no	13-60	56-100	0.56-0.65	included
								Dentin detection level	included	no	27-53	91-100	0.65-0.92	
Abesi et al. 2012	6	4	66 surf	46	13	7	E-speed	Caries detection level	included	no	55	100		included
De Souza et al. 2014	1	1	102 surf	53	14	35	F-speed	Caries detection level	included	no	57	96	0.77	included
								Dentin detection level	included	no	51	96	0.83	
Zayet et al. (2014)	1	1	150 surf	80	22	48	E-speed	Caries detection level	included	no	90/90	63.3/66.7	0.80/0.72	included
								Dentin detection level	included	no	100/100	46.2/44.2	-	
Neuhaus et al. 2015	7	4	118surf	*38*	*45*	*35*	F speed	Caries detection level	included	yes	23	86	0.80	included
								Dentin detection level	included	yes	27	94	0.65	
Safi et al. (2015)	21	4	84surf	54	11	19	E speed	Caries detection level	included	yes	31.8	95.4	-	included
Jan et al. 2016	6	1	100surf	40	47	13	F speed	Caries detection level	included	yes	27	88	0.57	included
Krzyostaniak et al. 2014	4	6	270surf	157	74	39	F-speed	Caries detection level	included	no	-	-	0.67	excluded
Ekstrand et al. 2011	3	5	151surf	33	52	28/38 (outer 1/3 dentin)	F-speed	Ekstrand outer 1/3 dentin	included	yes	54	100	-	included
Tonkaboni et al. 2019	1	1	108surf	245	13	66	F-speed	Caries detection level	included	yes	53	100	-	included

ITC-Index test criteria, RTC- Reference test criteria, S-Sound, E-Caries in enamel, D- Caries in dentin

Table S4h Studies assessed for inclusion in the meta-analysis- visual examination of in vivo validation studies on proximal surfaces

Studies selected in the 1st round	ITC	RTC	Sample size and caries distribution				Cut-off	2nd round of selection	Cross-tabulation given	SE	SP	Az	3th round of selection
			Total	S	E	D							
Shimada et al. et 2014	5	16	86 surf	28	25	33	Dentin detection level	included	no	35	91	0.63	included

ITC-Index test criteria, RTC- Reference test criteria, S-Sound, E-Caries in enamel, D- Caries in dentin

Digital Radiography

Table S5a Results of Systematic Literature Review for in vitro digital radiography validation studies on proximal surfaces

Digital bitewing radiography examination	Study material		Diagnostics	Validation methodology				Validity					
								Caries detection level			Dentin caries detection level		
In vitro validation studies on proximal surfaces	Teeth (N)	Molars/ PM (N)	Radiographic Criteria[1]	Hard tissue processing	Caries Staining	Visualisation /magnification	Scoring criteria[2]	SE (%)	SP (%)	Az (ROC)	SE (%)	SP (%)	Az (ROC)
Hintze et al. (1994) [sensor]	66	-/-	18	Slices	-	Microscopy	1	-	-	0.54	-	-	-
Wenzel et al. (1995)[phosph. plate]	116	-/-	18	Slices	-	Microscopy	3	-	-	0.58-0.68	-	-	-
Wenzel et al. (1995)[sensor]	116	-/-	18	Slices	-	Microscopy	3	-	-	0.42-0.7	-	-	-
Moystad et al. (1996) [phosp. plate]	50	25/25	18	Slices	-	Microscopy	17	-	-	0.78	-	-	0.79
Svanaes et al. (1996) [phosp. plate]	50	25/25	18	Hemisection	-	Microscopy	3	-	-	0.72	-	-	0.68
Huysmans et al. (1997) [phosph. plate]	220	-/-	18	Slices	-	Stereomicroscopy	1	26-33	93-95	0.61-0.69	-	-	-
White et al. (1997) [sensor]	320[surf]	80/80	18	Slices	-	Microscopy	1	34.2	91.5-	-	-	-	-
Ariji et al. (1998) [sensor]	118	-/-	1	Slices	-	Microscopy	1	29	87	-	35	96	-
Wenzel et al. (1999) [phosp plate]	230 [surf]	-/-	18	Slices/Groun	-	Microscopy	3	-	-	0.60-0.71	-	-	-
Abreu et al. (1999) [sensor]	40	20/20	18	Slices	-	Microscopy	9	-	-	0.77	-	-	-
Svanaes et al. (2000) [phosp. plate]	120	61/59	18	Slices	-	Microscopy	1	48	94	0.77	-	-	-
Abreu et al. (2001) [sensor]	40	20/20	18	Slices	-	Microscopy	1	-	-	0.82-0.84	-	-	-
Nair et al. (2001) [sensor]	46	-/-	18	Slices	-	Microscopy	9	69	85	0.73	82	88	-
Hintze et al. (2002a) [phosph. plate]	190	159/31	18	Slices	-	Microscopy	4	-	-	0.64-0.69	-	-	-
Hintze et al. (2002b) [sensor]	177	146/31	18	Slices	-	Microscopy	4	-	-	0.64-0.71	-	-	-
Li et al. (2002) [sensor]	40	-/-	18	Hemi/Slices	-	Microscopy	3	-	-	0.57	-	-	0.84
Matsuda et al. (2002) [sensor]	30	-/30	18	-	-	Micro-CT	nr	-	-	0.66-0.78	-	-	-
Wenzel et al. (2002) [sensor]	363 [surf]	-/-	10	Slices	-	Microscopy	3	22-58	85-99	-	-	-	-
Haak et al. (2003) [sensor]	160	-/-	13	Slices	-	Microscopy	10	38	93	-	33	95	-
Moystad et al. (2003) [phosph. plate]	120	61/59	18	Hemisection	-	Microscopy	1	-	-	0.84	-	-	-
Pabla et al. (2003) [phosph. plate]	41	22/19	18	Slices	-	Microscopy	9	-	-	0.86	-	-	-
Jacobsen et al. (2004) [sensor]	177	-/-	nr	Slices	-	Microscopy	nr	-	-	-	-	-	-
Jacobsen et al. (2004) [phosph. plate]	177	-/-	nr	Slices	-	Microscopy	nr	-	-	-	-	-	-
De Araujo et al. (2005) [Sensor]	52	-/52	1	Slices	-	Microscopy	1	-	-	0.74-0.88	-	-	-
Khan et al. (2005) [sensor]	40	20/20	18	Slices	-	Microscopy	9	-	-	0.80	-	-	-
Rocha et al. (2005) [sensor]	48	-/-	11	Slices	-	Microscopy	nr	56-71	86-100	-	-	-	-
Haak et al. (2005) [sensor]	320[surf]	-/-	13	Slices	-	Microscopy	11	-	-	0.69	-	-	0.74
Akdeniz et al. (2006)[phosp.plate]	30	16/14	nr	Slices	-	Microscopy	nr	-	-	-	-	-	-

Digital bitewing radiography examination	Study material		Diagnostics	Validation methodology				Validity					
In vitro validation studies on proximal surfaces	Teeth (N)	Molars/ PM (N)	Radiographic Criteria[1]	Hard tissue processing	Caries Staining	Visualisation /magnification	Scoring criteria[2]	Caries detection level			Dentin caries detection level		
								SE (%)	SP (%)	Az (ROC)	SE (%)	SP (%)	Az (ROC)
Kutcher et al. (2006)[phosp.plate]	40	19/21	18	Slices	-	Microscopy	9	43-54	0.97-0.98	0.79-0.87	-	-	-
Berkhout et al.(2007)[phosp.plate]	65	0/65	9	Slices	-	Microscopy	2	-	-	-	-	-	-
Berkhout et al. (2007) [sensor]	65	0/65	9	Slices	-	Microscopy	2	-	-	-	-	-	-
Haiter-Neto et al. (2007) [Phosp. plate]	80	40/40	18	Slices	new!	Microscopy		15/22	91/85	-	-	-	-
Haiter-Neto et al. (2007) [sensor]	80	40/40	18	Slices	new!	Microscopy		19/23	90/90	-	-	-	-
Castro et al. (2007) [sensor]	174	-/-	20	Slices	-	Microscopy	nr	-	-	0.73	-	-	-
Li et al. (2007) [phosp.plate]	90	-/90	18	Slices	-	Microscopy	2	-	-	0.71/0.76	-	-	0.78/0.83
Peker et al. (2007) [Sensor]	48	24/24	18	Hemisection	fuchsin	Microscopy	5	-	-	0.79	-	-	-
Wenzel et al. (2007a) [phosph plate]	80	40/40	14	Slices	-	Microscopy	4	-	-	-	-	-	-
Wenzel et al. (2007a) [Sensor]	80	40/40	14	Slices	-	Microscopy	4	-	-	-	-	-	-
Wenzel et al. (2007b) [phosph plate]	80	40/40	13	Slices	-	Microscopy	5	16-26	85-94	-	-	-	-
Wenzel et al. (2007b) [Sensor]	80	40/40	13	Slices	-	Microscopy	5	19-20	92-93	-	-	-	-
Forner-Navarro et al. (2008) [Sensor]	192	-/-	1	Abrasion	-	Photomacroscope	1	17/20	85/80	-	5/7	95/90	-
Haiter Neto et al. (2008) [phosph plate]	80	40/40	18+9	Slices	-	Microscopy	3	17	91	-	-	-	-
Li et al. (2008) [phosp.plate]	72[surf]	72/-	18	Slices	-	Microscopy	14	-	-	0.60-0.74	-	-	-
Rockenbach et al. (2008) [Phosphor plate]	75	51/24	12	Hemisection	-	Microscopy	4	51.4-56.9	75-85.7	-	-	-	-
Schulte et al. (2008) [Sensor]	30	-/30	1	Slices/Groun	-	Photography	?	-	-	-	46-53	96-100	-
Schulte et al. (2008) [Phosphor plate]	30	-/30	1	Slices/Groun	-	Photography	?	-	-	-	27-53	96-100	-
Schulze et al. (2008) [Sensor]	100	44/23	18	Slices	-	Microscopy	4	15	91	-	19	91	-
Haiter Neto et al. (2009) [phosph plate]	80	40/40	22	Slices	-	Microscopy	3	29	86	-	-	-	-
Hellen-Halme et al. (2009) [Sensor]	100	60/40	18	Slices	-	Microscopy	14	-	-	0.60/0.61	-	-	0.71/0.73
Isidor et al. (2009) [Phosphor plate]	80	40/40	14	Slices	-	Microscopy	13	13-17	93-96	-	-	-	-
Peker et al. (2009)[Sensor]	48	-/-	1	Slices	-	Microscopy	16	-	-	0.76/0.79	-	-	-
Shi et al. (2009) [Sensor]	130	-/130	18	Hemi/Slices	-	Microscopy	1	-	-	0.61	-	-	0.82
Shi et al. (2009) [Phosphor plate]	130	-/130	18	Hemi/Slices	-	Microscopy	1	-	-	0.67	-	-	0.83
Young et al. (2009) [Sensor]	100[surf]	-/-		-	-	Micro-CT	1	-	-	0.18-0.96	-	-	0.33-0.96
Booshehry et al. (2010) [Sensor]	102 [surf]	-/-	22	Slices	-	Microscopy	15	66.7	60	-	-	-	-
Hellen-Halme et al. (2010) [Sensor]	100	60/40	18	Slices	-	Microscopy	14	-	-	0.60	-	-	0.76
Li et al. (2010) [Phosphor plate]	35	19/16	18	Slices	-	Microsopy	2	-	-	0.57	-	-	-
Mitropoulos et al. (2010) [Sensor]	20	8/12	9	Slices	-	Microscopy	2	46/50	100/94	0.73/0.72	52/50	87/81	0.76/0.74

Digital bitewing radiography examination	Study material		Diagnostics	Validation methodology				Validity					
In vitro validation studies on proximal surfaces	Teeth (N)	Molars/ PM (N)	Radiographic Criteria[1]	Hard tissue processing	Caries Staining	Visualisation /magnification	Scoring criteria[2]	Caries detection level			Dentin caries detection level		
								SE (%)	SP (%)	Az (ROC)	SE (%)	SP (%)	Az (ROC)
Pontual et al. (2010) phosphor plate	80	40/40	9	Slices	-	Microscopy	2	14-16	89-94	-	-	-	-
Senel et al. (2010) Sensor	138	-/-	6	Slices	-	Scanner	4	-	-	0.75-0.78	-	-	-
Senel et al. (2010) phosphor plate	138	-/-	6	Slices	-	Scanner	4	-	-	0.80-0.81	-	-	-
Behere et al. (2011) Sensor	100 surf	-/-	10	Slices	-	Photography	3	33	96	-	100	96	-
Bottenberg et al. (2011) Sensor	116 surf	-/-	2	Slices/Groun	-	Microscopy/Photo	1	85-95	9-40	0.53-0.58	-	-	-
Hellen-Halme et al. (2011) Sensor	100	60/40	18	Slices	-	Microscopy	14	-	-	0.60/0.59	-	-	0.67/0.71
Kayipmaz et al.(2011) Phosphor plate	72	27/45	18	Slices	-	Microscopy	1	-	-	0.68	-	-	-
Zhang et al. (2011) phosphor plate	78 surf	-/-	18	Slices	-	Microscopy	2	-	-	0.52	-	-	-
Abesi et al. (2012) sensor	66 surf	-/-	6	Slices	-	Microscopy	4	15	96	-	-	-	-
Abesi et al. (2012) phosph. plate	66 surf	-/-	6	Slices	-	Microscopy	4	23	98	-	-	-	-
Astvaldsdottr et al. (2012) sensor	56	-/56	1	Slices	-	Microscopy/M-rad	1	22-64	74-100	0.52-0.71	40-60	92-100	0.78-0.98
Cheng et al. (2012) phosph. plate	45	-/-	18	Slices	-	Microscopy	7	-	-	0.59	-	-	-
Pakkala et al. (2012) phosph. plate	80	40/40	9	Slices	-	Microscopy	?	16-25	89-95	0.64-0.67	-	-	-
Minston et al. (2013) sensor	46	-/-	18	Slices	-	Microscopy	3	-	-	0.52/0.55	-	-	0.73/0.74
Pontual et al. (2013) sensor	160 surf	-/-	18	Slices	-	Microscopy	3	-	-	0.51-0.62	-	-	-
Pontual et al. (2013) phosph. plate	160 surf	-/-	18	Slices	-	Microscopy	3	-	-	0.49-0.59	-	-	-
Krzyzostaniak et al. (2014) phosph. plate	135	68/67	4	Slices	-	Microscopy	6	-	-	0.67	-	-	-
Zayet et al. (2014) phosph. plate	75	-/-	1	Slices	-	Microscopy	1	90/90	55/70	0.73/0.83	100/100	48.1/44.2	-
Ko et al. (2015) sensor	95	-/-	1	Slices	-	Microscopy	1	71	89	0.80	50	94	0.72
Melo et al. (2015) phosph. plate	20	10/10	18	Slices	-	Microscopy	3	-	-	0.49-0.55	-	-	-
Miri et al. (2015) sensor	80	-/80	18	Slices	hematoxylin/ eosin	Microscopy		-	-	-	72.5	90	80.9
Vivek et al. (2015) phosph. plate	50	-/-	6	Slices	-	Microscopy	4	81	74	0.85	-	-	-
Barbosa et al. (2015) phosph. plate	40	20/20	18	Slices	-	Microscopy	3	-	-	-	-	-	-
Abdinian et al. (2015) phosph. plate	100*	-/-	18	Slices	-	Microscopy	4	74.1	91.6	0.80	-	-	-
Safi et al. (2015) phosph. plate	42	-/-	21	Slices	-	Microscopy	4	56.8	94.9	-	-	-	-
Bozdemir et al. (2016) phosph. plate	156 surfaces	-/-	10	Slices	-	Microscopy	3	32.5/36.1	97.3/95.9	0.65/0.66	28.2/28.2	99.1/98.3	0.74/0.73
Milosavljevic (2016) sensor	100	60/40	18	Slices	-	Microscopy	14	-	-	0.55-0.61	-	-	0.63-0.70
Dehghani et al. (2017) phosph. plate	50	16/34	18	Slices	-	Microscopy	1	27.7/44.4	76.6/78.0	0.70/0.68	-	-	-
Dehghani et al. (2017) sensor	50	16/34	18	Slices	-	Microscopy	1	5.5	82.9/73.1	0.69/0.61		-	-

Digital bitewing radiography examination	Study material		Diagnostics	Validation methodology				Validity					
In vitro validation studies on proximal surfaces	Teeth (N)	Molars/ PM (N)	Radiographic Criteria[1]	Hard tissue processing	Caries Staining	Visualisation /magnification	Scoring criteria[2]	Caries detection level			Dentin caries detection level		
								SE (%)	SP (%)	Az (ROC)	SE (%)	SP (%)	Az (ROC)
Ghoncheh et al. (2017) phosph. plate	52	26/26	18	Slices	-	Microscopy	10	30.68	-	-	80.63	90.24	-
Nascimento et al. (2018) sensor	40	20/20	18	-	-	Micro-CT	3	45	84	0.67	-	-	-
Adibi et al. (2018) phosph. plate	240surf	-/-	18	Slices	-	Microscopy	12	36.5-37.7	96.3-97.1	0.83-0.88	-	-	-

[1]See Table S1b; [2]See Table S1e; nr-not reported *canines; **incisors

Table S5b Results of Systematic Literature Review for in vivo digital radiography validation studies on proximal surfaces

Digital bitewing radiography examination	Study material			Diagnostics	In vivo validation methodology			Validity					
								Caries detection level			Dentin caries detection level		
In vivo validation studies on proximal surfaces	Patients (N)	Age (years)	Teeth (N)	Radiographic Criteria	Histology technique	Validation	Reference method	SE (%)	SP (%)	Az (ROC)	SE (%)	SP (%)	Az (ROC)
Huth et al. (2010) sensor	117	8-54	117	1	Cav.Prep.	Visual/Radiography	1	-	-	-	-	-	-
Li et al. (2010) phosph. plate	11	nr	35	18	Histo prepa	*in vivo*	2	-	-	0.60	-	-	-
Kühnisch et al. (2016) sensor	85	25	127	1	Cav.Prep.	Visual/Radiography		-	-	-	96	-	0.98
Baltacioglu et al. (2017) phosph. plate	26	28.1-32.4	52 (32)	18	Cav.Prep.	Visually	nr	-	-	-	-	-	0.63-0.87
Jablonski- Momeni et al. (2017) sensor	18	29.5	193 surfaces	9	nr	Tooth separation	2	-	-	-	-	-	-
Menem et al. (2017) sensor	30	18-37	90 surfaces	6	nr	Tooth separation	3	55	93	0.81			
Ozkan et al. (2017) phosph. plate	157	12-18	161 surfaces	8	Cav.Prep.	Visual/Radiography	4	-	-	-	83	60	71

nr-not reported

Table S5c Reproducibility/Reliability results of Systematic Literature Review for in vitro digital bitewing studies on proximal surfaces

Digital bitewing radiography examination	Study material			Diagnostics	Reproducibility/Reliability detection & diagnostic methods		
In vitro validation studies on proximal surfaces	Examiners (N)	Teeth (N)	Molars/PM (N)	Radiographic Criteria	Reproducibility/Reliability testing	Intraexaminer	Interexaminer
Hintze et al. (1994)	2	66	-/-	18	nr	-	-
Wenzel et al. (1995)	6	116	-/-	18	nr	-	-
Moystad et al. (1996)	10	50	25/25	18	nr	-	-
Svanaes et al. (1996)	10	50	25/25	18	nr	-	-
Huysmans et al. (1997)	3	220	-/-	18	Cohen's Kappa	-	0.53-0.69
White et al. (1997)	16	160[surfaces]	80/80	18	nr	-	-
Ariji et al. (1998)	6	118	-/-	1	nr	-	-
Wenzel et al. (1999)	5	230[surfaces]		18	nr	-	-
Abreu et al. (1999)	8	40	20/20	18	ICC	0.67	0.54
Svanaes et al. (2000)	9	120	61/59	18	nr	-	-
Abreu et al. (2001)	6	40	20/20	18	nr	-	-
Nair et al. (2001)	8	46	-/-	18	Cohen's Kappa	0.66	0.42
Hintze et al. (2002a)	4	190	159/31	18	nr	-	-
Hintze et al. (2002b)	4	177	143/31	18	nr	-	-
Li et al. (2002)	10	40	-/-	18	nr	-	-
Matsuda et al. (2002)	3	30	-/30	18	nr	-	-
Wenzel et al. (2002)	4	363 [surfaces]	-/-	10	nr	-	-
Haak et al. (2003)	5	160	-/-	13	nr	-	-
Moystad et al. (2003)	7	120	61/59	18	nr	-	-
Pabla et al. (2003)	7	41	22/19	18	nr	-	-
Jacobsen et al. (2004)	4	177	-/-	nr	nr	-	-
De Araujo et al. (2005)	4	52	-/52	1	nr	-	-
Khan et al. (2005)	4	40	20/20	18	nr	-	-
Rocha et al. (2005)	14	48	-/-	11	Cohen's Kappa	0.38-0.54	-
Haak et al. (2005)	5	320[surf]	-/-	13	nr	-	-
Akdeniz et al. (2006)	2	30	16/14	nr	Bland Altman	-	-
Kutcher et al. (2006)	6	40	19/21	18	nr	-	-
Berkhout et al. (2007)	5	65	0/65	9	nr	-	-
Haiter-Neto et al. (2007)	8	80	40/40	18	nr	-	-
Castro et al. (2007)	7	174	-/-	20	Pearson corr. coeff./ Kendall's	-0.02	0.43
Peker et al. (2007)	2	48	24/24	18	nr	-	-
Wenzel et al. (2007a)	8	80	40/40	14	nr	-	-
Wenzel et al. (2007b)	6	80	40/40	5	nr	-	-
Forner-Navarro et al. (2008)	1	192	-/-	1	Cohen's Kappa	0.98	-
Haiter Neto et al. (2008)	6	80	40/40	18+9	nr	-	-
Li et al. (2008)	10	72 [surf]	72/-	18	nr	-	-
Rockenbach et al. (2008)	1	75	51/24	12	Kendall's test	0.79-0.86	-

Digital bitewing radiography examination	Study material			Diagnostics	Reproducibility/Reliability detection & diagnostic methods		
In vitro validation studies on proximal surfaces	Examiners (N)	Teeth (N)	Molars/PM (N)	Radiographic Criteria	Reproducibility/Reliability testing	Intraexaminer	Interexaminer
Schulte et al. (2008) Sensor	4	30	-/30	?	Cohen's Kappa	0.47-0.57	-
Schulte et al. (2008) Phosphor plate	4	30	-/30	?	Cohen's Kappa	0.47-0.57	-
Schulze et al. (2008)	10	100	44/23	18	Cohen's Kappa	0.33-0.52	0.35-0.40
Haiter Neto et al. (2009)	6	80	40/40	22	nr	-	-
Hellen-Halme et al. (2009)	7	100	60/40	18	Cohen's Kappa	0.30-0.66	-
Isidor et al. (2009)	7	80	40/40	14	nr	-	-
Peker et al. (2009)	3	48	-/-	1	nr	-	-
Shi et al (2009)	7	130	-/130	18	nr	-	-
Young et al. (2009)	8	100surf	-/-	nr	nr	-	-
Booshehry et al. (2010)	3	102 surf	-/-	22	Cohen's Kappa	-	0.33-0.70
Hellen-Halme et al. (2010)	7	100	60/40	18	Cohen's Kappa	0.63	-
Li et al. (2010)	6	35	19/16	18	nr	-	-
Mitropoulos et al. (2010)	2	20	-/-	9	Cohen's Kappa	-	0.72
Pontual et al. (2010)	17	80	40/40	9	nr	-	-
Senel et al. (2010) sensor	3	138	-/-	6	Cohen's Kappa	0.79-0.86	-
Senel et al. (2010) phosphor plate	3	138	-/-	6	Cohen's Kappa	0.81-0.88	-
Behere et al. (2011)	3	100surfaces	-/-	10	Cohen's Kappa	-	-
Bottenberg et al. (2011)	3	116surfaces	-/-	2	Cohen's Kappa	0.72-0.80	-
Hellen-Halme et al. (2011)	7	100	60/40	18	Cohen's Kappa	0.85	-
Kayipmaz et al. (2011)	2	72	27/45	18	nr	-	-
Zhang et al. (2011)	7	78surfaces	-/-	18	paired t test	-	-
Abesi et al. (2012) sensor	4	66surfaces	-/-	6	nr	-	-
Abesi et al. (2012) phosphor plate	4	66surfaces	-/-	6	nr	-	-
Astvaldsdottr et al. (2012)	8	56	-/56	1	Cohen's Kappa	0.79	0.67-0.78
Cheng et al. (2012)	8	45	-/-	18	nr	-	-
Pakkala et al. (2012)	2	80	40/40	9	nr	-	-
Minston et al. (2013)	20	46	-/-	18	nr	-	-
Pontual et al. (2013) sensor	6	160 surfaces	-/-	18	nr	-	-
Pontual et al. (2013) hosphor plate	6	160 surfaces	-/-	18	nr	-	-
Krzyzostaniak et al. (2014)	2	135	68/67	4	nr	-	-
Zayet et al. (2014)	6	75	-/-	1	Cohen's Kappa	0.79/0.81	0.80/0.77
Ko et al. (2015)	1	95	-/-	1	ICC	0.88	-
Melo et al. (2015)	3	20	10/10	18	Cohen's Kappa	-	-
Miri et al. (2015)	4	80	-/80	18	nr	-	-
Vivek et al. (2015)	1	50	-/-	6	nr	-	-
Barbosa et al. (2015)	36	40	20/20	18	nr	-	-
Abdinian et al. (2015)	2	100*	-/-	18	Cohen's Kappa	-	-
Safi et al. (2015)	4	42	-/-	21	nr	-	-
Bozdemir et al. (2016)	2	156surfaces	-/-	10	Cohen's Kappa	-	0.75

Digital bitewing radiography examination	Study material			Diagnostics	Reproducibility/Reliability detection & diagnostic methods		
In vitro validation studies on proximal surfaces	Examiners (N)	Teeth (N)	Molars/PM (N)	Radiographic Criteria	Reproducibility/Reliability testing	Intraexaminer	Interexaminer
Milosavljevic (2016)	7	100	60/40	18	Cohen's Kappa	0.14-0.54	-
Dehghani et al. (2017)	2	50	16/34	18	nr	-	-
Ghoncheh et al. (2017)	4	52	26/26	18	nr	-	-
Nascimento et al. (2018)	5	40	20/20	18	Cohen's Kappa	0.72	0.46
Adibi et al. (2018)	2	240 surfaces	-/-	18	Cohen's Kappa	0.81-0.95	0.77-0.85

Table S5d Reproducibility/Reliability results of Systematic Literature Review for in vivo digital bitewing studies on proximal surfaces

Digital bitewing radiography examination	Study material				Diagnostics	Reproducibility/Reliability detection & diagnostic methods		
In vivo validation studies on proximal surfaces	Patients (N)	Examiners (N)	Teeth (N)	Molars/PM (N)	Radiographic Criteria	Reproducibility/Reliability testing	Intraexaminer	Interexaminer
Huth et al. (2010)	117	4	117	-/-	1	Cohen's Kappa	0.73-0.89	0.74-0.84
Li et al. (2010)	11	6	35	19/16	18	nr	-	-
Kühnisch et al. (2016)	85	2	127	81/45	1	nr	-	-
Baltacioglu et al. (2017)	26	2	52 (32)	-/-	18	Cohen's Kappa	0.55/0.65	0.62/0.67
Jablonski- Momeni et al. (2017)	18	3	193 surfaces	95/98	9	nr	-	-
Menem et al. (2017)	30	1	90 surfaces	63/27	6	Cohen's Kappa	0.81	-
Ozkan et al. (2017)	157	2	161 surfaces	-/-	2	Cohen's Kappa	0.38/0.63	0.35

nr-not reported

Table S5e Risk of bias assessment for digital bitewing radiography of in vitro validation studies on proximal surfaces

Digital bitewing of *in vitro* validation studies on proximal surfaces	Signaling questions															
	Selection bias				Index test bias			Reference test bias				Verification bias			Outcome bias	
	Patient selection	Teeth selection	Caries Spectrum	Sample size	Test Criteria	Blinding bias	Calibration bias	Test Criteria	Blinding bias	Calibration bias	Incorporation bias	Partial ver. bias	Differential ver. bias	Bias in the Analysis	Validity bias	Reproducibility bias
Hintze et al. (1994)	x	?	-	-	-	+	-	+	-	-	+	+	+	+	?	-
Wenzel et al. (1995)	x	?	?	-	-	+	-	+	+	-	+	+	+	+	-	-
Moystad et al. (1996)	x	+	?	-	-	?	?	+	-	-	+	+	+	+	?	-
Svanaes et al. (1996)	x	+	?	-	-	+	-	+	-	-	+	+	+	+	?	-
Huysmans et al. (1997)	x	-	-	-	-	+	-	+	-	-	+	+	+	+	?	?
White et al. (1997)	x	-	+	-	-	+	-	+	?	-	+	+	+	+	?	-
Ariji et al. (1998)	x	?	-	-	+	?	-	+	?	-	+	+	+	+	?	-
Wenzel et al. (1999)	x	?	-	-	-	?	-	+	?	-	+	+	+	+	?	-
Abreu et al. (1999)	x	+	-	-	-	-	-	+	+	-	+	+	+	+	-	-
Svanaes et al. (2000)	x	+	-	-	-	+	-	+	?	-	+	+	+	+	?	-
Abreu et al. (2001)	x	+	+	-	-	+	?	+	?	-	+	+	+	+	?	-
Nair et al. (2001)	x	?	-	-	-	+	?	+	?	-	+	+	+	+	?	+
Hintze et al. (2002a)	x	+	-	-	-	+	-	+	+	?	+	+	+	+	?	-
Hintze et al. (2002b)	x	+	-	-	-	+	-	+	+	-	+	+	+	+	?	-
Li et al. (2002)	x	-	-	-	-	+	-	+	?	-	+	+	+	+	?	-
Matsuda et al. (2002)	x	-	-	-	-	+	-	-	-	-	+	+	+	+	?	-
Wenzel et al. (2002)	x	?	-	-	+	-	-	+	-	-	+	+	+	+	?	-
Haak et al. (2003)	x	+	?	-	+	+	-	+	?	-	+	+	+	?	+	-
Moystad et al. (2003)	x	+	-	-	-	+	-	+	-	-	+	+	+	+	?	-

Digital bitewing of *in vitro* validation studies on proximal surfaces	Signaling questions															
	Selection bias				Index test bias			Reference test bias				Verification bias			Outcome bias	
	Patient selection	Teeth selection	Caries Spectrum	Sample size	Test Criteria	Blinding bias	Calibration bias	Test Criteria	Blinding bias	Calibration bias	Incorporation bias	Partial ver. bias	Differential ver. bias	Bias in the Analysis	Validity bias	Reproducibility bias
Pabla et al. (2003)	x	+	-	-	-	+	-	+	+	-	+	+	+	+	?	-
Jacobsen et al. (2004)	x	?	-	-	-	?	-	-	?	-	+	-	+	+	-	-
De Araujo et al. (2005)	x	-	+	-	+	-	+	+	-	-	+	+	+	+	?	-
Khan et al. (2005)	x	+	+	-	-	+	?	+	+	-	+	+	+	+	?	-
Rocha et al. (2005)	x	-	-	-	-	+	-	-	-	-	?	+	+	+	?	?
Haak et al. (2005)	x	?	-	-	+	+	-	+	+	-	+	+	+	+	?	-
Akdeniz et al. (2006)	x	+	?	-	-	-	-	-	-	-	+	+	+	+	-	-
Kutcher et al. (2006)	x	+	-	-	-	+	-	+	+	-	+	+	+	+	?	-
Berkhout et al. (2007)	x	-	+	-	+	-	-	+	-	-	+	+	+	+	-	-
Haiter-Neto et al. (2007)	x	+	+	-	-	-	-	+	?	-	+	+	+	+	?	-
Castro et al. (2007)	x	?	?	-	-	+	-	+	?	-	+	+	+	+	?	?
Li et al. (2007)	x	-	-	-	-	+	-	+	+	-	+	+	+	+	?	-
Peker et al. (2007)	x	+	?	-	-	-	-	+	-	-	+	+	+	+	?	-
Wenzel et al. (2007a)	x	+	-	-	+	+	-	+	+	-	+	+	+	+	?	-
Wenzel et al. (2007b)	x	+	-	-	+	+	-	+	+	-	+	+	+	+	?	-
Forner-Navarro et al. (2008)	x	+	-	+	+	-	-	+	-	-	+	+	+	+	?	-
Haiter Neto et al. (2008)	x	+	+	-	-	+	-	+	+	?	+	+	+	+	?	-
Li et al. (2008)	x	-	-	-	-	+	-	+	+	-	+	+	+	+	?	-
Rockenbach et al. (2008)	x	+	-	-	+	+	-	+	+	-	+	+	+	+	?	?
Schulte et al. (2008)	x	-	+	-	+	+	-	?	-	-	+	+	+	+	?	?

Digital bitewing of *in vitro* validation studies on proximal surfaces	Signaling questions															
	Selection bias				Index test bias			Reference test bias				Verification bias			Outcome bias	
	Patient selection	Teeth selection	Caries Spectrum	Sample size	Test Criteria	Blinding bias	Calibration bias	Test Criteria	Blinding bias	Calibration bias	Incorporation bias	Partial ver. bias	Differential ver. bias	Bias in the Analysis	Validity bias	Reproducibility bias
Schulze et al. (2008)	x	-	?	-	-	-	-	+	-	-	+	+	+	+	?	+
Haiter Neto et al. (2009)	x	+	?	-	+	+	+	+	+	+	+	+	+	+	?	-
Hellen-Halme et al. (2009)	x	+	+	-	-	+	-	+	+	-	+	+	+	+	?	?
Isidor et al. (2009)	x	+	?	-	+	+	?	+	+	-	+	+	+	+	?	-
Peker et al. (2009)	x	?	+	-	+	+	-	+	-	+	+	+	+	+	?	-
Shi et al. (2009)	x	-	?	-	-	+	-	+	+	-	+	+	+	+	?	-
Young et al. (2009)	x	?	-	-	-	+	?	+	+	-	+	+	+	+	?	-
Booshehry et al. (2010)		-	?	-	+	+	-	+	+	-	+	+	+	+	?	?
Hellen-Halme et al. (2010)	x	+	+	-	-	+	-	+	+	-	+	+	+	+	?	?
Li et al. (2010)	x	+	-	-	+	+	-	+	+	-	+	+	+	+	?	-
Mitropoulos et al. (2010)	x	+	-	-	+	+	?	+	?	?	+	+	+	+	+	?
Pontual et al. (2010)	x	+	?	-	+	+	-	+	-	-	+	+	+	+	+	?
Senel et al. (2010)	x	?	?	-	+	?	-	+	?	-	+	+	+	+	+	+
Behere et al. (2011)	x	?	-	-	+	+	?	+	-	-	+	+	+	+	?	?
Bottenberg et al. (2011)	x	?	?	-	+	+	-	+	+	-	+	+	+	-	-	-
Hellen-Halme et al. (2011)	x	+	+	-	-	+	-	+	+	-	+	+	+	+	?	?
Kayipmaz et al. (2011)	x	+	-	-	-	-	-	+	-	-	+	+	+	+	?	-
Zhang et al. (2011)	x	-	-	-	-	+	-	+	+	-	+	+	+	+	?	?
Abesi et al. (2012)	x	?	?	-	+	+	-	+	+	-	+	+	+	+	-	-
Astvaldsdottir et al. (2012)	x	?	?	-	+	+	-	+	+	?	+	+	+	+	+	+

Digital bitewing of *in vitro* validation studies on proximal surfaces	Signaling questions															
	Selection bias				Index test bias			Reference test bias				Verification bias			Outcome bias	
	Patient selection	Teeth selection	Caries Spectrum	Sample size	Test Criteria	Blinding bias	Calibration bias	Test Criteria	Blinding bias	Calibration bias	Incorporation bias	Partial ver. bias	Differential ver. bias	Bias in the Analysis	Validity bias	Reproducibility bias
Cheng et al. (2012)	x	?	?	-	-	+	-	+	+	?	+	+	+	+	?	+
Pakkala et al. (2012)	x	+	?	-	+	?	?	?	?	-	+	+	+	+	+	-
Minston et al. (2013)	x	?	-	-	-	+	-	+	+	-	+	+	+	+	?	-
Pontual et al. (2013)	x	-	-	-	-	+	+	+	+	-	+	+	+	+	?	-
Krzyzostaniak et al. (2014)	x	+	-	-	+	+	-	+	+	-	+	+	+	+	-	-
Zayet et al. (2014)	x	?	?	-	+	+	-	+	-	-	+	+	+	+	+	+
Ko et al. (2015)	x	?	-	-	+	-	?	+	-	-	+	-	-	-	?	?
Melo et al. (2015)	x	+	-	-	-	+	+	+	?	?	+	+	+	+	?	?
Miri et al. (2015)	x	-	-	-	-	-	-	-	-	-	+	+	+	+	+	-
Vivek et al. (2015)	x	-	-	-	+	+	-	+	+	-	+	+	+	+	+	-
Barbosa et al. (2015)	x	+	-	-	-	+	?	+	+	?	+	+	+	+	?	-
Abdinian et al. (2015)	x	-	?	-	-	+	-	+	-	-	+	+	+	+	+	?
Safi et al. (2015)	x	?	?	-	+	-	-	+	-	+	+	+	+	+	+	-
Bozdemir et al. (2016)	x	-	?	-	+	-	-	+	+	-	+	+	+	+	+	?
Milosavljevic (2016)	x	+	+	-	-	+	?	+	+	-	+	+	+	+	?	?
Dehghani et al. (2017)	x	+	?	-	-	+	-	+	+	-	+	+	+	+	+	-
Ghoncheh et al. (2017)	x	+	?	-	-	+	-	+	-	-	+	+	+	+	?	-
Nascimento et al. (2018)	x	+	-	-	-	+	-	?	-	-	+	+	+	+	+	-
Adibi et al. (2018)	x	?	?	-	+	+	?	+	+	?	+	+	+	+	+	+

Legend: +=Low risk of bias (Yes); -=High risk of bias (Probably No, No); ?=Unclear (No information, Incomplete reporting, Probably Yes) x=Question for in vivo studies

Table S5f Risk of bias assessment for digital bitewing radiography of in vivo validation studies on proximal surfaces

Digital bitewing of *in vivo* validation studies on proximal surfaces	Signaling questions															
	Selection bias				Index test bias			Reference test bias				Verification bias			Outcome bias	
	Patient selection	Teeth selection	Caries Spectrum	Sample size	Test Criteria	Blinding bias	Calibration bias	Test Criteria	Blinding bias	Calibration bias	Incorporation bias	Partial ver. bias	Differential ver. bias	Bias in the Analysis	Validity bias	Reproducibility bias
Huth et al. (2010)	-	?	-	+	+	+	-	+	+	?	+	+	-	+	-	+
Li et al. (2010)	-	+	-	-	+	+	-	+	+	-	+	+	+	+	?	-
Kühnisch et al. (2016)	-	+	-	+	+	+	+	+	+	+	+	+	+	+	+	-
Baltacioglu et al. (2017)	+	?	-	+	-	+	+	-	-	-	-	-	-	-	?	+
Jablonski- Momeni et al. (2017)	?	+	-	+	+	-	?	+	-	+	+	+	+	+	+	-
Menem et al. (2017)	?	+	+	+	+	-	+	+	-	+	+	+	+	+	+	-
Ozkan et al. (2017)	-	?	-	+	+	-	+	+	-	-	+	+	+	+	+	+

Legend: +=Low risk of bias (*Yes*); -=High risk of bias (*Probably No*, *No*); ?=Unclear (*No information, Incomplete reporting, Probably Yes*)

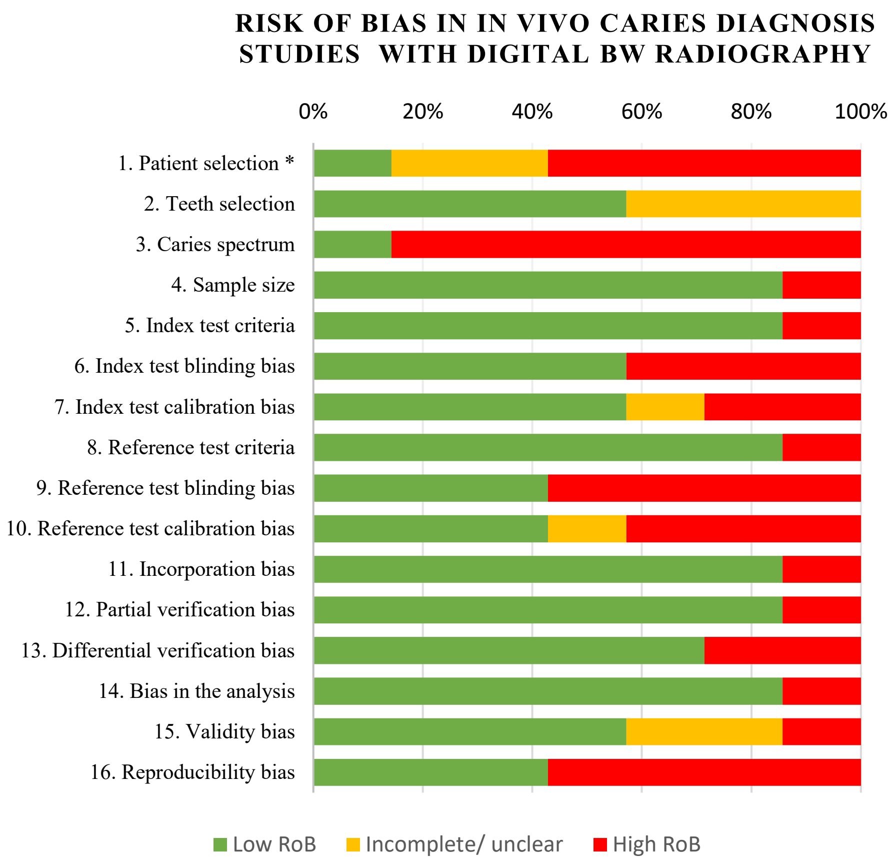

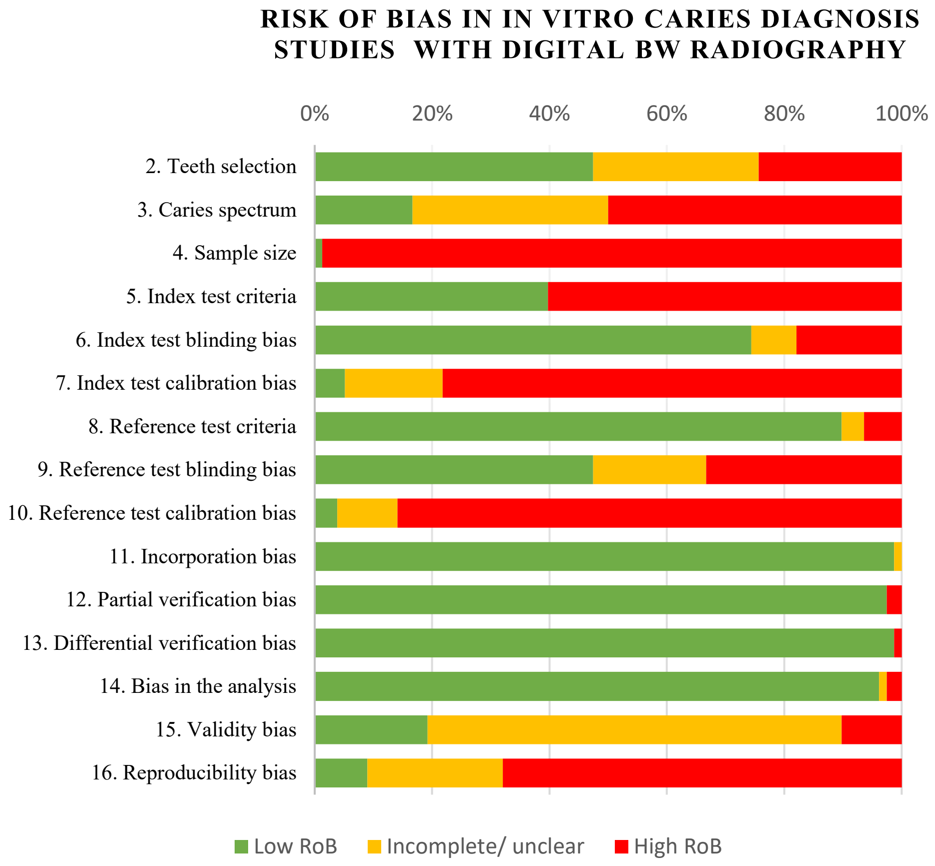

Figure S3 Risk of bias graph for in vivo and in vitro caries diagnostic studies with digital bitewing radiography: review authors' judgements about each risk of bias item presented as percentages across all included studies

* Item no 1 (Patient selection bias) is only available for clinical diagnostic studies

Table S5g Studies assessed for inclusion in the meta-analysis- digital bitewing radiography of in vitro validation studies on proximal surfaces

Studies that passed 1st round of selection	ITC	RTC	Sample size and caries distribution				Radiogr. mode	Cut-off	2nd round of selection	Cross-tabulation given	SE	SP	Az	3th round of selection
			Total	S	E	D								
Ariji et al. (1998)	1	1	232^{surf}	54	86	96	sensor	Caries detection level	included	no	29	87	-	included
								Dentin detection level	included	no	35	96	-	
Wenzel et al. 2002	10	3	363^{surf}	226	82	55	sensor	Caries detection level	included	no	30*	94*	-	included
								Dentin detection level		no	-	-		excluded
Haak et al. 2003	13	10	320^{surf}	107	94	119	sensor	Caries detection level	included	no	38	93	-	included
								Dentin detection level		no	33	95	-	
Haak et al. 2005	13	11	320^{surf}				sensor	Caries detection level	included	no	-	-	0.69	excluded
								Dentin detection level		no	-	-	0.74	
Wenzel et al. 2007a	14	4	160^{surf}	101	50	9	sensor	Caries detection level	included	yes	-	-	-	included
							phosph. plate	Caries detection level	included	yes	-	-	-	
Wenzel et al. 2007b	13	5	160^{surf}	101	50	9	sensor	Caries detection level	included	no	19/20	92/93	-	included
							phosph. plate	Caries detection level	included	no	16/26	92/93	-	included
Berkhout et al. 2007	9	2	65^{surf}	17	27	21	sensor	Caries detection level	included	yes	-	-	-	excluded
							phosph. plate	Dentin detection level	included	yes	-	-	-	
							sensor	Caries detection level	included	yes	-	-	-	
							phosph. plate	Dentin detection level	included	yes	-	-	-	
Forner-Navarro et al. 2008	1	1	384 $^{surf.}$	-	-	-	sensor	Caries detection level	included	no	17/20	85/80	-	included
								Dentin detection level	included	no	5/7	95/90	-	excluded
Haiter Neto et al. 2009	22	3	160 ^{surf}	101	56	3	phosph. plate	Caries detection level	included	no	29	86	-	included
Isidor et al. 2009	14	13	160^{surf}	100	50	10	phosph. plate	Caries detection level	included	no	13-17	93-96	-	included
Booshehry et al. 2010	22	15	102 ^{surf}	30	72		sensor	Caries detection level	included	no	74	55	-	included
Mitropoulos et al. 2010	9	2	40 $^{surf.}$	245	13	66	sensor	Caries detection level	included	yes	46/50	100/94	0.73/0.72	included
								Dentin detection level	included	yes	52/50	87/81	0.76/0.74	excluded
Behere et al. 2011	10	3	100^{surf}	76	20	4	sensor	Caries detection level	included	yes	33	96	-	included
								Dentin detection level	included	yes	100	96	-	
Pontual et al. 2010	9	2	152 $^{surf.}$	88	64	-	phosph. plate	Caries detection level	included	no	14-15-16	93-94-89	-	included
Senel et al. 2010	6	4	276 $^{surf.}$	142	40	94	sensor	Caries detection level	included	no	56*	95*	0.75-0.78	included

							phosph. plate	Caries detection level	included	no	48*	91*	0.80-0.81	
Bottenberg et al. 2011	2	1	116 surf.	61	55	-	sensor	Caries detection level	included	no	85-95	9-40	0.53-0.58	Excluded
Astvaldsdottr et al. 2012	1	1	97surf.	34	48	15	sensor	Caries detection level	included	no	22-64	74-100	0.52-0.71	included
								Dentin detection level	included	no	40-60	92-100	0.78-0.98	
Abesi et al. 2012	6	4	66 surf	46	13	7	sensor	Caries detection level	included	no	15	96	-	included
							phosph. plate	Caries detection level	included	no	23	98	-	
Krzyostaniak et al. 2014	4	6	270surf	157	74	39	phosph. plate	Caries detection level	included	no	-	-	0.67	excluded
Zayet et al. (2014)	1	1	150 surf	80	22	48	phosph. plate	Caries detection level	included	no	90/90	55/70	0.73/0.83	included
								Dentin detection level	included	no	100/100	48.1/44.2	-	
Safi et al. (2015)	21	4	84surf	54	11	19	phosph. plate	Caries detection level	included	yes	56.8	94.9	-	included
Vivek et al. 2015	6	4	100surf	42	16	42	phosph. plate	Caries detection level	included	no	81	74	0.85	included
Bozdemir et al. (2016)	10	3	156 surf.	73	44	39	phosph. plate	Caries detection level	included	no	32.5/36.1	97.3/95.9	0.65/0.66	included
								Dentin detection level	included	no	28.2/28.2	99.1/98.3	0.74/0.73	included

ITC-Index test criteria, RTC- Reference test criteria, S-Sound, E-Caries in enamel, D- Caries in dentin, *-calculated by us

Table S5h Studies assessed for inclusion in the meta-analysis- digital bitewing radiography of in vivo validation studies on proximal surfaces

Studies that passed 1st round of selection	ITC	RTC	Sample size and caries distribution				Radiogr. mode	Cut-off	2nd round of selection	Cross-tabulation given	SE	SP	Az	3th round of selection
			Total	S	E	D								
Jablonski- Momeni et al. 2017	9	2	193 surf	74	119	-	sensor	Caries detection level	included	yes	-	-	-	excluded
Menem et al. 2017	6	3	90 surf	30	60		sensor	Caries detection level	included	yes	55	93	0.81	included
Kühnisch et al. 2016	1	*	127 surf	0	0	127	sensor	Dentin caries detection level	included	yes	96	-	0.98	included
Ozkan et al. 2017	8	4	161 surf	0	5	156	phosph. plate	Dentin caries detection level	included	no	83	60	0.77	included

ITC-Index test criteria, RTC- Reference test criteria, S-Sound, E-Caries in enamel, D- Caries in dentin

Laser Fluorescence

Table S6a Results of Systematic Literature Review for in vitro LF validation studies on proximal surfaces

Laser Fluorescence examination	Study material		Diagnostics	Validation methodology				Validity					
								Caries detection level			Dentin caries detection level		
In vitro validation studies on proximal surfaces	Teeth (N)	Molars /PM (N)	LF criteria[1]	Hard tissue processing	Caries Staining	Visualisation /magnification	Scoring criteria[2]	SE (%)	SP (%)	Az (ROC)	SE (%)	SP (%)	Az (ROC)
DIAGNOdent 2095													
Tagtekin et al. (2008) cone shaped tip	38	-/-	3	Hemisection	-	Photography	9	-	24/57	-	71/85	-	-
Tagtekin et al. (2008) broad tip	38	-/-	3	Hemisection	-	Photography	9	-	24/76	-	48/52	-	-
DIAGNOdent Pen 2190													
Lussi et al. (2006) wedge shaped	75	75/-	4	Ground	-	Microscopy/Photo	1	88	92	-	89	82	-
Lussi et al. (2006) tapered wedge shaped	75	75/-	4	Ground	-	Microscopy/Photo	1	87	93	-	92	81	-
De Souza et al. (2014)	51**	-/-	1	Slices	Rhodamine B	Microscopy	1	80	96	0.93	94	95	0.98
Neuhaus et al. (2015)	120	120/-	2	Ground	-	Microscopy/Photo	4	47	78	-	39	84	-
Bozdemir et al. (2016)	156surf	-/-	4	Slices	-	Microscopy	3	94/77.1	60.3/75.3	0.88/0.79	79.5/76.9	83.8/76.9	0.84/0.79

[1]See Table S1c; [2]See Table S1e;**incisors

Table S6b Results of Systematic Literature Review for in vivo Laser Fluorescence validation studies on proximal surfaces

Laser Fluorescence examination	Study material			Diagnostics	In vivo validation methodology			Validity					
In vivo validation studies on proximal surfaces	Patients (N)	Age (years)	Teeth (N)	LF Criteria	Histology technique	Validation	Reference method	Caries detection level			Dentin caries detection level		
								SE (%)	SP (%)	Az (ROC)	SE (%)	SP (%)	Az (ROC)
DIAGNOdent Pen 2190													
Huth et al. (2010)	117	8-54	117	own	Cav.Prep.	Visual/Radiograph	1	68	70	-	60	84	-
Kühnisch et al. (2016)	85	25	127	1	Cav.Prep.	Visual/Radiography		-	-	-	67	-	0.82
Menem et al. (2017)	30	18/37	90[surfaces]	own	Cav.Prep.	Visual/Radiography	3	92	90	0.95	-	-	-
Ozkan et al. (2017)	157	12-18	161 [surfaces]	1	Cav.Prep.	Visual/Radiography	4	-	-	-	60	20	0.40

Table S6c Reproducibility/Reliability results of Systematic Literature Review for in vitro laser fluorescence studies on proximal surfaces

Laser Fluorescence examination	Study material			Diagnostics	Reproducibility/Reliability detection & diagnostic methods		
In vitro validation studies on proximal surfaces	Examiners (N)	Teeth (N)	Molars/PM (N)	LF Criteria	Reproducibility/Reliability testing	Intraexaminer	Interexaminer
DIAGNOdent 2095							
Tagtekin et al. (2008)[cone shaped tip]	2	38	-/-	3	Cohen's Kappa	0.36/0.38	0.38
Tagtekin et al. (2008) [broad tip]	2	38	-/-	3	Cohen's Kappa	0.20/0.38	0.43
DIAGNOdent Pen 2190							
Lussi et al. (2006)[wedge shaped]	5	75	75/-	4	Cohen's Kappa	0.74	-
Lussi et al. (2006)[tapered wedge shape]	5	75	75/-	4	Cohen's Kappa	0.82	-
De Souza et al. (2014)	2	51[**]	-/-	own	ICC	0.86/0.86	0.71
Neuhaus et al. (2015)	2	120	120/-	2	ICC	0.69-0.84	0.86
Bozdemir et al. (2016)	2	156[surf]	-/-	4	Cohen's Kappa	-	0.43

Table S6d Reproducibility/Reliability results of Systematic Literature Review for in vivo laser fluorescence studies on proximal surfaces

Laser Fluorescence examination	Study material				Diagnostics	Reproducibility/Reliability detection & diagnostic methods		
In vivo validation studies on proximal surfaces	Patients (N)	Examiners (N)	Teeth (N)	Molars/PM (N)	LF Criteria	Reproducibility/Reliability testing	Intraexaminer	Interexaminer
DIAGNOdent Pen 2190								
Huth et al. (2010)	117	4	117	-/-	own	ICC	0.84-0.98	0.97-0.98
Kühnisch et al. (2016)	85	2	127	46/81	1	nr	-	-
Menem et al. (2017)	30	1	90 surfaces	63/27	own	ICC	0.95	-
Ozkan et al. (2017)	157	12-18	161 surfaces	-/-	1	Cohen's Kappa	0.62/0.55	0.52

Table S6e Risk of bias assessment for laser fluorescence measurements of in vitro validation studies on proximal surfaces

Laser Fluorescence measurements of *in vitro* validation studies on proximal surfaces	Signaling questions															
	Selection bias				Index test bias			Reference test bias				Verification bias			Outcome bias	
	Patient selection	Teeth selection	Caries Spectrum	Sample size	Test Criteria	Blinding bias	Calibration bias	Test Criteria	Blinding bias	Calibration bias	Incorporation bias	Partial ver. bias	Differential ver. bias	Bias in the Analysis	Validity bias	Reproducibility bias
DIAGNOdent 2095																
Tagtekin et al. (2008)	x	-	-	-	+	+	?	+	?	-	+	+	+	+	?	+
DIAGNOdent Pen 2190																
Lussi et al. (2006)	x	-	-	-	+	+	-	+	-	-	+	+	+	+	?	-
De Souza et al. (2014)	x	-	+	+	+	+	-	+	+	-	+	+	+	+	?	+
Neuhaus et al. (2015)	x	?	?	-	+	+	?	+	+	-	+	+	+	+	?	+
Bozdemir et al. (2016)	x	-	?	-	+	-	-	+	+	-	+	+	+	+	+	?

Legend: +=Low risk of bias *(Yes*); -=High risk of bias (*Probably No*, *No*); ?=Unclear (*No information, Incomplete reporting, Probably Yes*) x=*Question for in vivo studies*

Table S6f Risk of bias assessment for laser fluorescence measurements of in vivo validation studies on proximal surfaces

Laser Fluorescence measurements of *in vivo* validation studies on proximal surfaces	Signaling questions															
	Selection bias				Index test bias			Reference test bias				Verification bias			Outcome bias	
	Patient selection	Teeth selection	Caries Spectrum	Sample size	Test Criteria	Blinding bias	Calibration bias	Test Criteria	Blinding bias	Calibration bias	Incorporation bias	Partial ver. bias	Differential ver. bias	Bias in the Analysis	Validity bias	Reproducibility bias
DIAGNOdent Pen 2190																
Huth et al. (2010)	-	?	-	+	+	+	?	+	+	?	+	+	-	+	-	+
Kühnisch et al. (2016)	-	+	-	+	+	+	+	+	+	+	+	+	+	+	+	-
Menem et al. (2017)	?	+	+	+	?	-	+	+	-	+	+	+	+	+	+	-
Ozkan et al. (2017)	-	?	-	+	+	-	+	+	-	-	+	+	+	+	+	+

Legend: +=Low risk of bias (*Yes*); -=High risk bias (*Probably No*, *No*); ?=Unclear (*No information, Incomplete reporting, Probably Yes*)

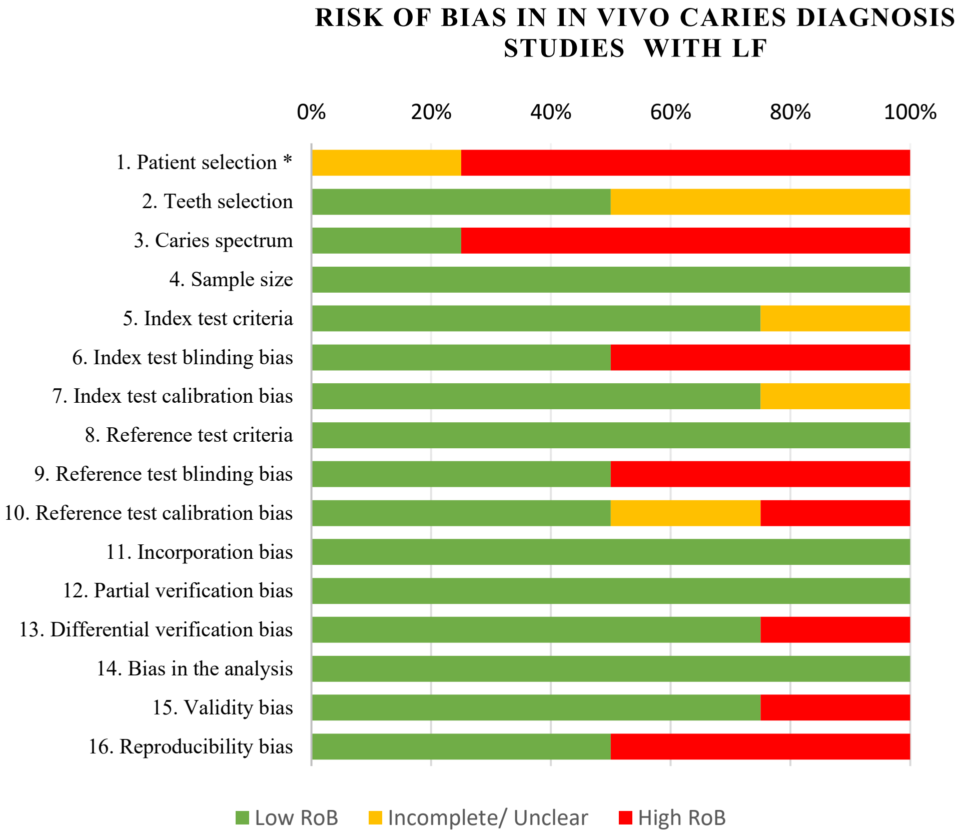

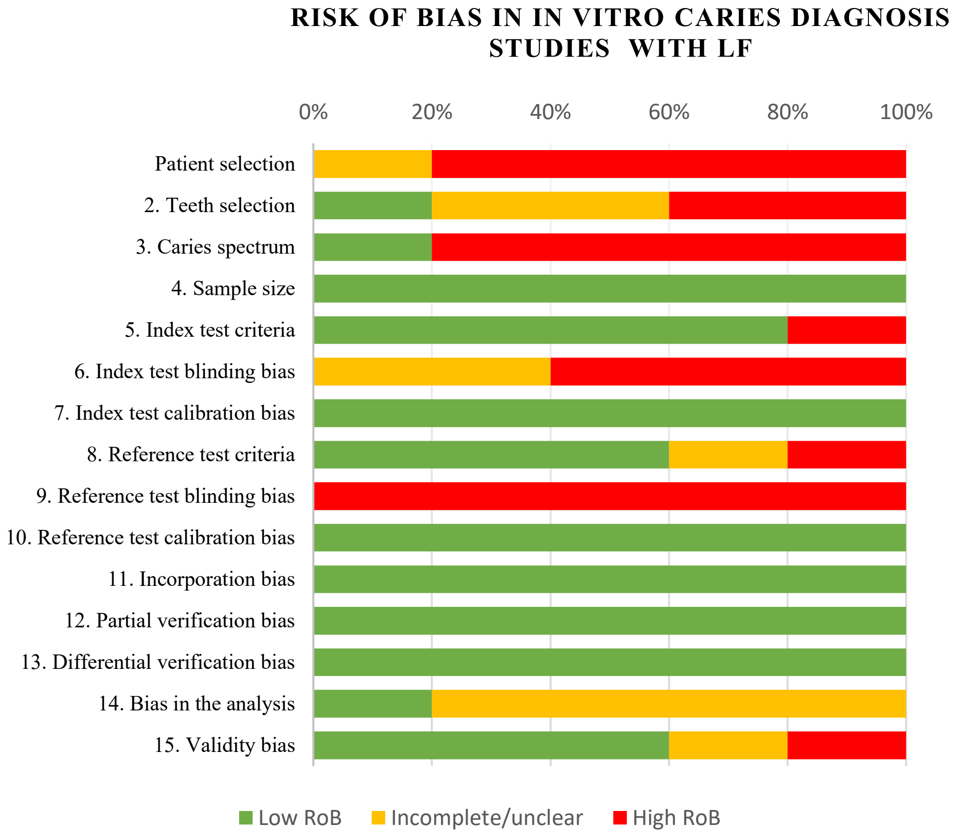

Figure S4 Risk of bias graph for in vitro and in vivo caries diagnostic studies with laser fluorescence: review authors' judgements about each risk of bias item presented as percentages across all included studies

* Item no 1 (Patient selection bias) is only available for clinical diagnostic studies

Table S6g Studies assessed for inclusion in the meta-analysis- laser fluorescence of in vitro validation studies on proximal surfaces

Studies that passed 1st round of selection	ITC	RTC	Sample size and caries distribution				Device		Cut-off	2nd round of selection	Cross-tabulation given	SE	SP	Az	3th round of selection
			Total	S	E	D									
Bozdemir et al. (2016)	4	3	156 surf.	73	44	39	*DIAGNOdent Pen 2190*		Caries detection level	included	no	94/77.1	60.3/75.3	0.88/0.79	included
									Dentin detection level	included	no	79.5/76.9	79.5/76.9	0.84/0.79	included
Neuhaus et al. 2015	2	4	118surf	*38*	*45*	*35*	*DIAGNOdent Pen 2190*		Caries detection level	included	yes	47	78	-	included
									Dentin detection level	included	yes	39	84	-	
De Souza et al. 2014	1	1	102 surf	53	14	35	*DIAGNOdent Pen 2190*		Caries detection level	included	no	80	96	0.93	included
									Dentin detection level	included	no	94	95	0.98	
Tagtekin et al. 2008	3	9	42 surf	-	-	-	*DIAGNOdent 2095*	cone shaped tip	Caries detection level	included	no		24/57	-	excluded
									Dentin detection level	included	no	71/85	-	--	
								broad tip	Caries detection level	included	no	-	24/76		
									Dentin detection level	included	no	48/52	-	-	
Lussi et al. 2006	4	1	150 surf	61	52	37	*DIAGNOdent Pen 2190*	wedge shaped	Caries detection level	included	no	88	92	-	included
									Dentin detection level	included	no	89	82	--	
								tapered wedge shaped	Caries detection level	included	no	87	93	-	
									Dentin detection level	included	no	92	81		

ITC-Index test criteria, RTC- Reference test criteria, S-Sound, E-Caries in enamel, D- Caries in dentin

Table S6h Studies assessed for inclusion in the meta-analysis- laser fluorescence of in vivo validation studies on proximal surfaces

Studies that passed 1st round of selection	ITC	RTC	Sample size and caries distribution				Device	Cut-off	2nd round of selection	Cross-tabulation given	SE	SP	Az	3th round of selection
			Total	S	E	D								
Huth et al. 2010	own	1	117surf	40	34	43	*DIAGNOdent Pen 2190*	Dentine detection level	included	no	60	84	-	included
Kühnisch et al. 2016	1	*	127surf	0	0	127	*DIAGNOdent Pen 2190*	Dentine detection level	included	yes	67	-	0.82	included
Menem et al. 2017	own	3	90surf	30	60		*DIAGNOdent Pen 2190*	Caries detection level	included	yes	92	90	95	included
Ozkan et al. 2017	1	4	161 surf	0	5	156	*DIAGNOdent Pen 2190*	Dentin caries detection level	included	no	60	20	0.40	included

ITC-Index test criteria, RTC- Reference test criteria, S-Sound, E-Caries in enamel, D- Caries in dentin

FOTI

Table S7a Results of Systematic Literature Review for in vitro FOTI validation studies on proximal surfaces

FOTI examination	Study material		Diagnostics	Validation methodology				Validity					
In vitro validation studies on proximal surfaces								Caries detection level			Dentin caries detection level		
	Teeth (N)	Molars/ PM (N)	FOTI Criteria[1]	Hard tissue processing	Caries Staining	Visualisation /magnification	Scoring criteria[2]	SE (%)	SP (%)	Az (ROC)	SE (%)	SP (%)	Az (ROC)
Peers et al. (1993)	240	-/-	4	Slices	-	Microscopy	nr	-	-	-	67	96	-
Schneiderman et al. (1997)	50	14/12	nr	Slices/Groun	SvB[s]/Fuchsin	Microscopy	nr	56	76	-	-	-	-
Astvaldsdottir et al. (2012)	56	-/56	1	Slices	-	Microscopy/M-rad	1	-	-	-	-	-	-
Abogazalah et al. (2019)	30[surf]	-/-	5	-	-	Micro-CT 3	6	91 30[surfaces]	69	0.91	-	-/- -	-

[1]See Table S1d; [2]See Table S1e; nr-not reported

Table S7b Results of Systematic Literature Review for in vivo FOTI validation studies on proximal surfaces

FOTI Examination	Study material			Diagnostics	In vivo validation methodology			Validity					
In vivo validation studies on proximal surfaces								Caries detection level			Dentin caries detection level		
	Patients (N)	Age (years)	Teeth (N)	FOTI Criteria	Histology technique	Validation	Reference method	SE (%)	SP (%)	Az (ROC)	SE (%)	SP (%)	Az (ROC)
Hintze et al. (1998)	53	20-38	338[surfaces]	2	nr	Tooth separation	3	-	-	-	63	93	-
Mialhe et al. (2003)	70	13-15	199[surfaces]	2	nr	Tooth separation	3	-	-	-	-	-	-
Bin-Shuwaish et al. (2008)	21	20-54	51	3	nr	Radiography	nr	-	-	-	100	27	-

nr-not reported

Table S7c Reproducibility/Reliability results of Systematic Literature Review for in vitro FOTI studies on proximal surfaces

FOTI examination	Study material			Diagnostics	Reproducibility/Reliability detection & diagnostic methods		
In vitro validation studies on proximal surfaces	Examiners (N)	Teeth (N)	Molars/PM (N)	FOTI Criteria	Reproducibility/Reliability testing	Intraexaminer	Interexaminer
Peers et al. (1993)	1	240	-/-	4	Cohen's Kappa	0.65	-
Schneiderman et al. (1997)	4	50	14/12	nr	nr	-	-
Astvaldsdottr et al. (2012)	8	56	-/56	1	Cohen's Kappa	0.82	0.62-0.76
Abogazalah et al. (2019)	3	30[surfaces]	-/-	5	ICC	0.85	0.83

nr-not reported

Table S7d Reproducibility/Reliability results of Systematic Literature Review for in vivo FOTI studies on proximal surfaces

FOTI examination	Study material				Diagnostics	Reproducibility/Reliability detection & diagnostic methods		
In vivo validation studies on proximal surfaces	Patients (N)	Examiners (N)	Teeth (N)	Molars/PM (N)	FOTI Criteria	Reproducibility/Reliability testing	Intraexaminer	Interexaminer
Hintze et al. (1998)	53	4	338[surfaces]	163/176	2	Cohen's Kappa	-	0.14-0.26
Mialhe et al. (2003)	70	3	199[surfaces]	-/-	2	Cohen's Kappa	0.83	-
Bin-Shuwaish et al. (2008)	21	1	51		3	nr	-	-

Table S7e Risk of bias assessment for FOTI of in vitro validation studies on proximal surfaces

FOTI of *in vitro* validation studies on proximal surfaces	Signaling questions															
	Selection bias				Index test bias			Reference test bias				Verification bias			Outcome bias	
	Patient selection	Teeth selection	Caries Spectrum	Sample size	Test Criteria	Blinding bias	Calibration bias	Test Criteria	Blinding bias	Calibration bias	Incorporation bias	Partial ver. bias	Differential ver. bias	Bias in the Analysis	Validity bias	Reproducibility bias
Peers et al. (1993)	x	?	?	-	?	+	-	?	+	?	+	?	-	-	?	?
Schneiderman et al. (1997)	x	-	-	-	-	-	?	-	-	-	-	-	+	+	-	-
Astvaldsdottir et al. (2012)	x	-	?	-	+	+	?	+	+	?	+	+	+	+	+	+
Abogazalah et al. (2019)	x	-	-	+	-	+	+	+	+	+	+	+	+	+	+	+

Legend: +=Low risk of bias *(Yes*); -=High risk of bias (*Probably No*, *No*); ?=Unclear (*No information, Incomplete reporting, Probably Yes*) x=*Question for in vivo studies*

Table S7f Risk of bias assessment for FOTI of in vivo validation studies on proximal surfaces

FOTI of *in vivo* validation studies on proximal surfaces	Signaling questions															
	Selection bias				Index test bias			Reference test bias				Verification bias			Outcome bias	
	Patient selection	Teeth selection	Caries Spectrum	Sample size	Test Criteria	Blinding bias	Calibration bias	Test Criteria	Blinding bias	Calibration bias	Incorporation bias	Partial ver. bias	Differential ver. bias	Bias in the Analysis	Validity bias	Reproducibility bias
Hintze et al. (1998)	-	+	-	-	+	-	?	-	-	?	-	+	+	+	?	?
Mialhe et al. (2003)	-	?	-	-	+	+	?	-	-	?	+	+	+	+	?	-
Bin-Shuwaish et al. (2008)	x	?	-	-	?	+	-	+	+	-	+	+	+	+	?	-

Legend: +=Low risk of bias (Yes); -=High risk of bias (Probably No, No); ?=Unclear (No information, Incomplete reporting, Probably Yes)

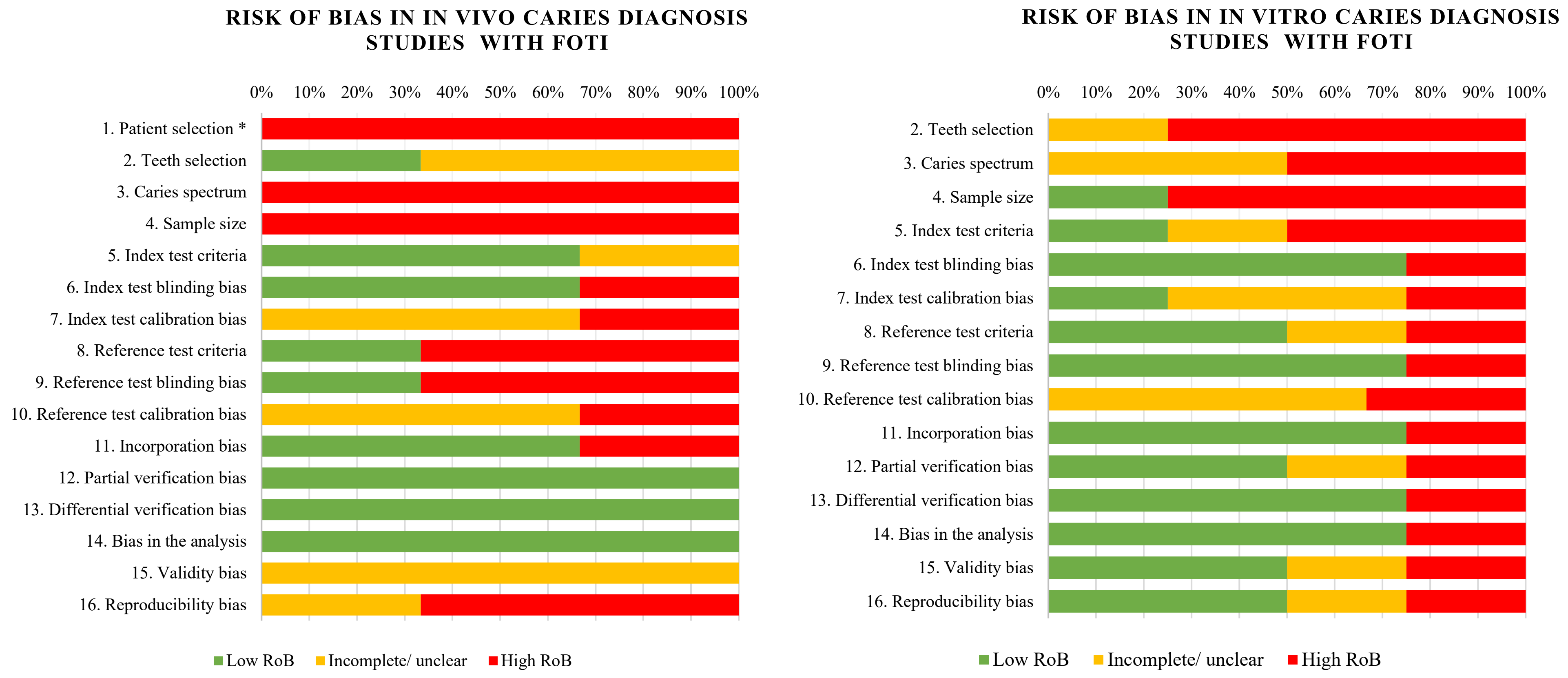

Figure S5 Risk of bias graph for in vivo and in vitro caries diagnostic studies with FOTI: review authors' judgements about each risk of bias item presented as percentages across all included studies

* Item no 1 (Patient selection bias) is only available for clinical diagnostic studies

Meta analytic statistics

Table S8 Forest plots (DOR) for different caries diagnostic methods- in vitro validation studies on proximal surfaces

<table>
<tr><th rowspan="2">Forest Plots</th><th colspan="3">In vitro</th></tr>
<tr><th>Caries detection level</th><th>Dentin detection level</th><th>1/3 dentine detection level</th></tr>
<tr><td>Visual Examination</td><td>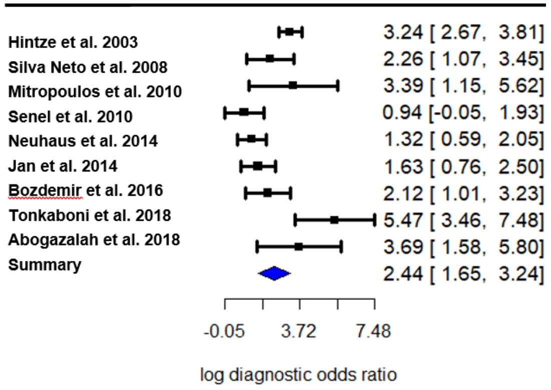
</td><td>No Data Available</td><td>No Data Available</td></tr>
<tr><td>Conventional Bitewing Radiography (D-speed)</td><td>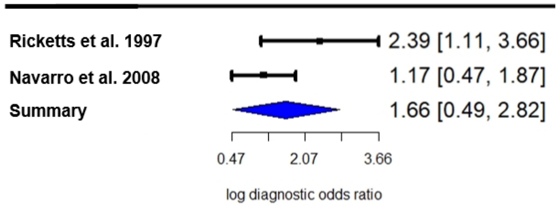
</td><td>No Data Available</td><td>No Data Available</td></tr>
<tr><td>Conventional Bitewing Radiography (E-speed)</td><td>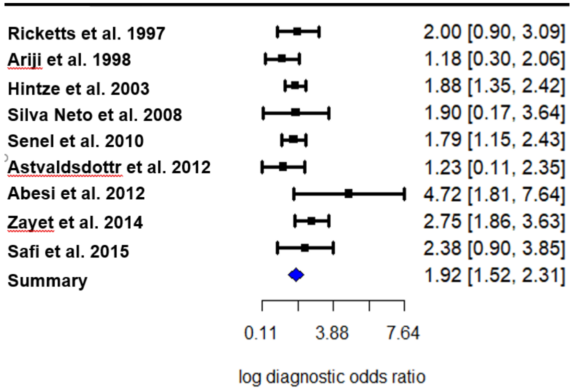
</td><td>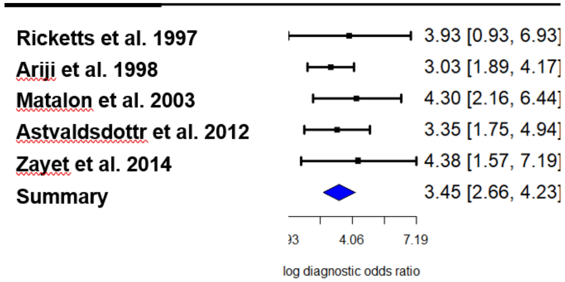
</td><td>No Data Available</td></tr>
</table>

<table>
<tr><th>Forest Plots</th><th colspan="3">In vitro</th></tr>
<tr><th></th><th>Caries detection level</th><th>Dentin detection level</th><th>1/3 dentine detection level</th></tr>
<tr><td>Conventional Bitewing Radiography (F-speed)</td><td>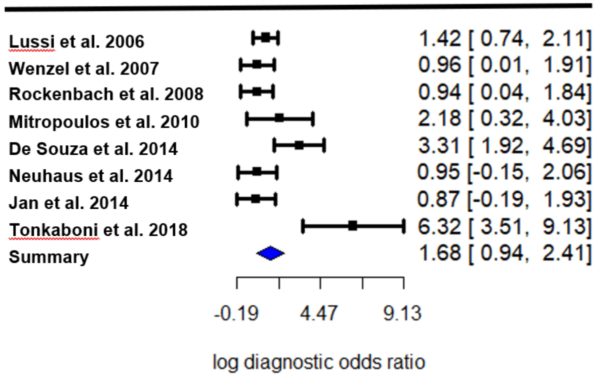
</td><td>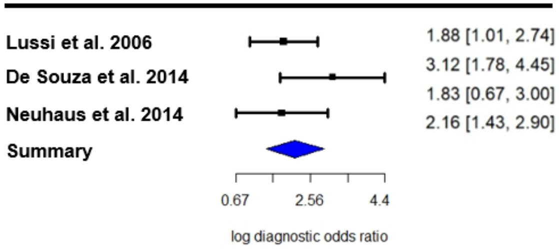
</td><td>No Data Available</td></tr>
<tr><td>Digital Bitewing Radiography (Sensor)</td><td>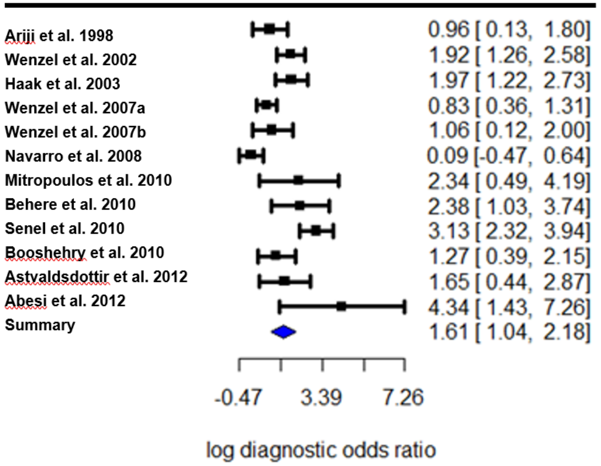
</td><td>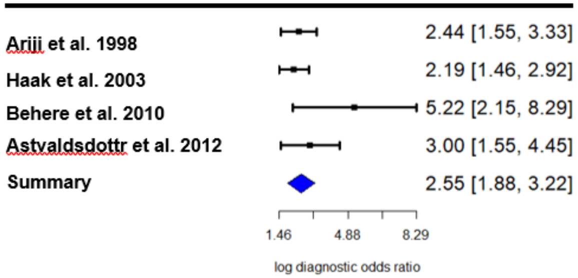
</td><td>No Data Available</td></tr>
</table>

<table>
<tr><th rowspan="2">Forest Plots</th><th colspan="3">In vitro</th></tr>
<tr><th>Caries detection level</th><th>Dentin detection level</th><th>1/3 dentine detection level</th></tr>
<tr><td>Digital Bitewing Radiography (Phosphor plate)</td><td>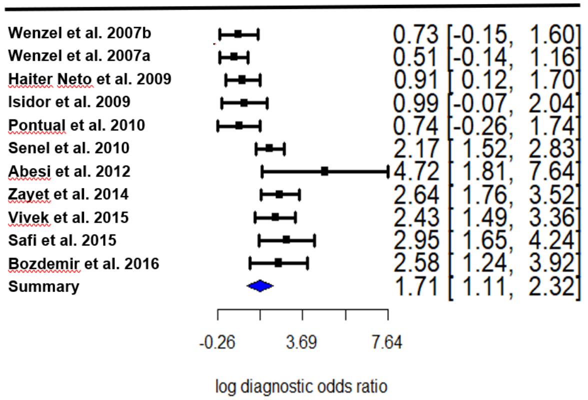
</td><td>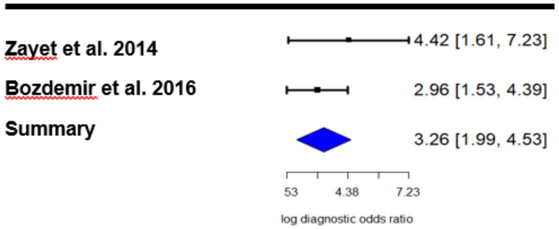
</td><td>No Data Available</td></tr>
<tr><td>Laser Fluorescence Pen 2190</td><td>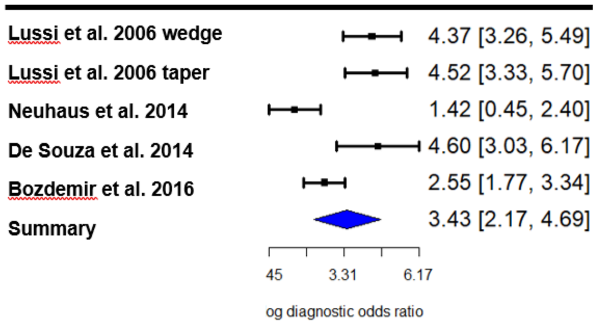
</td><td>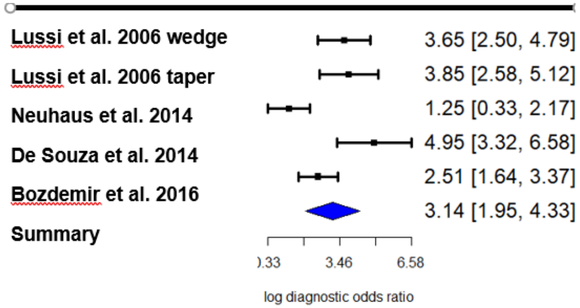
</td><td>No Data Available</td></tr>
</table>

Table S9 Forest plots (DOR) for different caries diagnostic methods- in vivo validation studies on proximal surfaces

Forest Plots	In vivo<br>Caries detection level	 Dentin detection level	 1/3 dentine detection level
Visual Examination	No Data Available	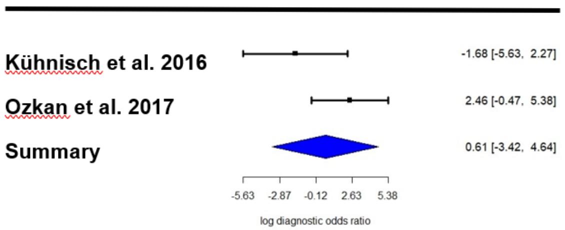	No Data Available
Conventional Bitewing Radiography (D-speed)	No Data Available	No Data Available	No Data Available
Conventional Bitewing Radiography (E-speed)	No Data Available	No Data Available	No Data Available
Conventional Bitewing Radiography (F-speed)	No Data Available	No Data Available	No Data Available
Digital Bitewing Radiography (Sensor)	No Data Available	No Data Available	No Data Available
Digital Bitewing Radiography (Phosphor plate)	No Data Available	No Data Available	No Data Available

Forest Plots		**In vivo**	
	Caries detection level	Dentin detection level	1/3 dentine detection level
Laser Fluorescence Pen 2190	No Data Available	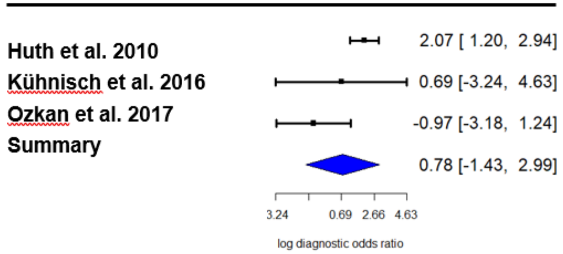	No Data Available

Table S10 SROC for different caries diagnostic methods- in vitro validation studies on proximal surfaces

SROC	In vitro<br>Caries detection level	Dentin detection level	1/3 dentine detection level
Visual Examination	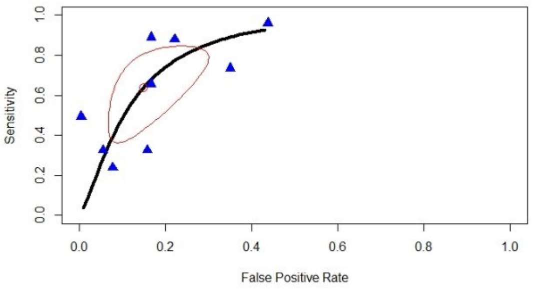<br><br>No. studies= 9<br>AUC= 0.84<br>Pooled Sen= 0.64 (0.42-0.81)<br>Pooled Spe= 0.85 (0.74-0.92)<br>Pooled DOR (REML)= 11.49 (5.19-25.46)<br>Heterogeneity chi-squared= 9.1071, df = 2, p-value = 0.2451<br>Inconsistency (I-squared)= 14.954%<br>Cochran's Q: 9.407 (8 df, p = 0.309)<br>Estimate of between-study variance (Tau-squared)= 1.052	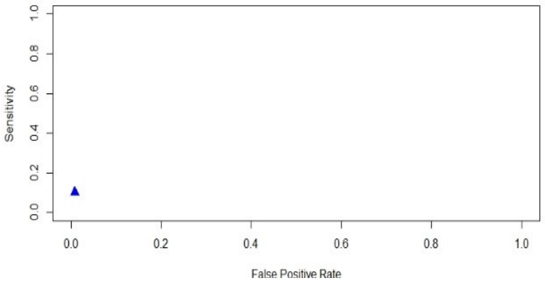<br><br>No. studies= 1<br>AUC= 0.85<br>Pooled Sen= 0.09 (0.04-0.24)<br>Pooled Spe= 0.99 (0.94-0.999)<br>Pooled DOR (REML)= 13.26 (1.44-122.51)<br>Heterogeneity chi-squared= NA<br>Inconsistency (I-squared)= NA<br>Cochran's Q: NA<br>Estimate of between-study variance (Tau-squared)= NA	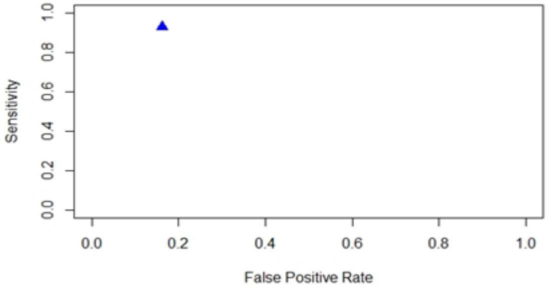<br><br>No. studies= 1<br>AUC= 0.95<br>Pooled Sen= 0.93 (0.77-0.98)<br>Pooled Spe= 0.84 (0.76-0.89)<br>Pooled DOR (REML)= 66.95 (14.70-304.85)<br>Heterogeneity chi-squared= NA<br>Inconsistency (I-squared)= NA<br>Cochran's Q: NA<br>Estimate of between-study variance (Tau-squared)= NA
Conventional Bitewing Radiography (D-speed)	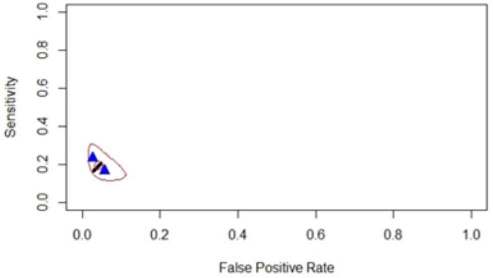<br><br>No. studies= 2<br>AUC= 0.55<br>Pooled Sen= 0.19 (0.13-0.28)<br>Pooled Spe= 0.96 (0.91-0.99)<br>Pooled DOR (REML)= 5.24 (1.64-16.81)<br>Heterogeneity chi-squared= 2.0579, df = 2, p-value < 2.2e-16<br>Inconsistency (I-squared)= 0%<br>Cochran's Q: 1 (1 df, p = 0.317)<br>Estimate of between-study variance (Tau-squared)= 0.460	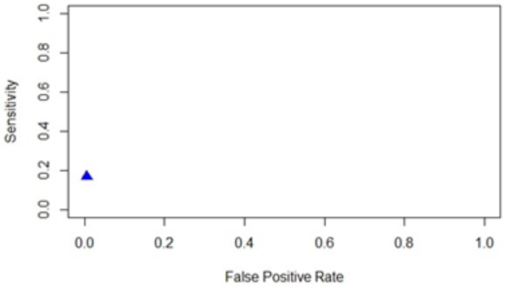<br><br>No. studies= 1<br>AUC= 0.91<br>Pooled Sen= 0.17 (0.07-0.40)<br>Pooled Spe= 0.99 (0.96-0.999)<br>Pooled DOR (REML)= 31 (3.30-291.26)<br>Heterogeneity chi-squared= NA<br>Inconsistency (I-squared)= NA<br>Cochran's Q: NA<br>Estimate of between-study variance (Tau-squared)= NA	No Data Available

SROC	In vitro<br>Caries detection level	 Dentin detection level	 1/3 dentine detection level

Conventional Bitewing Radiography (E-speed)

Caries detection level:

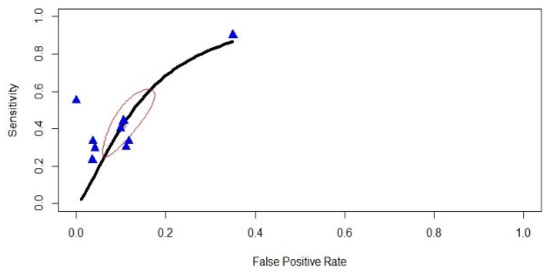

No. studies= 9
AUC= 0.82
Pooled Sen= 0.42 (0.28-0.57)
Pooled Spe= 0.90 (0.84-0.93)
Pooled DOR (REML)= 6.80 (4.58-10.10)
Heterogeneity chi-squared= 8.8409, df = 2, p-value = 0.2643
Inconsistency (I-squared)= 10.157%
Cochran's Q: 8.904 (8 df, p = 0.35)
Estimate of between-study variance (Tau-squared)= 0.106

Dentin detection level:

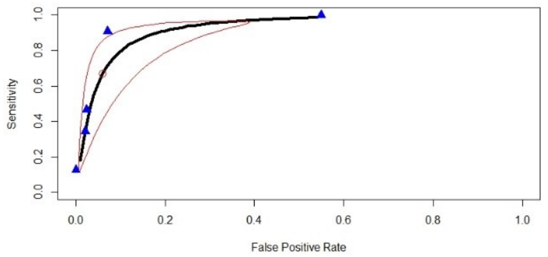

No. studies= 5
AUC= 0.92
Pooled Sen= 0.67 (0.19-0.95)
Pooled Spe= 0.94 (0.71-0.99)
Pooled DOR (REML)= 31.40 (14.31-68.93)
Heterogeneity chi-squared= 4.8359, df = 2, p-value = 0.1842
Inconsistency (I-squared)= 0%
Cochran's Q: 1.662 (4 df, p = 0.798)
Estimate of between-study variance (Tau-squared)= 0.000

1/3 dentine detection level:

No Data Available

Conventional Bitewing Radiography (F-speed)

Caries detection level:

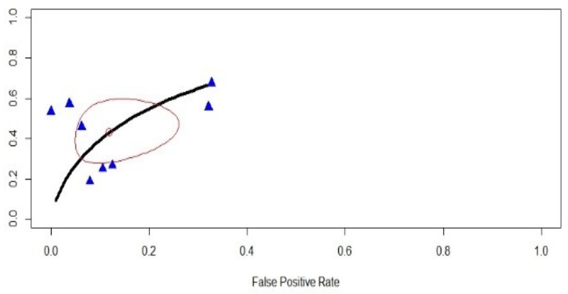

No. studies= 8
AUC= 0.72
Pooled Sen= 0.43 (0.31-0.57)
Pooled Spe= 0.88 (0.74-0.95)
Pooled DOR (REML)= 5.34 (2.55-11.17)
Heterogeneity chi-squared= 7.6708, df = 2, p-value = 0.2632
Inconsistency (I-squared)= 43.716%
Cochran's Q: 12.437 (7 df, p = 0.087)
Estimate of between-study variance (Tau-squared)= 0.722

Dentin detection level:

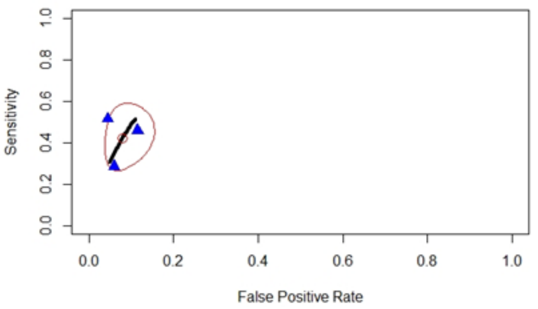

No. studies= 3
AUC= 0.81
Pooled Sen= 0.42 (0.29-0.56)
Pooled Spe= 0.92 (0.86-0.96)
Pooled DOR (REML)= 8.71 (4.19-18.09)
Heterogeneity chi-squared= 2.9074, df = 2, p-value = 0.08817
Inconsistency (I-squared)= 4.628%
Cochran's Q: 2.097 (2 df, p= 0.35)
Estimate of between-study variance (Tau-squared)= 0.109

1/3 dentine detection level:

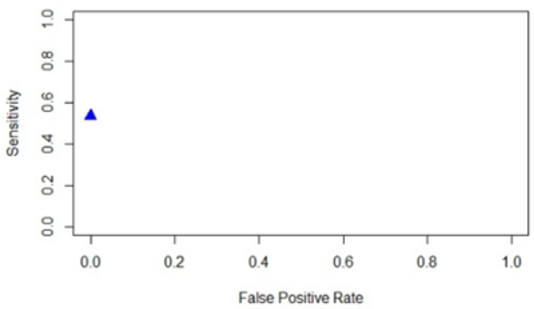

No. studies= 1
AUC= 0.98
Pooled Sen= 0.0.54 (0.36-0.70)
Pooled Spe= 0.996 (0.96-1)
Pooled DOR (REML)= 283.59 (16.05-5010.02)
Heterogeneity chi-squared= NA
Inconsistency (I-squared)= NA
Cochran's Q: NA
Estimate of between-study variance (Tau-squared)= NA

SROC	In vitro<br>Caries detection level	 Dentin detection level	 1/3 dentine detection level
Digital Bitewing Radiography (Sensor)	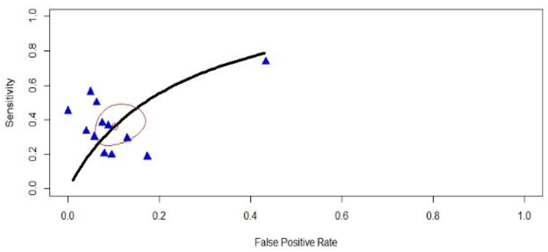<br><br>No. studies= 12<br>AUC= 0.74<br>Pooled Sen= 0.35 (0.26-0.45)<br>Pooled Spe= 0.90 (0.85-0.93)<br>Pooled DOR (REML)= 5.01 (2.83-8.88)<br>Heterogeneity chi-squared= 11.931, df = 2, p-value = 0.2897<br>Inconsistency (I-squared)= 0%<br>Cochran's Q: 10.631 (11 df, p = 0.475)<br>Estimate of between-study variance (Tau-squared)= 0.739	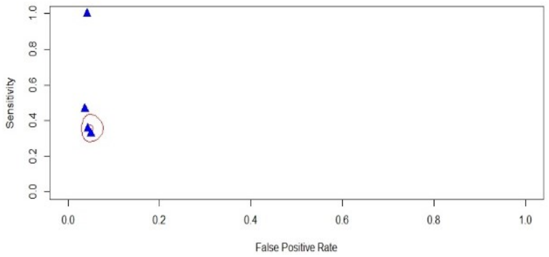<br><br>No. studies= 4<br>AUC= 0.90<br>Pooled Sen= 0.36 (0.30-0.42)<br>Pooled Spe= 0.95 (0.93-0.97)<br>Pooled DOR (REML)= 12.84 (6.56-25.13)<br>Heterogeneity chi-squared= 3.8226, df = 2, p-value = 0.1479<br>Inconsistency (I-squared)= 15.87%<br>Cochran's Q: 3.566 (3 df, p = 0.312)<br>Estimate of between-study variance (Tau-squared)= 0.132	No Data Available
Digital Bitewing Radiography (Phosphor plate)	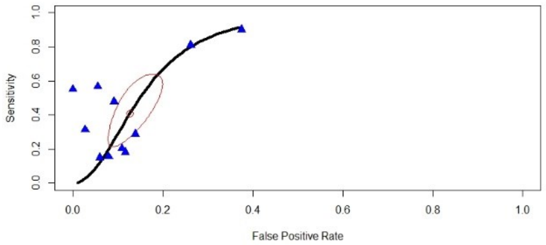<br><br>No. studies= 11<br>AUC= 0.82<br>Pooled Sen= 0.41 (0.25-0.61)<br>Pooled Spe= 0.89 (0.83-0.93)<br>Pooled DOR (REML)= 5.56 (3.04-10.16)<br>Heterogeneity chi-squared= 11.099, df = 2, p-value = 0.269<br>Inconsistency (I-squared)= 13.267%<br>Cochran's Q: 11.53 (10 df, p = 0.318)<br>Estimate of between-study variance (Tau-squared)= 0.745	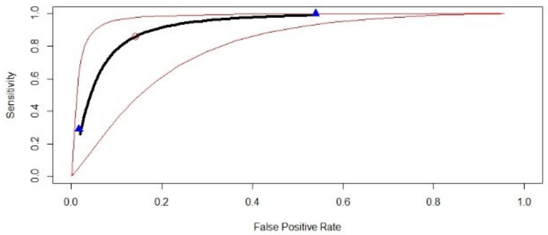<br><br>No. studies= 2<br>AUC= 0.92<br>Pooled Sen= 0.86 (0.03-0.99)<br>Pooled Spe= 0.86 (0.11-0.99)<br>Pooled DOR (REML)= 26.06 (7.29-93.21)<br>Heterogeneity chi-squared= 2.0136, df = 2, p-value < 2.2e-16<br>Inconsistency (I-squared)= 0%<br>Cochran's Q: 0.82 (1 df, p = 0.365)<br>Estimate of between-study variance (Tau-squared)= 0.00	No Data Available

<table>
<tr><th rowspan="2">SROC</th><th colspan="3">In vitro</th></tr>
<tr><th>Caries detection level</th><th>Dentin detection level</th><th>1/3 dentine detection level</th></tr>
<tr><td>Laser Fluorescence</td><td>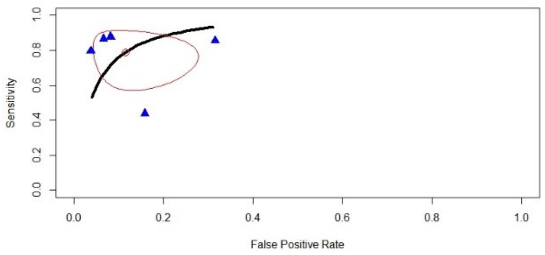

No. studies= 5
AUC= 0.91
Pooled Sen= 0.79 (0.62-0.90)
Pooled Spe= 0.89 (0.76-0.95)
Pooled DOR (REML)= 30.79 (8.74-108.51)
Heterogeneity chi-squared= 5.6875, df = 2, p-value = 0.1278
Inconsistency (I-squared)= 0.458%
Cochran's Q: 4.018 (4 df, p = 0.404)
Estimate of between-study variance (Tau-squared)= 1.729</td><td>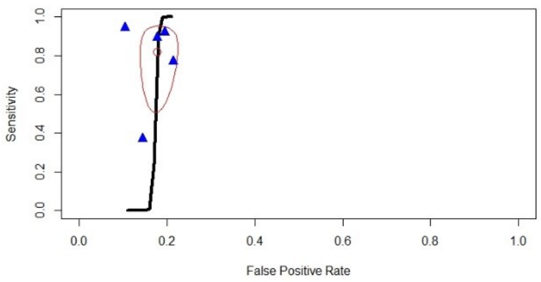

No. studies= 5
AUC= 0.83
Pooled Sen= 0.82 (0.58-0.94)
Pooled Spe= 0.81 (0.78-0.85)
Pooled DOR (REML)= 23.09 (7.01-76.04)
Heterogeneity chi-squared= 6.7615, df = 2, p-value = 0.0799
Inconsistency (I-squared)= 5.414%
Cochran's Q: 4.229 (4 df, p = 0.376)
Estimate of between-study variance (Tau-squared)= 1.491</td><td>No Data Available</td></tr>
</table>

Table S11 SROC for different caries diagnostic methods- in vivo validation studies on proximal surfaces

SROC	In vivo<br>Caries detection level	Dentin detection level	1/3 dentine detection level
Visual Examination	No Data Available	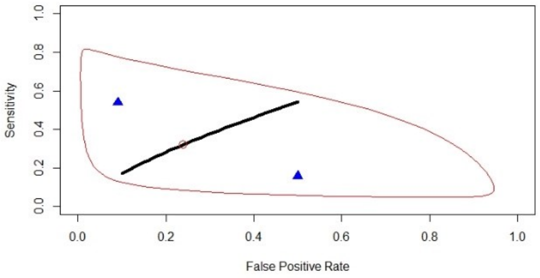<br>No. studies= 2<br>AUC= 0.53<br>Pooled Sen= 0.32 (0.07-0.74)<br>Pooled Spe= 0.76 (0.11-0.99)<br>Pooled DOR (REML)= 1.84 (0.03-103.47)<br>Heterogeneity chi-squared= 2.9529, df = 2, p-value < 2.2e-16<br>Inconsistency (I-squared)= 0%<br>Cochran's Q: 1 (1 df, p = 0.317)<br>Estimate of between-study variance (Tau-squared)= 5.402	No Data Available
Conventional Bitewing Radiography (D-speed)	No Data Available	No Data Available	No Data Available

SROC	Caries detection level	**In vivo**<br>Dentin detection level	1/3 dentine detection level
Conventional Bitewing Radiography (E-speed)	No Data Available	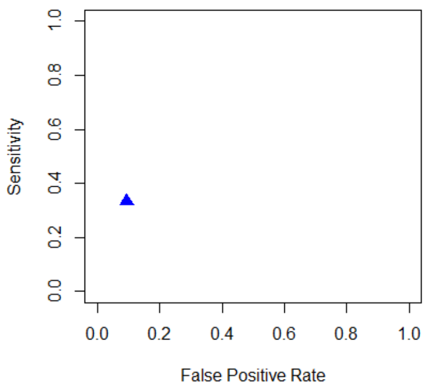<br>No. studies= 1<br>AUC= 0.74<br>Pooled Sen= 0.35 (0.19-0.51)<br>Pooled Spe= 0.91 (0.80-0.96)<br>Pooled DOR (REML)= 4.8 (1.48-15.48)<br>Heterogeneity chi-squared= NA<br>Inconsistency (I-squared)= NA<br>Cochran's Q: NA<br>Estimate of between-study variance (Tau-squared)= NA	No Data Available
Conventional Bitewing Radiography (F-speed)	No Data Available	No Data Available	No Data Available

SROC	Caries detection level	**In vivo**<br>Dentin detection level	1/3 dentine detection level
Digital Bitewing Radiography (Sensor)	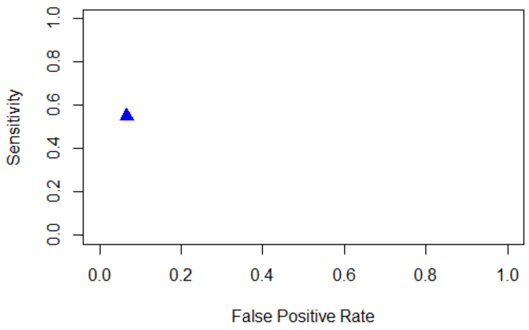<br>No. studies= 1<br>AUC= 0.82<br>Pooled Sen= 0.55 (0.42-0.67)<br>Pooled Spe= 0.93 (0.77-0.98)<br>Pooled DOR (REML)= 17.11 (3.73-78.39)<br>Heterogeneity chi-squared= NA<br>Inconsistency (I-squared)= NA<br>Cochran's Q: NA<br>Estimate of between-study variance (Tau-squared)= NA	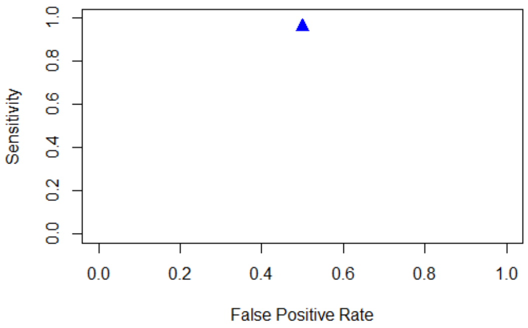<br>No. studies= 1<br>AUC= 0.89<br>Pooled Sen= 0.96 (0.91-0.98)<br>Pooled Spe= 0.50 (0.02-0.98)<br>Pooled DOR (REML)= 24.4 (0.44-1359.98)<br>Heterogeneity chi-squared= NA<br>Inconsistency (I-squared)= NA<br>Cochran's Q: NA<br>Estimate of between-study variance (Tau-squared)= NA	No Data Available
Digital Bitewing Radiography (Phosphor plate)	No Data Available	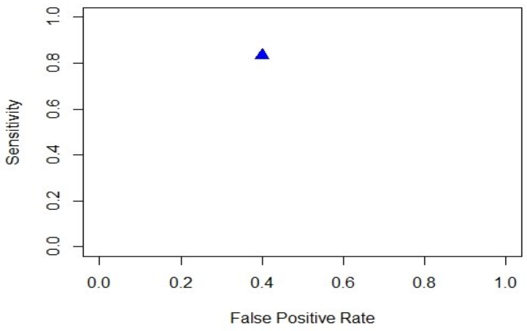<br>No. studies= 1<br>AUC= 0.79<br>Pooled Sen= 0.83 (0.77-0.88)<br>Pooled Spe= 0.60 (0.20-0.90)<br>Pooled DOR (REML)= 7.5 (1.19-47.13)<br>Heterogeneity chi-squared= NA<br>Inconsistency (I-squared)= NA<br>Cochran's Q: NA<br>Estimate of between-study variance (Tau-squared)= NA	No Data Available

SROC	**In vivo**<br>Caries detection level	Dentin detection level	1/3 dentine detection level
Laser Fluorescence Pen 2190	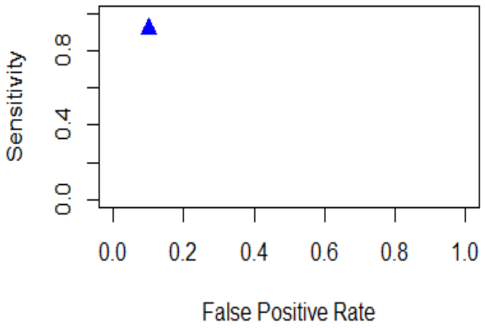<br>No. studies= 1<br>AUC= 0.96<br>Pooled Sen= 0.92 (0.82-0.96)<br>Pooled Spe= 0.90 (0.73-0.97)<br>Pooled DOR (REML)= 99 (22.01-445.30)<br>Heterogeneity chi-squared= NA<br>Inconsistency (I-squared)= NA<br>Cochran's Q: NA<br>Estimate of between-study variance (Tau-squared)= NA	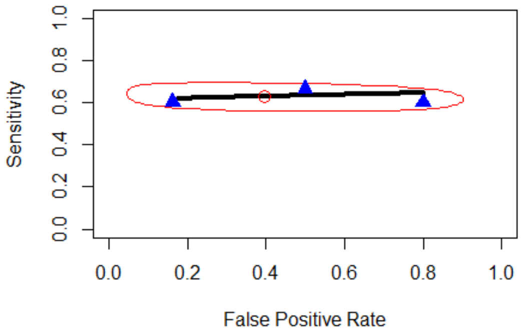<br>No. studies= 3<br>AUC= 0.63<br>Pooled Sen= 0.63 (0.58-0.68)<br>Pooled Spe= 0.60 (0.15-0.93)<br>Pooled DOR (REML)= 2.18 (0.24-19.95)<br>Heterogeneity chi-squared= 3.4273, df = 2, p-value < 2.2e-16<br>Inconsistency (I-squared)= 0%<br>Cochran's Q: 0.521 (1 df, p = 0.471)<br>Estimate of between-study variance (Tau-squared)= 0.000	No Data Available

References

Abdinian M, Razavi SM, Faghihian R, Samety AA, Faghihian E (2015). Accuracy of Digital Bitewing Radiography versus Different Views of Digital Panoramic Radiography for Detection of Proximal Caries. J Dent (Tehran); 12(4):290-7.

Abesi F, Mirshekar A, Moudi E, Seyedmajidi M, Haghanifar S, Haghighat N, Bijani A (2012). Diagnostic accuracy of digital and conventional radiography in the detection of non-cavitated approximal dental caries. Iran J Radiol; 9(1):17-21.

Abogazalah N, Eckert GJ, Ando M (2019). In vitro visual and visible light transillumination methods for detection of natural non-cavitated approximal caries. Clin Oral Investig; 23(3):1287-1294.

Abreu Junior M, Tyndall DA, Platin E, Ludlow JB, Phillips C (1999). Two- and three-dimensional imaging modalities for the detection of caries. A comparison between film, digital radiography and tuned aperture computed tomography (TACT). Dentomaxillofac Radiol; 28(3):152-7.

Abreu M, Jr., Mol A, Ludlow JB (2001). Performance of RVGui sensor and Kodak Ektaspeed Plus film for proximal caries detection. Oral Surg Oral Med Oral Pathol Oral Radiol Endod; 91(3):381-5.

Adibi S, Amrollahi A, Dehghani Nazhvani A, Movahhedian N (2018). Assessing the Accuracy of Caries Diagnosis in Bitewing Radiographs Using Different Reproduction Media. J Dent (Shiraz); 19(3):174-180.

Akarslan ZZ, Akdevelioglu M, Gungor K, Erten H (2008). A comparison of the diagnostic accuracy of bitewing, periapical, unfiltered and filtered digital panoramic images for approximal caries detection in posterior teeth. Dentomaxillofac Radiol; 37(8):458-63.

Akbari M, Zarch HH, Movagharipour F, Ahrari F (2013). A pilot study of a modified radiographic technique for detecting early proximal cavities. Caries Res; 47(6):612-6.

Akdeniz BG, Grondahl HG, Magnusson B (2006). Accuracy of proximal caries depth measurements: Comparison between limited cone beam computed tomography, storage phosphor and film radiography. Caries Research; 40(3):202-207.

Akkaya N, Kansu O, Kansu H, Cagirankaya LB, Arslan U (2006). Comparing the accuracy of panoramic and intraoral radiography in the diagnosis of proximal caries. Dentomaxillofacial Radiology; 35(3):170-174.

Aleksejuniene J, Tranaeus S, Skudutyte-Rysstad R (2006). DIAGNOdent - An adjunctive diagnostic method for caries diagnosis in epidemiology. Community Dental Health; 23(4):217-221.

Alkurt MT, Peker I, Bala O, Altunkaynak B (2007). In vitro comparison of four different dental x-ray films and direct digital radiography for proximal caries detection. Operative Dentistry; 32(5):504-509.

Ardakani FE, Davari A, Goodarzipour D, Goodarzipour K, Fallahzadeh H (2004). Evaluation of the diagnostic advantage of intraoral D and E film for detecting interproximal caries. Journal of Contemporary Dental Practice; 5(4):58-70.

Ariji Y, Takahashi JI, Matsui O, Okano T, Naitoh M, Yuasa H, Iida H, Hasegawa J, Senda A, Ariji E (1998). In vitro comparison of subjective image quality of the Pana Digital intraoral x-ray imaging system and conventional intraoral radiography in caries detection. Oral Radiology; 14(2):75-83.

Astvaldsdottir A, Ahlund K, Holbrook WP, de Verdier B, Tranaeus S (2012). Approximal Caries Detection by DIFOTI: In Vitro Comparison of Diagnostic Accuracy/Efficacy with Film and Digital Radiography. Int J Dent; 2012:326401.

Baltacioglu IH, Orhan K (2017). Comparison of diagnostic methods for early interproximal caries detection with near-infrared light transillumination: an in vivo study. BMC oral health; 17(1):130.

Barbosa VL, Gonzaga AK, Pontual AA, Bento PM, Ramos-Perez FM, Filgueira PT, Melo DP (2015). The influence of display modalities on proximal caries detection and treatment decision. Acta Odontol Latinoam; 28(2):95-102.

Behere R, Lele S (2011). Reliability of Logicon caries detector in the detection and depth assessment of dental caries: An in-vitro study. Indian Journal of Dental Research; 22(2):362.

Belem MD, Ambrosano GM, Tabchoury CP, Ferreira-Santos RI, Haiter-Neto F (2013). Performance of digital radiography with enhancement filters for the diagnosis of proximal caries. Braz Oral Res; 27(3):245-51.

Berg SC, Stahl JM, Lien W, Slack CM, Vandewalle KS (2018). A clinical study comparing digital radiography and near-infrared transillumination in caries detection. J Esthet Restor Dent; 30(1):39-44.

Berkhout WER, Verheij JGC, Syriopoulos K, Li G, Sanderink GCH, Van Der Stelt PF (2007). Detection of proximal caries with high-resolution and standard resolution digital radiographic systems. Dentomaxillofacial Radiology; 36(4):204-210.

Bin-Shuwaish M, Yaman P, Dennison J, Neiva G (2008). The correlation of DIFOTI to clinical and radiographic images in Class II carious lesions. Journal of the American Dental Association (1939); 139(10):1374-1381.

Bottenberg P, Jacquet W, Stachniss V, Wellnitz J, Schulte AG (2011). Detection of cavitated or non-cavitated approximal enamel caries lesions using CMOS and CCD digital X-ray sensors and conventional D and F-speed films at different exposure conditions. American Journal of Dentistry; 24(2):74-78.

Bozdemir E, Aktan AM, Ozsevik A, Sirin Kararslan E, Ciftci ME, Cebe MA (2016). Comparison of different caries detectors for approximal caries detection. J Dent Sci; 11(3):293-298.

Caliskan Yanikoglu F, Ozturk F, Hayran O, Analoui M, Stookey GK (2000). Detection of natural white spot caries lesions by an ultrasonic system. Caries Res; 34(3):225-32.

Castro VM, Katz JO, Hardman PK, Glaros AG, Spencer P (2007). In vitro comparison of conventional film and direct digital imaging in the detection of approximal caries. Dentomaxillofacial Radiology; 36(3):138-142.

Cheng JG, Zhang ZL, Wang XY, Zhang ZY, Ma XC, Li G (2012). Detection accuracy of proximal caries by phosphor plate and cone-beam computerized tomography images scanned with different resolutions. Clinical Oral Investigations; 16(4):1015-1021.

Choksi SK, Brady JM, Dang DH, Rao MS (1994). Detecting approximal dental caries with transillumination: a clinical evaluation. J Am Dent Assoc; 125(8):1098-102.

Clifton TL, Tyndall DA, Ludlow JB (1998). Extraoral radiographic imaging of primary caries. Dentomaxillofac Radiol; 27(4):193-8.

Crombie K, Parker ME, Nortje CJ, Sanderink GC (2009). Comparing the performance of storage phosphor plate and Insight film images for the detection of proximal caries depth. SADJ : journal of the South African Dental Association = tydskrif van die Suid-Afrikaanse Tandheelkundige Vereniging; 64(10):452, 454-456, 458-459.

da Silva Neto JM, dos Santos RL, Sampaio MC, Sampaio FC, Passos IA (2008). Radiographic diagnosis of incipient proximal caries: an ex-vivo study. Brazilian dental journal; 19(2):97-102.

Dashpuntsag O, Yoshida M, Kasai R, Maeda N, Hosoki H, Honda E (2017). Numerical Evaluation of Image Contrast for Thicker and Thinner Objects among Current Intraoral Digital Imaging Systems. Biomed Res Int; 2017:5215413.

De Araujo EAF, De Melo Castilho JC, Medici Filho E, De Moraes MEL (2005). Comparison of direct digital and conventional imaging with Ekta Speed Plus and INSIGHT films for the detection of approximal caries. American Journal of Dentistry; 18(4):241-244.

De Araujo FB, Rosito DB, Toigo E, dos Santos CK (1992). Diagnosis of approximal caries: radiographic versus clinical examination using tooth separation. Am J Dent; 5(5):245-8.

de Melo SDP, Cruz AD, Melo SLS, Farias JFG, Haiter-Neto F, de Almeida SM (2015). Effect of different tube potential settings on caries detection using psp plate and conventional film. Journal of Clinical and Diagnostic Research; 9(4):ZC58-ZC61.

de Souza JF, Diniz MB, Boldieri T, Rodrigues JA, Lussi A, de Cassia Loiola Cordeiro R (2014). In vitro performance of a pen-type laser fluorescence device and bitewing radiographs for approximal caries detection in permanent and primary teeth. Indian J Dent Res; 25(6):702-10.

de Vries HC, Ruiken HM, Konig KG, van 't Hof MA (1990). Radiographic versus clinical diagnosis of approximal carious lesions. Caries Res; 24(5):364-70.
Dehghani M, Barzegari R, Tabatabai H, Ghanea S (2017). Diagnostic Value of Conventional and Digital Radiography for Detection of Cavitated and Non-Cavitated Proximal Caries. J Dent (Tehran); 14(1):21-30.
dos Anjos Pontual A, de Melo DP, Pontual ML, de Almeida SM, Haiter-Neto F (2013). Comparative study of a wireless digital system and 2 PSP digital systems on proximal caries detection and pixel values. Gen Dent; 61(6):56-60.
Dove SB, McDavid WD (1992). A comparison of conventional intra-oral radiography and computer imaging techniques for the detection of proximal surface dental caries. Dentomaxillofac Radiol; 21(3):127-34.
Downer MC, Kay EJ (1996). Restorative treatment decisions from bitewing radiographs--performance of dental epidemiologists and general dental practitioners. Community Dent Oral Epidemiol; 24(2):101-5.
Eggertsson H, Analoui M, van der Veen M, Gonzalez-Cabezas C, Eckert G, Stookey G (1999). Detection of early interproximal caries in vitro using laser fluorescence, dye-enhanced laser fluorescence and direct visual examination. Caries Res; 33(3):227-33.
Ekstrand KR, Luna LE, Promisiero L, Cortes A, Cuevas S, Reyes JF, Torres CE, Martignon S (2011). The reliability and accuracy of two methods for proximal caries detection and depth on directly visible proximal surfaces: An in vitro study. Caries Research; 45(2):93-99.
Erten H, Akarslan ZZ, Topuz O (2005). The efficiency of three different films and radiovisiography in detecting approximal carious lesions. Quintessence Int; 36(1):65-70.
Espelid I, Tveit AB (1986). Clinical and radiographic assessment of approximal carious lesions. Acta Odontol Scand; 44(1):31-7.
Espelid I, Tveit AB (2001). A comparison of radiographic occlusal and approximal caries diagnoses made by 240 dentists. Acta Odontol Scand; 59(5):285-9.
Faghihian R, Faghihian H (2015). Proximal caries detection accuracy using intra oral digital bitewing radiography and conventional intra oral bitewing radiography with film. [Persian]. Journal of Mazandaran University of Medical Sciences; 25(125):131-134.
Falahzadeh F, Tayyebi A, Tofangchiha M, Modirfalah H, Safarzadeh Khosroshahi S (2013). Agreement of bitewing and digital panoramic radiographies in the detection of proximal caries. [Persian]. Journal of Kerman University of Medical Sciences; 20(4):343-353.
Ferreira RI, Haiter-Neto F, Tabchoury CPM, de Paiva GAN, Boscolo FN (2006). Assessment of enamel demineralization using conventional digital, and digitized radiography. Brazilian Oral Research; 20(2):114-119.
Forner-Navarro L, Puy Ma CL, Godoy FG (2008). Diagnostic performance of radiovisiography in combination with a diagnosis assisting program versus conventional radiography and radiovisiography in basic mode and with magnification. Medicina Oral, Patologia Oral y Cirugia Bucal; 13(4):261-265.
Galcera Civera V, Almerich Silla JM, Montiel Company JM, Forner Navarro L (2007). Clinical and radiographic diagnosis of approximal and occlusal dental caries in a low risk population. Medicina oral, patologia oral y cirugia bucal; 12(3):E252-257.
Ghoncheh Z, Kheirandish Y, Kaviani H, Kharazi Fard MJ, Sadeghi K (2017). Diagnosis of Approximal Caries after Delayed Scanning of Photostimulable Phosphor Plates. J Dent (Shiraz); 18(3):201-206.
Gray BM, Mol A, Zandona A, Tyndall D (2017). The effect of image enhancements and dual observers on proximal caries detection. Oral Surg Oral Med Oral Pathol Oral Radiol; 123(4):e133-e139.
Gungor K, Erten H, Akarslan ZZ, Celik I, Semiz M (2005). Approximal carious lesion depth assessment with insight and ultraspeed films. Oper Dent; 30(1):58-62.
Haak R, Wicht MJ, Noack MJ (2001). Conventional, digital and contrast-enhanced bitewing radiographs in the decision to restore approximal carious lesions. Caries Res; 35(3):193-9.
Haak R, Wicht MJ, Hellmich M, Gossmann A, Noack MJ (2002). The validity of proximal caries detection using magnifying visual aids. Caries Res; 36(4):249-55.
Haak R, Wicht MJ, Nowak G, Hellmich M (2003). Influence of displayed image size on radiographic detection of approximal caries. Dentomaxillofac Radiol; 32(4):242-6.
Haak R, Wicht MJ (2005). Grey-scale reversed radiographic display in the detection of approximal caries. J Dent; 33(1):65-71.

Haiter-Neto F, dos Anjos Pontual A, Frydenberg M, Wenzel A (2007). A comparison of older and newer versions of intraoral digital radiography systems: diagnosing noncavitated proximal carious lesions. J Am Dent Assoc; 138(10):1353-9; quiz 1382-3.

Haiter-Neto F, Wenzel A, Gotfredsen E (2008). Diagnostic accuracy of cone beam computed tomography scans compared with intraoral image modalities for detection of caries lesions. Dentomaxillofacial Radiology; 37(1):18-22.

Haiter-Neto F, Casanova MS, Frydenberg M, Wenzel A (2009). Task-specific enhancement filters in storage phosphor images from the Vistascan system for detection of proximal caries lesions of known size. Oral Surg Oral Med Oral Pathol Oral Radiol Endod; 107(1):116-21.

Heaven TJ, Weems RA, Firestone AR (1994). The use of a computer-based image analysis program for the diagnosis of approximal caries from bitewing radiographs. Caries Res; 28(1):55-8.

Hellen-Halme K, Nilsson M, Petersson A (2009). Effect of monitors on approximal caries detection in digital radiographs-standard versus precalibrated DICOM part 14 displays: An in vitro study. Oral Surgery, Oral Medicine, Oral Pathology, Oral Radiology and Endodontology; 107(5):716-720.

Hellen-Halme K, Petersson GH (2010). Influence of education level and experience on detection of approximal caries in digital dental radiographs. An in vitro study. Swedish Dental Journal; 34(2):63-69.

Hellen-Halme K, Lith A (2013). Carious lesions: diagnostic accuracy using pre-calibrated monitor in various ambient light levels: an in vitro study. Dentomaxillofac Radiol; 42(8):20130071.

Hellén-Halme K, Petersson A, Warfvinge G, Nilsson M (2008). Effect of ambient light and monitor brightness and contrast settings on the detection of approximal caries in digital radiographs: an in vitro study. Dentomaxillofac Radiol; 37(7):380-4.

Hintze H, Wenzel A, Jones C (1994). In vitro comparison of D- and E-speed film radiography, RVG, and visualix digital radiography for the detection of enamel approximal and dentinal occlusal caries lesions. Caries Res; 28(5):363-7.

Hintze H, Christoffersen L, Wenzel A (1996a). In vitro comparison of Kodak Ultra-speed, Ektaspeed, and Ektaspeed Plus, and Agfa M2 Comfort dental x-ray films for the detection of caries. Oral Surg Oral Med Oral Pathol Oral Radiol Endod; 81(2):240-4.

Hintze H, Wenze A (1996b). Clinical and laboratory radiographic caries diagnosis. A study of the same teeth. Dentomaxillofac Radiol; 25(3):115-8.

Hintze H, Wenzel A, Danielsen B, Nyvad B (1998). Reliability of visual examination, fibre-optic transillumination, and bite-wing radiography, and reproducibility of direct visual examination following tooth separation for the identification of cavitated carious lesions in contacting approximal surfaces. Caries Res; 32(3):204-9.

Hintze H, Wenzel A, Frydenberg M (2002a). Accuracy of caries detection with four storage phosphor systems and E-speed radiographs. Dentomaxillofac Radiol; 31(3):170-5.

Hintze H, Wenzel A (2002b). Influence of the validation method on diagnostic accuracy for caries. A comparison of six digital and two conventional radiographic systems. Dentomaxillofac Radiol; 31(1):44-9.

Hintze H, Wenzel A (2003). Diagnostic outcome of methods frequently used for caries validation. A comparison of clinical examination, radiography and histology following hemisectioning and serial tooth sectioning. Caries Res; 37(2):115-24.

Hintze H (2006). Diagnostic accuracy of two software modalities for detection of caries lesions in digital radiographs from four dental systems. Dentomaxillofacial Radiology; 35(2):78-82.

Holtzman J, Osann K, Potdar S, Duong S, Ahn YC, Chen Z, Wilder-Smith P (2011). OCT versus current clinical standards for early-stage caries detection. Lasers in Surgery and Medicine; 23):972.

Huth KC, Lussi A, Gygax M, Thum M, Crispin A, Paschos E, Hickel R, Neuhaus KW (2010). In vivo performance of a laser fluorescence device for the approximal detection of caries in permanent molars. Journal of Dentistry; 38(12):1019-1026.

Huysmans MC, Hintze H, Wenzel A (1997). Effect of exposure time on in vitro caries diagnosis using the Digora system. Eur J Oral Sci; 105(1):15-20.

Isidor S, Faaborg-Andersen M, Hintze H, Kirkevang LL, Frydenberg M, Haiter-Neto F, Wenzel A (2009). Effect of monitor display on detection of approximal caries lesions in digital radiographs. Dentomaxillofacial Radiology; 38(8):537-541.

Jablonski-Momeni A, Jablonski B, Lippe N (2017). Clinical performance of the near-infrared imaging system VistaCam iX Proxi for detection of approximal enamel lesions. BDJ Open; 3:17012.

Jacobsen JH, Hansen B, Wenzel A, Hintze H (2004). Relationship between histological and radiographic caries lesion depth measured in images from four digital radiography systems. Caries Res; 38(1):34-8.

Jan J, Wan Bakar WZ, Mathews SM, Okoye LO, Ehler BR, Louden C, Amaechi BT (2016). Proximal caries lesion detection using the Canary Caries Detection System: an in vitro study. J Investig Clin Dent; 7(4):383-390.

Janhom A, van Ginkel FC, van Amerongen JP, van der Stelt PF (2001). Scanning resolution and the detection of approximal caries. Dentomaxillofac Radiol; 30(3):166-71.

Jessee SA, Makins SR, Bretz WA (1999). Accuracy of proximal caries depth determination using two intraoral film speeds. Gen Dent; 47(1):88-93.

Kajan ZD, Tayefeh Davalloo R, Tavangar M, Valizade F (2015). The effects of noise reduction, sharpening, enhancement, and image magnification on diagnostic accuracy of a photostimulable phosphor system in the detection of non-cavitated approximal dental caries. Imaging Sci Dent; 45(2):81-7.

Kalathingal SM, Mol A, Tyndall DA, Caplan DJ, Hill C (2007). In vitro assessment of cone beam local computed tomography for proximal caries detection. Oral Surgery, Oral Medicine, Oral Pathology, Oral Radiology and Endodontology; 104(5):699-704.

Kamburoglu K, Kolsuz E, Murat S, Yuksel S, Ozen T (2012). Proximal caries detection accuracy using intraoral bitewing radiography, extraoral bitewing radiography and panoramic radiography. Dentomaxillofacial Radiology; 41(6):450-459.

Kay EJ, Knill-Jones R (1992). Variation in restorative treatment decisions: application of Receiver Operating Characteristic curve (ROC) analysis. Community Dent Oral Epidemiol; 20(3):113-7.

Kayipmaz S, Sezgin Ö S, Saricaoğlu ST, Çan G (2011). An in vitro comparison of diagnostic abilities of conventional radiography, storage phosphor, and cone beam computed tomography to determine occlusal and approximal caries. Eur J Radiol; 80(2):478-82.

Ketley CE, Holt RD (1993). Visual and radiographic diagnosis of occlusal caries in first permanent molars and in second primary molars. Br Dent J; 174(10):364-70.

Khan EA, Tyndall DA, Caplan D (2004). Extraoral imaging for proximal caries detection: Bitewings vs scanogram. Oral Surg Oral Med Oral Pathol Oral Radiol Endod; 98(6):730-7.

Khan EA, Tyndall DA, Ludlow JB, Caplan D (2005). Proximal caries detection: Sirona Sidexis versus Kodak Ektaspeed Plus. Gen Dent; 53(1):43-8.

Kidd EA, Banerjee A, Ferrier S, Longbottom C, Nugent Z (2003). Relationships between a clinical-visual scoring system and two histological techniques: a laboratory study on occlusal and approximal carious lesions. Caries Res; 37(2):125-9.

Kielbassa AM, Paris S, Lussi A, Meyer-Lueckel H (2006). Evaluation of cavitations in proximal caries lesions at various magnification levels in vitro. Journal of Dentistry; 34(10):817-822.

Ko HY, Kang SM, Kim HE, Kwon HK, Kim BI (2015). Validation of quantitative light-induced fluorescence-digital (QLF-D) for the detection of approximal caries in vitro. J Dent; 43(5):568-75.

Koob A, Sanden E, Hassfeld S, Staehle HJ, Eickholz P (2004). Effect of digital filtering on the measurement of the depth of proximal caries under different exposure conditions. Am J Dent; 17(6):388-93.

Krzyzostaniak J, Kulczyk T, Czarnecka B, Surdacka A (2015). A comparative study of the diagnostic accuracy of cone beam computed tomography and intraoral radiographic modalities for the detection of noncavitated caries. Clin Oral Investig; 19(3):667-72.

Kuhnisch J, Sochtig F, Pitchika V, Laubender R, Neuhaus KW, Lussi A, Hickel R (2016). In vivo validation of near-infrared light transillumination for interproximal dentin caries detection. Clin Oral Investig; 20(4):821-9.

Kutcher MJ, Kalathingal S, Ludlow JB, Abreu Jr M, Platin E (2006). The effect of lighting conditions on caries interpretation with a laptop computer in a clinical setting. Oral Surgery, Oral Medicine, Oral Pathology, Oral Radiology and Endodontology; 102(4):537-543.

Laitala ML, Piipari L, Sampi N, Korhonen M, Pesonen P, Joensuu T, Anttonen V (2017). Validity of Digital Imaging of Fiber-Optic Transillumination in Caries Detection on Proximal Tooth Surfaces. Int J Dent; 2017:8289636.

Li G, Yoshiura K, Welander U, Shi XQ, McDavid WD (2002). Detection of approximal caries in digital radiographs before and after correction for attenuation and visual response. An in vitro study. Dentomaxillofac Radiol; 31(2):113-6.

Li G, Sanderink GC, Berkhout WE, Syriopoulos K, van der Stelt PF (2007). Detection of proximal caries in vitro using standard and task-specific enhanced images from a storage phosphor plate system. Caries Res; 41(3):231-4.

Li G, Berkhout WER, Sanderink GCH, Martins M, Van Der Stelt PF (2008). Detection of in vitro proximal caries in storage phosphor plate radiographs scanned with different resolutions. Dentomaxillofacial Radiology; 37(6):325-329.

Li G, Qu Xm, Chen Y, Zhang J, Zhang Zy, Ma Xc (2010). Diagnostic accuracy of proximal caries by digital radiographs: an in vivo and in vitro comparative study. Oral Surgery, Oral Medicine, Oral Pathology, Oral Radiology and Endodontology; 109(3):463-467.

Li Y, Ye WP, Li YJ, Yu Y (2006). [Establishment and evaluation of a computer-based software system for detection of initial approximal caries]. Zhonghua Kou Qiang Yi Xue Za Zhi; 41(3):164-7.

Llena-Puy C, Forner L (2005). A clinical and radiographic comparison of caries diagnosed in approximal surfaces of posterior teeth in a low-risk population of 14-year-old children. Oral Health Prev Dent; 3(1):47-52.

Ludlow JB, Platin E, Delano EO, Clifton L (1997). The efficacy of caries detection using three intraoral films under different processing conditions. J Am Dent Assoc; 128(10):1401-8.

Ludlow JB, Abreu Jr M (1999). Performance of film, desktop monitor and laptop displays in caries detection. Dento maxillo facial radiology; 28(1):26-30.

Ludlow JB, Abreu Jr M, Mol A (2001). Performance of a new F-speed film for caries detection. Dento maxillo facial radiology; 30(2):110-113.

Lussi A, Hack A, Hug I, Heckenberger H, Megert B, Stich H (2006). Detection of approximal caries with a new laser fluorescence device. Caries Research; 40(2):97-103.

Lussi A, Hellwig E (2006). Performance of a new laser fluorescence device for the detection of occlusal caries in vitro. Journal of Dentistry; 34(7):467-471.

Madalli VB, Annigeri RG, Basavaraddi SM (2014). The evaluation of effect of developer age in the detection of approximal caries using three speed dental x-ray films: an in-vitro study. J Clin Diagn Res; 8(3):236-9.

Maia AMA, Karlsson L, Margulis W, Gomes ASL (2011). Evaluation of two imaging techniques: Near-infrared transillumination and dental radiographs for the detection of early approximal enamel caries. Dentomaxillofacial Radiology; 40(7):429-433.

Marsh PD, Featherstone A, McKee AS, Hallsworth AS, Robinson C, Weatherell JA, Newman HN, Pitter AF (1989). A microbiological study of early caries of approximal surfaces in schoolchildren. J Dent Res; 68(7):1151-4.

Marthaler TM (1966). A standardized system of recording dental conditions. Helv Odontol Acta; 10(1):1-18.

Matalon S, Feuerstein O, Kaffe I (2003). Diagnosis of approximal caries: bite-wing radiology versus the Ultrasound Caries Detector. An in vitro study. Oral Surg Oral Med Oral Pathol Oral Radiol Endod; 95(5):626-31.

Matalon S, Feuerstein O, Calderon S, Mittleman A, Kaffe I (2007). Detection of cavitated carious lesions in approximal tooth surfaces by ultrasonic caries detector. Oral Surgery, Oral Medicine, Oral Pathology, Oral Radiology, and Endodontics; 103(1):109-113.

Matsuda Y, Hanazawa T, Seki K, Araki K, Okano T (2002). Comparison between RVG UI sensor and Kodak Insight film for detection of incipient proximal caries. Oral Radiology; 18(2):105-111.

Menem R, Barngkgei I, Beiruti N, Al Haffar I, Joury E (2017). The diagnostic accuracy of a laser fluorescence device and digital radiography in detecting approximal caries lesions in posterior permanent teeth: an in vivo study. Lasers Med Sci; 32(3):621-628.

Mialhe FL, Pereira AC, Pardi V, de Castro Meneghim M (2003). Comparison of three methods for detection of carious lesions in proximal surfaces versus direct visual examination after tooth separation. J Clin Pediatr Dent; 28(1):59-62.
Mileman PA, van der Weele LT (1990). Accuracy in radiographic diagnosis: Dutch practitioners and dental caries. J Dent; 18(3):130-6.
Mileman PA, van den Hout WB (2002). Comparing the accuracy of Dutch dentists and dental students in the radiographic diagnosis of dentinal caries. Dentomaxillofac Radiol; 31(1):7-14.
Milosavljevic A, Westerberg J, Hellen-Halme K (2016). Diagnostic accuracy of carious lesions in digital radiographs at a public dental clinic - can it be improved by optimizing viewing conditions and further education? Swed Dent J; 40(2):235-242.
Minston W, Li G, Wennberg R, Nasstrom K, Shi XQ (2013). Comparison of diagnostic performance on approximal caries detection among Swedish and Chinese senior dental students using analogue and digital radiographs. Swed Dent J; 37(2):79-85.
Miri S, Mehralizadeh S, Sadri D, Motamedi MR, Soltani P (2015). The efficacy of the reverse contrast mode in digital radiography for the detection of proximal dentinal caries. Imaging Sci Dent; 45(3):141-5.
Mitropoulos P, Rahiotis C, Stamatakis H, Kakaboura A (2010). Diagnostic performance of the visual caries classification system ICDAS II versus radiography and micro-computed tomography for proximal caries detection: An in vitro study. Journal of Dentistry; 38(11):859-867.
Moystad A, Svanaes DB, Risnes S, Larheim TA, Grondahl HG (1996). Detection of approximal caries with a storage phosphor system. A comparison of enhanced digital images with dental X-ray film. Dentomaxillofac Radiol; 25(4):202-6.
Moystad A, Svanaes DB, van der Stelt PF, Grondahl HG, Wenzel A, van Ginkel FC, Kullendorff B, Hintze H, Larheim TA (2003). Comparison of standard and task-specific enhancement of Digora storage phosphor images for approximal caries diagnosis. Dentomaxillofac Radiol; 32(6):390-6.
Nair MK, Nair UP (2001). An in-vitro evaluation of Kodak Insight and Ektaspeed Plus film with a CMOS detector for natural proximal caries: ROC analysis. Caries Res; 35(5):354-9.
Nascimento EH, Gaeta-Araujo H, Vasconcelos KF, Freire BB, Oliveira-Santos C, Haiter-Neto F, Freitas DQ (2018). Influence of brightness and contrast adjustments on the diagnosis of proximal caries lesions. Dentomaxillofac Radiol; 47(8):20180100.
Neuhaus KW, Ciucchi P, Rodrigues JA, Hug I, Emerich M, Lussi A (2015). Diagnostic performance of a new red light LED device for approximal caries detection. Lasers Med Sci; 30(5):1443-7.
Nikneshan S, Abbas FM, Sabbagh S (2015). Detection of proximal caries using digital radiographic systems with different resolutions. Indian J Dent Res; 26(1):5-10.
Noar SJ, Smith BGN (1990). Diagnosis of caries and treatment decisions in approximal surfaces of posterior teeth in vitro. Journal of Oral Rehabilitation; 17(3):209-218.
Obry-Musset AM, Cahen PM, Turlot JC, Frank RM (1988). Approximal caries diagnosis in epidemiological studies: transillumination or bitewing radiographs? J Biol Buccale; 16(1):13-7.
Okano T, Huang HJ, Nakamura T (1985). Diagnostic accuracy on detection of proximal enamel lesions in nonscreen radiographic performance. Oral Surg Oral Med Oral Pathol; 59(5):543-7.
Otis LL, Sherman RG (2005). Assessing the accuracy of caries diagnosis via radiograph. Film versus print. J Am Dent Assoc; 136(3):323-30.
Ozkan G, Guzel KGU (2017). Clinical evaluation of near-infrared light transillumination in approximal dentin caries detection. Lasers Med Sci; 32(6):1417-1422.
Ozsevik AS, Kararslan ES, Aktan AM, Bozdemir E, Cebe F, Sari F (2015). Effect of Different Contact Materials on Approximal Caries Detection by Laser Fluorescence and Light-Emitting Diode Devices. Photomed Laser Surg; 33(10):492-7.
Pabla T, Ludlow JB, Tyndall DA, Platin E, Abreu M, Jr. (2003). Effect of data compression on proximal caries detection: observer performance with DenOptix photostimulable phosphor images. Dentomaxillofac Radiol; 32(1):45-9.

Pakkala T, Kuusela L, Ekholm M, Wenzel A, Haiter-Neto F, Kortesniemi M (2012). Effect of varying displays and room illuminance on caries diagnostic accuracy in digital dental radiographs. Caries Research; 46(6):568-574.

Peers A, Hill FJ, Mitropoulos CM, Holloway PJ (1993). Validity and reproducibility of clinical examination, fibre-optic transillumination, and bite-wing radiology for the diagnosis of small approximal carious lesions: an in vitro study. Caries Res; 27(4):307-11.

Peker I, Toraman Alkurt M, Altunkaynak B (2007). Film tomography compared with film and digital bitewing radiography for proximal caries detection. Dentomaxillofacial Radiology; 36(8):495-499.

Peker I, Toraman Alkurt M, Bala O, Altunkaynak B (2009). The efficiency of operating microscope compared with unaided visual examination, conventional and digital intraoral radiography for proximal caries detection. Int J Dent; 2009:986873.

Pitts NB (1984). Systems for grading approximal carious lesions and overlaps diagnosed from bitewing radiographs. Proposals for future standardization. Community Dent Oral Epidemiol; 12(2):114-22.

Pitts NB, Rimmer PA (1992). An in vivo comparison of radiographic and directly assessed clinical caries status of posterior approximal surfaces in primary and permanent teeth. Caries Res; 26(2):146-52.

Pontual AA, De Melo DP, De Almeida SM, Boscolo FN, Haiter Neto F (2010). Comparison of digital systems and conventional dental film for the detection of approximal enamel caries. Dentomaxillofacial Radiology; 39(7):431-436.

Qu X, Li G, Zhang Z, Ma X (2011). Detection accuracy of in vitro approximal caries by cone beam computed tomography images. European Journal of Radiology; 79(2):e24-e27.

Raghav M, Sontakke S, Karjodkar F, Wenzel A (2014). Clinical cavitation and radiographic lesion depth in proximal surfaces in an Indian population. Acta odontologica Scandinavica; 72(8):1084-1088.

Reddy VV, Sugandhan S (1994). A comparison of bitewing radiography and fibreoptic illumination as adjuncts to the clinical identification of approximal caries in primary and permanent molars. Indian J Dent Res; 5(2):59-64.

Ricketts DN, Whaites EJ, Kidd EA, Brown JE, Wilson RF (1997). An evaluation of the diagnostic yield from bitewing radiographs of small approximal and occlusal carious lesions in a low prevalence sample in vitro using different film types and speeds. Br Dent J; 182(2):51-8.

Ritter AV, Ramos MD, Astorga F, Shugars DA, Bader JD (2013). Visual-tactile versus radiographic caries detection agreement in caries-active adults. J Public Health Dent; 73(3):252-60.

Rocha AS, Almeida SM, Boscolo FN, Haiter Neto F (2005). Interexaminer agreement in caries radiographic diagnosis by conventional and digital radiographs. J Appl Oral Sci; 13(4):329-33.

Rockenbach MI, Veeck EB, da Costa NP (2008). Detection of proximal caries in conventional and digital radiographs: an in vitro study. Stomatologija / issued by public institution "Odontologijos studija" .. [et al.]. 10(4):115-120.

Russell M, Pitts NB (1993). Radiovisiographic diagnosis of dental caries: initial comparison of basic mode videoprints with bitewing radiography. Caries Res; 27(1):65-70.

Safi Y, Shamloo Mahmoudi N, Aghdasi MM, Eslami Manouchehri M, Rahimian R, Valizadeh S, Vasegh Z, Azizi Z (2015). Diagnostic accuracy of Cone Beam Computed Tomography, conventional and digital radiographs in detecting interproximal caries. J Med Life; 8(Spec Iss 3):77-82.

Sansare K, Raghav M, Sontakke S, Karjodkar F, Wenzel A (2014a). Clinical cavitation and radiographic lesion depth in proximal surfaces in an Indian population. Acta Odontol Scand; 72(8):1084-8.

Sansare K, Singh D, Sontakke S, Karjodkar F, Saxena V, Frydenberg M, Wenzel A (2014b). Should cavitation in proximal surfaces be reported in cone beam computed tomography examination? Caries Res; 48(3):208-13.

Scarfe WC, Langlais RP, Nummikoski P, Dove SB, McDavid WD, Deahl ST, Yuan CH (1994). Clinical comparison of two panoramic modalities and posterior bite-wing radiography in the detection of proximal dental caries. Oral Surg Oral Med Oral Pathol; 77(2):195-207.

Schneiderman A, Elbaum M, Shultz T, Keem S, Greenebaum M, Driller J (1997). Assessment of dental caries with Digital Imaging Fiber-Optic TransIllumination (DIFOTI): in vitro study. Caries Res; 31(2):103-10.

Schulte AG, Wittchen A, Stachniss V, Jacquet W, Bottenberg P (2008). Approximal caries diagnosis after data import from different digital radiography systems: Interobserver agreement and comparison to histological hard-tissue sections. Caries Research; 42(1):57-61.

Schulze RKW, Richter A, D'Hoedt B (2008). The effect of wavelet and discrete cosine transform compression of digital radiographs on the detection of subtle proximal caries. Caries Research; 42(5):334-339.

Schulze RKW, Grimm S, Schulze D, Voss K, Keller HP, Wedel M (2011). Diagnostic yield of ink-jet prints from digital radiographs for the assessment of approximal carious lesions: ROC-analysis. European Journal of Radiology; 79(2):277-282.

Senel B, Kamburoglu K, Ucok O, Yuksel SP, Ozen T, Avsever H (2010). Diagnostic accuracy of different imaging modalities in detection of proximal caries. Dentomaxillofacial Radiology; 39(8):501-511.

Shi XQ, Tranaeus S, Angmar-Mansson B (2001). Comparison of QLF and DIAGNOdent for quantification of smooth surface caries. Caries Res; 35(1):21-6.

Shi XQ, Li G (2009). Detection accuracy of approximal caries by black-and-white and color-coded digital radiographs. Oral Surgery, Oral Medicine, Oral Pathology, Oral Radiology and Endodontology; 107(3):433-436.

Shimada Y, Nakagawa H, Sadr A, Wada I, Nakajima M, Nikaido T, Otsuki M, Tagami J, Sumi Y (2014). Noninvasive cross-sectional imaging of proximal caries using swept-source optical coherence tomography (SS-OCT) in vivo. J Biophotonics; 7(7):506-13.

Svanaes DB, Moystad A, Risnes S, Larheim TA, Grondahl HG (1996). Intraoral storage phosphor radiography for approximal caries detection and effect of image magnification: comparison with conventional radiography. Oral Surg Oral Med Oral Pathol Oral Radiol Endod; 82(1):94-100.

Svanaes DB, Moystad A, Larheim TA (2000). Approximal caries depth assessment with storage phosphor versus film radiography. Evaluation of the caries-specific Oslo enhancement procedure. Caries Res; 34(6):448-53.

Svenson B, Lindvall AM, Grondahl HG (1993). A comparison of a new dental X-ray film, Agfa Gevaert Dentus M4, with Kodak Ektaspeed and Ultraspeed dental X-ray films. Dentomaxillofac Radiol; 22(1):7-12.

Syriopoulos K, Velders XL, Sanderink GC, van Ginkel FC, van Amerongen JP, van der Stelt PF (1999). The effect of developer age on the detection of approximal caries using three dental films. Dentomaxillofac Radiol; 28(4):208-13.

Syriopoulos K, Sanderink GC, Velders XL, van der Stelt PF (2000). Radiographic detection of approximal caries: a comparison of dental films and digital imaging systems. Dento maxillo facial radiology; 29(5):312-318.

Tagtekin DA, Ozyoney G, Baseren M, Ando M, Hayran O, Alpar R, Gokalp S, Yanikoglu FC, Stookey GK (2008). Caries detection with DIAGNOdent and ultrasound. Oral Surgery, Oral Medicine, Oral Pathology, Oral Radiology and Endodontology; 106(5):729-735.

Tonkaboni A, Saffarpour A, Aghapourzangeneh F, Fard MJK (2019). Comparison of diagnostic effects of infrared imaging and bitewing radiography in proximal caries of permanent teeth. Lasers Med Sci; 34(5):873-879.

Tracy KD, Dykstra BA, Gakenheimer DC, Scheetz JP, Lacina S, Scarfe WC, Farman AG (2011). Utility and effectiveness of computer-aided diagnosis of dental caries. General Dentistry; 59(2):136-144.

Tsuchida R, Araki K, Okano T (2007). Evaluation of a limited cone-beam volumetric imaging system: comparison with film radiography in detecting incipient proximal caries. Oral Surgery, Oral Medicine, Oral Pathology, Oral Radiology and Endodontology; 104(3):412-416.

Tyndall DA, Ludlow JB, Platin E, Nair M (1998). A comparison of Kodak Ektaspeed Plus film and the Siemens Sidexis digital imaging system for caries detection using receiver operating characteristic analysis. Oral Surg Oral Med Oral Pathol Oral Radiol Endod; 85(1):113-8.

Vaarkamp J, ten Bosch J, Verdonschot EH, Huysmans MC (1997a). Wavelength-dependent fibre-optic transillumination of small approximal caries lesions: the use of a dye, and a comparison to bitewing radiography. Caries Res; 31(3):232-7.

Vaarkamp J, Ten Bosch JJ, Verdonschot EH, Tranaeus S (1997b). Quantitative diagnosis of small approximal caries lesions utilizing wavelength-dependent fiber-optic transillumination. J Dent Res; 76(4):875-82.
Vaarkamp J, Ten Bosch JJ, Verdonschot EH, Bronkhorst EM (2000). The real performance of bitewing radiography and fiber-optic transillumination in approximal caries diagnosis. Journal of Dental Research; 79(10):1747-1751.
Valizadeh S, Tavakoli MA, Zarabian T, Esmaeili F (2009). Diagnostic accuracy of digitized conventional radiographs by camera and scanner in detection of proximal caries. J Dent Res Dent Clin Dent Prospects; 3(4):126-31.
Verdonschot EH, van de Rijke JW, Brouwer W, ten Bosch JJ, Truin GJ (1991). Optical quantitation and radiographic diagnosis of incipient approximal caries lesions. Caries Res; 25(5):359-64.
Verdonschot EH, Kuijpers JM, Polder BJ, De Leng-Worm MH, Bronkhorst EM (1992). Effects of digital grey-scale modification on the diagnosis of small approximal carious lesions. J Dent; 20(1):44-9.
Vieira MS, Nogueira CP, Silva MA, Bauer JR, Maia Filho EM (2015). In vitro evaluation of proximal carious lesions using digital radiographic systems. ScientificWorldJournal; 2015:631508.
Vivek V, Thomas S, Nair BJ, Vineet AD, Thomas J, Ranimol P, Vijayan AK (2015). Comparison of Diagnostic Ability of Storage Phosphor Plate in Detecting Proximal Caries with Direct Measurement by Stereomicroscope: A Pilot Study. Clin Pract; 5(3):763.
Wenzel A, Borg E, Hintze H, Grondahl HG (1995). Accuracy of caries diagnosis in digital images from charge-coupled device and storage phosphor systems: an in vitro study. Dentomaxillofac Radiol; 24(4):250-4.
Wenzel A, Hintze H (1999). Comparison of microscopy and radiography as gold standards in radiographic caries diagnosis. Dentomaxillofac Radiol; 28(3):182-5.
Wenzel A, Hintze H, Kold LM, Kold S (2002). Accuracy of computer-automated caries detection in digital radiographs compared with human observers. Eur J Oral Sci; 110(3):199-203.
Wenzel A, Haiter-Neto F, Gotfredsen E (2007a). Influence of spatial resolution and bit depth on detection of small caries lesions with digital receptors. Oral Surgery, Oral Medicine, Oral Pathology, Oral Radiology and Endodontology; 103(3):418-422.
Wenzel A, Haiter-Neto F, Gotfredsen E (2007b). Risk factors for a false positive test outcome in diagnosis of caries in approximal surfaces: Impact of radiographic modality and observer characteristics. Caries Research; 41(3):170-176.
White SC, Gratt BM, Bauer JG (1988). A clinical comparison of xeroradiography and film radiography for the detection of proximal caries. Oral Surg Oral Med Oral Pathol; 65(2):242-8.
White SC, Yoon DC (1997). Comparative performance of digital and conventional images for detecting proximal surface caries. Dentomaxillofac Radiol; 26(1):32-8.
White SC, Yoon DC (2000). Comparison of sensitometric and diagnostic performance of two films. Compend Contin Educ Dent; 21(6):530-2, 534, 536 passim.
Wojtowicz PA, Brooks SL, Hasson H, Kerschbaum WE, Eklund SA (2003). Radiographic detection of approximal caries: a comparison between senior dental students and senior dental hygiene students. J Dent Hyg; 77(4):246-51.
Wong A, Monsour PA, Moule AJ, Basford KE (2002). A comparison of Kodak Ultraspeed and Ektaspeed plus dental X-ray films for the detection of dental caries. Aust Dent J; 47(1):27-9.
Xavier CRG, Araujo-Pires AC, Poleti ML, Rubira-Bullen IRF, Ferreira Jr O, Capelozza ALA (2011). Evaluation of proximal caries in images resulting from different modes of radiographic digitalization. Dentomaxillofacial Radiology; 40(6):338-343.
Yoon HI, Yoo MJ, Park EJ (2017). Detection of proximal caries using quantitative light-induced fluorescence-digital and laser fluorescence: a comparative study. J Adv Prosthodont; 9(6):432-438.
Young DA, Featherstone JDB (2005). Digital Imaging Fiber-Optic Trans-Illumination, F-speed radiographic film and depth of approximal lesions. Journal of the American Dental Association; 136(12):1682-1687.

Young SM, Lee JT, Hodges RJ, Chang TL, Elashoff DA, White SC (2009). A comparative study of high-resolution cone beam computed tomography and charge-coupled device sensors for detecting caries. Dentomaxillofacial Radiology; 38(7):445-451.

Zangooei Booshehry M, Davari A, Ezoddini Ardakani F, Rashidi Nejad MR (2010). Efficacy of application of pseudocolor filters in the detection of interproximal caries. J Dent Res Dent Clin Dent Prospects; 4(3):79-82.

Zayet MK, Helaly YR, Eiid SB (2014). Effect of changing the kilovoltage peak on radiographic caries assessment in digital and conventional radiography. Imaging Sci Dent; 44(3):199-205.

Zhang ZL, Qu XM, Li G, Zhang ZY, Ma XC (2011). The detection accuracies for proximal caries by cone-beam computerized tomography, film, and phosphor plates. Oral Surgery, Oral Medicine, Oral Pathology, Oral Radiology and Endodontology; 111(1):103-108.
